# Correlating Structure with Spectroscopy in Ascorbate Peroxidase Compound II

**DOI:** 10.1021/jacs.3c13169

**Published:** 2024-03-26

**Authors:** Mursaleem Ansari, Sinjini Bhattacharjee, Dimitrios A. Pantazis

**Affiliations:** Max-Planck-Institut für Kohlenforschung, Kaiser-Wilhelm-Platz 1, Mülheim an der Ruhr 45470, Germany

## Abstract

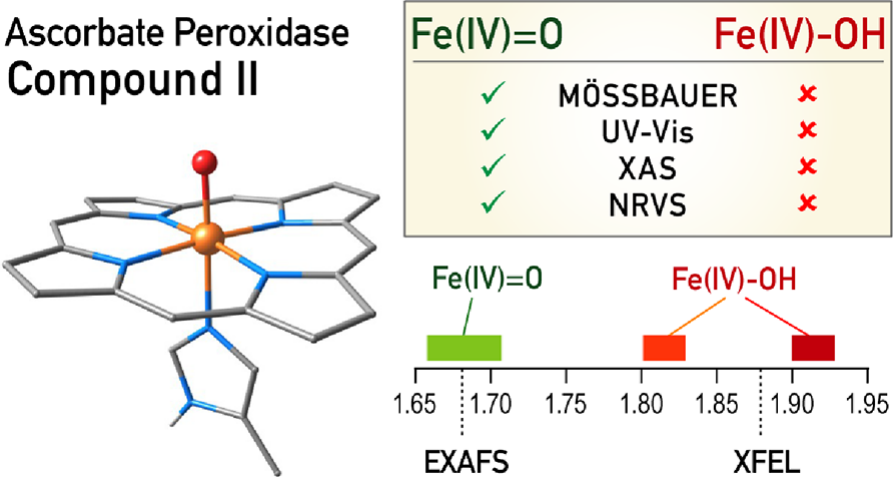

Structural and spectroscopic investigations of compound II in ascorbate peroxidase (APX) have yielded conflicting conclusions regarding the protonation state of the crucial Fe(IV) intermediate. Neutron diffraction and crystallographic data support an iron(IV)-hydroxo formulation, whereas Mössbauer, X-ray absorption (XAS), and nuclear resonance vibrational spectroscopy (NRVS) studies appear consistent with an iron(IV)-oxo species. Here we examine APX with spectroscopy-oriented QM/MM calculations and extensive exploration of the conformational space for both possible formulations of compound II. We establish that irrespective of variations in the orientation of a vicinal arginine residue and potential reorganization of proximal water molecules and hydrogen bonding, the Fe–O distances for the oxo and hydroxo forms consistently fall within distinct, narrow, and nonoverlapping ranges. The accuracy of geometric parameters is validated by coupled-cluster calculations with the domain-based local pair natural orbital approach, DLPNO-CCSD(T). QM/MM calculations of spectroscopic properties are conducted for all structural variants, encompassing Mössbauer, optical, X-ray absorption, and X-ray emission spectroscopies and NRVS. All spectroscopic observations can be assigned uniquely to an Fe(IV)=O form. A terminal hydroxy group cannot be reconciled with the spectroscopic data. Under no conditions can the Fe(IV)=O distance be sufficiently elongated to approach the crystallographically reported Fe–O distance. The latter is consistent only with a hydroxo species, either Fe(IV) or Fe(III). Our findings strongly support the Fe(IV)=O formulation of APX-II and highlight unresolved discrepancies in the nature of samples used across different experimental studies.

## 1 Introduction

**Figure 1:**
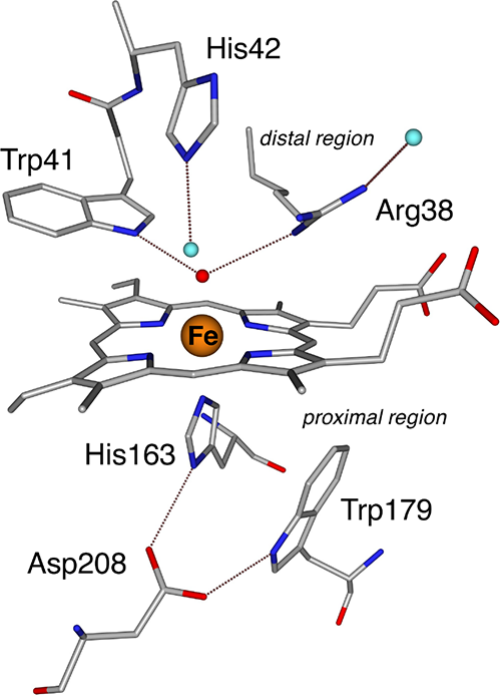
Active site of ascorbate peroxidase as deduced from structural studies.^17,18^ Waters are represented in light blue, ferryl oxygen is depicted in red, and possible hydrogen bonding interactions are depicted as dotted lines. Note that the Arg38 position is not fully resolved crystallographically^18^ and that the water shown on the right can also occupy an “internal” position.

Heme-containing peroxidases oxidize a wide variety of substrates by reacting with hydrogen peroxide.^1,2^ Among the well-known heme peroxidases are cytochrome c peroxidase (CCP),^3,4^ lignin peroxidase,^5^ ascorbate peroxidase (APX),^6,7^ peanut peroxidase,^8^ and horseradish peroxidase (HRP).^9^ APX, the subject of the present study, is an essential peroxidase in various organisms that plays a significant role in protecting cells from oxidative stress.^10,11^ It is involved in the detoxification of hydrogen peroxide^12^ utilizing ascorbate (vitamin C) as a reducing agent,^13,14^ thus contributing to maintaining cellular redox balance and preventing oxidative damage to essential biomolecules.^15,16^ The active site of APX and its immediate environment are shown in Figure 1.^17,18^ In all structurally characterized peroxidases, the histidine (His163) and aspartate (Asp208) on the “proximal” heme side consistently occupy the shown positions. However, the proximal tryptophan (Trp179 in Figure 1) is unique to CCP and APX, as most other peroxidases feature phenylalanine in this position.^4^ On the opposite side, the axial oxygen-derived ligand in APX is surrounded by a hydrogen-bonding tryptophan (Trp41) as well as a histidine (His42) and arginine (Arg38), the latter two connected with the axial group via intermediary water ligands.

Many heme-containing systems employ ferryl heme in catalysis.^19,20^ The term ferryl refers to an oxidation state of iron elevated by one (Fe(IV), known as “compound II”) or two equivalents (Fe(V) or more accurately Fe(IV)-L^•^, known as “compound I”) compared to the ferric Fe(III) state. In compound I, the second oxidative equivalent is situated either on the porphyrin ring or, in certain cases, on a protein-based radical.^21^ Thiolate-ligated heme systems such as cytochrome P450 are able to activate C–H bonds,^22,23^ presumably because the strong electron donation from the axial thiolate ligand enhances the reactivity of the primary oxidant compound I toward C–H activation while also safeguarding from oxidative damage.^24−26^ In contrast, peroxidases with histidine ligands, like APX, generally follow sequential one-electron oxidation and are not recognized as capable of activating C–H bonds.^22,27^ Owing to the fact that histidine is less electron-donating than the thiolate, His-ligated compound II species are believed to be more electrophilic.^25^ The nature of compound II has been established for many enzymes but remains contentious for APX. The controversy centers around the protonation state of the Fe(IV) intermediate, i.e., Fe(IV)–OH or Fe(IV)=O.^17,28−31^ Whether compound II in APX (hereafter APX-II) is an oxo or hydroxo species has crucial implications for understanding the principles of metal-oxo catalyzed C–H activation.^31^

The origin of the controversy lies in the discrepancy between structural and spectroscopic information on the nature of APX-II.^28,30,32−37^ A neutron diffraction study of APX-II documented positive nuclear density consistent with an OH ligand and supported an Fe–O distance of ca. 1.88 Å, a bond length considered consistent with an Fe(IV)-hydroxide species.^17^ In 2021 an X-ray crystallographic study using an X-ray free electron laser (XFEL) source supported a very similar bond length of ca. 1.87 Å, further advocating the assignment of APX-II as Fe(IV)–OH.^18^ Although this distance-based interpretation is reasonable, it should be kept in mind that neither approach directly probes the oxidation state of the iron in the crystal; this requires complementary techniques that may or may not be applied *in crystallo* and in the same sample.^17^ In contrast to the above, Mössbauer and X-ray absorption spectroscopy (XAS) experiments that utilized extended X-ray absorption fine structure (EXAFS) and analysis of the Fe pre-edge region supported that APX-II is an Fe(IV)-oxo species with an estimated Fe–O bond length of ca. 1.68 Å.^31^ Among others, this was supported by the similarity of the pre-edge region of APX-II with other known Fe(IV)=O species^25,35^ and by the impossibility of fitting the EXAFS with the assumption of a hydroxide species.^31^ Data obtained by a subsequent nuclear resonance vibrational spectroscopy (NRVS) study evidenced vibrational features consistent with similarly short Fe–O distances of ca. 1.67–1.69 Å and hence further supported the Fe(IV)=O formulation of APX-II.^38^

The two distinct lines of evidence are irreconcilable, except if local, as yet undefined elements of the active site create an extraordinary electronic structure situation that allows unprecedented flexibility and, hence, bond length overlap between different protonation or even oxidation states of the Fe–O group.^18^ This problem calls for detailed spectroscopic-oriented multiscale modeling of the enzyme. Quantum chemical studies can offer important insights by mapping precise connections between geometric features and electronic/spectroscopic properties. Such approaches have been used extensively in the study of various heme- and nonheme iron systems,^29,39−47^ and in investigations of compound I and II intermediates in particular. However, there has been no systematic effort so far to address the above-described controversy surrounding APX-II. Here we approach the problem using multiscale calculations in the form of quantum mechanics/molecular mechanics (QM/MM) on an all-atom MM model of APX with a sufficiently large QM region to encompass the heme iron site and all relevant second-sphere residues that may affect the bonding environment of the Fe–O(H) unit. Building on extensive geometry optimizations, supported by coupled-cluster validation of crucial bonding parameters, we explore structure–spectroscopy correlations using the full range of experimentally reported spectroscopic techniques. Our results allow us to establish explicit relationships between structural features and spectroscopic properties for APX-II. By evaluating a wide range of alternatives, we definitively exclude the possibility of bond length overlap between different protonation and oxidation state formulations of APX-II. We show that only Fe(IV)=O models can be consistent with the spectroscopic data and suggest that the precise nature of the species giving rise to the “long” bond lengths reported by crystallography needs to be carefully re-evaluated.

## 2 Methodology

### 2.1 Construction of the Protein Model

**Figure 2:**
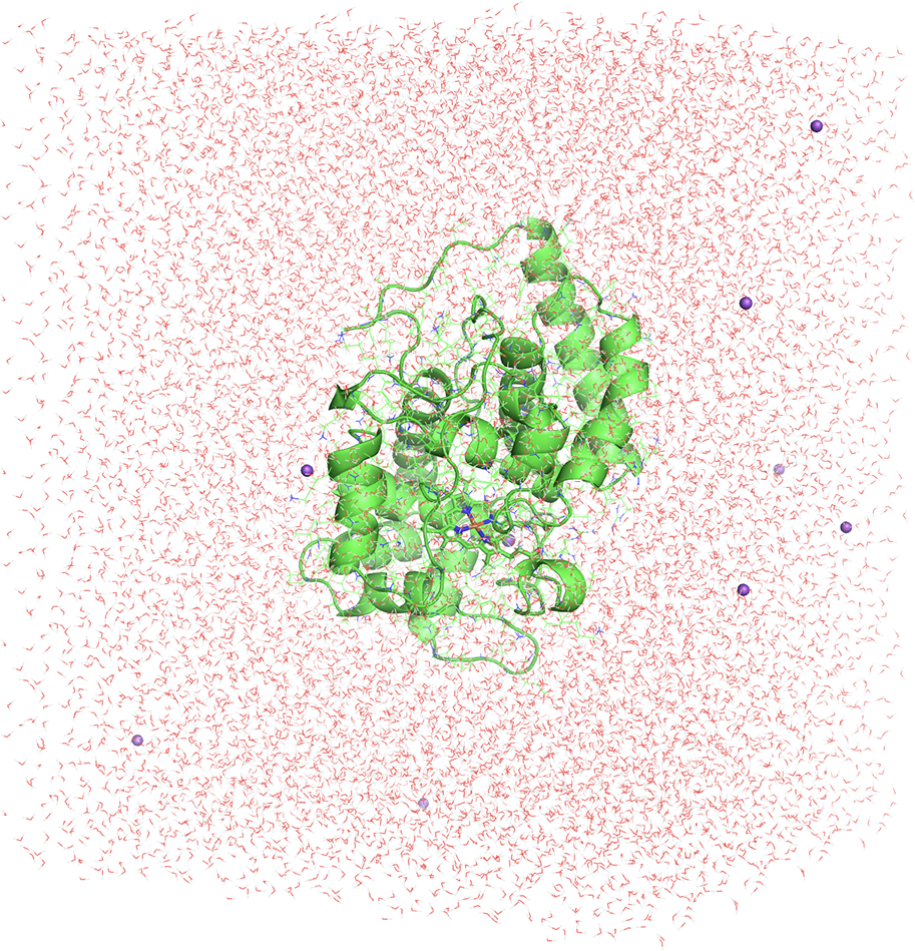
Depiction of the complete ascorbate peroxidase model with the water box (96 × 81 × 77 Å) and sodium counterions employed in classical MD simulations and QM/MM calculations.

The complete molecular mechanics setup is based on the crystal structure of soybean APX using the X-ray free electron laser at SACLA at an atomic resolution of 1.5 Å (PDB ID: 7BI1).^18^ The enzyme is composed of a single chain and is almost entirely helical. The whole protein was modeled classically using AmberTools20.^48^ All 249 amino acid residues, 1 heme, 1 K^+^, and 566 waters present in the crystal structure were retained. Throughout this work, we follow the nomenclature used in the 7BI1 crystal structure for residue labeling. The protonation states of titratable residues were determined and missing hydrogens were added using the H++ web server.^49^ All arginine (Arg) and tryptophan (Trp) residues were modeled in their protonated states, whereas histidines (His) were modeled as being singly protonated. The protonation states of all residues at the active site were checked and determined manually. Specifically, the His163 directly ligated to Fe was initially protonated at the Nδ position (HID in Amber nomenclature); all oxygens at the active site were protonated and initially modeled as waters. We added eight Na^+^ ions to maintain charge neutrality. The complete system is further solvated (at least 15 Å around the solute), resulting in 50,832 atoms and an overall dimension of 96 × 81 × 77 Å for the simulation box (Figure 2). The standard protein residues and waters are described with the Amberff14SB force field^50^ and TIP3P model, respectively.^51^ The bonded parameters for the heme active site are obtained from the literature,^52^ whereas the MK-RESP (Merz–Kollman restrained electrostatic potential) charges^53,54^ were derived for both Fe(III) and Fe(IV) oxidation states and the corresponding axial ligand. The initial setup set Fe to Fe(III) and the axial ligand to H_2_O, which enabled easy subsequent modifications for generation of QM/MM variants. The partial charges (see SI) were first computed at the B3LYP/6-31G* level of theory,^55^ and RESP fitting was performed in Multiwfn.^56^ Joung–Cheatham parameters compatible with TIP3P models were used for the K^+^ and Na^+^ ions.^51^

The complete system was systematically minimized to remove unfavorable geometric clashes and optimize the side chain positions. First, only hydrogens were minimized in 2000 steps, followed by the solvent (waters), ions, and protein side chains through 10,000 cycles. During the minimization procedure, we applied positional restraints (100 kcal mol^–1^ Å^–2^) on the heme moiety, its axial ligands, and the backbone atoms to maintain the same active site geometry and thus ensure a common starting structure for all QM/MM calculations and to prevent unnatural backbone movements. To ensure minimum deviations from the fold of the crystallographic structure, the protein configuration obtained after the minimization procedure was used for generating the initial structures for all QM/MM calculations. Using a crystallographic-like setup is desirable because our principal goal in this study is to compare with experimental data on the Fe–O(H) unit obtained from studies of the crystal or in general under cryogenic conditions. We point out that crystallographic structures are not necessarily representative of biologically relevant physiological conditions, which will have to be simulated when focusing on mechanistic aspects. Nevertheless, the present structure–property correlations are most meaningful when compared against crystallography; protein conformational changes will, in any case, not affect the spectroscopic signatures of the Fe–O(H) unit because all spectroscopic parameters utilized in our study are essentially local.

Selected QM/MM models chosen as examples of spectroscopically consistent formulations of APX-II were used for further molecular dynamics simulations. In the equilibration phase, the system was heated from 10 to 300 K for 100 ps in the NVT ensemble followed by further equilibration in the NPT ensemble at 300 K for 1000 ps. The temperature during this step was maintained using Langevin dynamics with a collision frequency of 5 ps^–1^. During the equilibration MD, the heme with its axial ligands and backbone atoms were restrained with a reduced force constant of 50 kcal mol^–1^ Å^–2^. Subsequently, the restraints on the backbone atoms were relaxed to 10 kcal mol^–1^ Å^–2^, and a production run was initiated for 50 ns (NPT) with the temperature and pressure set at 303 K and 1 atm. During the entire procedure, the temperature was controlled using a Langevin thermostat with a collision frequency of 1 ps^–1^, and the system pressure was controlled using a Berendsen barostat with anisotropic pressure scaling with a relaxation time of 1 ps. We employed the SHAKE algorithm to constrain the bonds involving hydrogens.^57^ The time step in MD simulations was maintained at 1 fs, and frames were saved every 10 ps. Electrostatic interactions were treated using the Particle Mesh Ewald (PME) approach with a 10 Å cutoff.^58^ The energy minimizations were done using AmberTools20, whereas the equilibration and production MD simulations were performed in the GPU version of the pmemd module (*pmemd.cuda*) of Amber20.

### 2.2 QM/MM Setup

**Figure 3:**
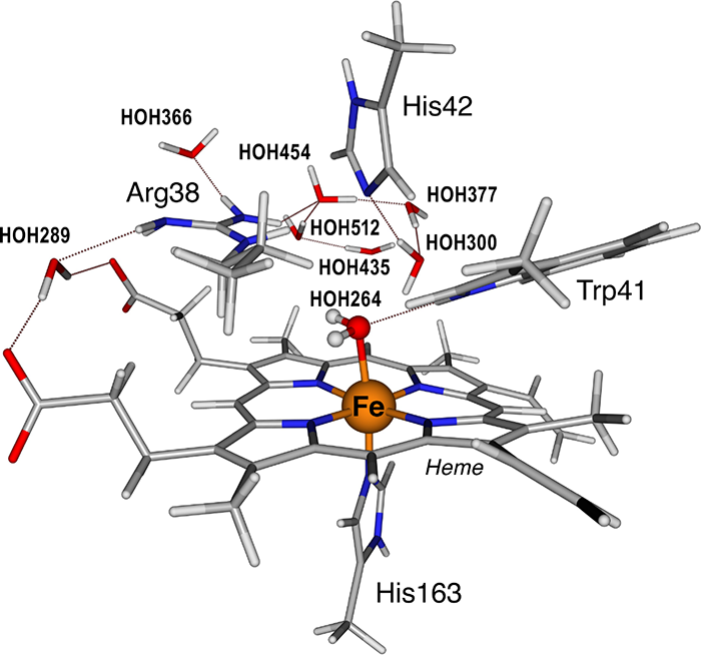
Final selected QM region used in the QM/MM calculations comprises 159 QM atoms.

The final QM/MM setup consisted of the whole protein, the complete water box, and eight Na^+^ counterions to maintain system neutrality. We tested several sizes of the QM regions. Starting from a minimal QM region that only included heme, His163, HOH264 and HOH289, we increased the size in five steps to gradually include the side chain of Arg38, Trp41, His42, Ser160, His167, Arg172, Trp179, Asp208, and a number of closely bound waters (HOH300, HOH353, HOH366, HOH377, HOH289, HOH435, HOH454, and HOH512) (Figure S1). The convergence of critical geometric parameters with the increasing size of the QM region was monitored, and this investigation led to the conclusion that the parameters of interest are stable and no longer change at a size of the QM region that includes the heme, Arg38, Trp41, His42, His163, and a number of closely bound waters (HOH300, HOH366, HOH377, HOH435, HOH454, and HOH512) (Table S1). Therefore, all remaining calculations were performed with this setup. The final QM region used in the production QM/MM optimizations is shown in Figure 3. A large number of models were subsequently created by specific active site modifications for the given QM region, as described in the Results and Discussion section.

In the QM/MM calculations, not only the QM region is allowed to relax but also a large region of MM atoms surrounding it; this is referred to as the “active region” of the QM/MM simulation. All atoms within the active regions (described at either the QM or MM levels) are allowed to move during the optimization, whereas the remaining atoms remain constrained. To determine the size of the active region, all protein residues and water molecules containing even one atom within a certain radius from the QM site are considered “active”. In most cases, we utilized a 3 Å distance criterion, which means that the active region included complete residues and water molecules with at least one atom within 3 Å of the heme. This choice led to approximately 735 active atoms in the QM/MM models. We note that the use of active regions, i.e., of an MM layer that is relaxed using force-field parameters in response to the relaxation of the QM region within a QM/MM run, is important to avoid convergence to new local minima during each new QM/MM optimization.^59^ Overall, following a number of tests with varying settings, we observed that the QM/MM optimizations for the present system did not react too sensitively to the size of the active region and that converged and stable results were obtained with the above settings.

### 2.3 Computational Details

All QM/MM calculations were performed with Orca 5.^60^ Geometry optimizations used the r^2^SCAN^61^ functional combined with Def2-TZVP^62^ basis sets on Fe, N, O, C, and H. This functional was shown to perform well for the geometry of both transition metal and main group chemistry, for example, Benediktsson and Björnsson used it for the iron–molybdenum cofactor (FeMoco) of nitrogenase, achieving accurate bond distances and accurate description of metal–ligand covalency.^63^ The RI approximation was used to speed up Coulomb integral calculation with def2/J basis sets.^64^ For geometry optimizations, the “TightOpt” settings were used with the default integration grids. Dispersion effects were included using the D4 corrections.^65^ A combination of KDIIS and SOSCF was used for facilitating self-consistent-field convergence. We also performed a three-layered ONIOM (QM/QM2/MM) for computing the spin state energetics of the APX-II model system with the high-level DLPNO-CCSD(T) method for the heme, His163, HOH264, and HOH289 (core QM layer); a medium-level layer (QM2, PBEh-3c)^66^ for Arg38, Trp41, His42, HOH300, HOH366, HOH377, HOH435, HOH454, and HOH512; and finally the rest of the system (MM layer). After optimization, vibrational frequencies were calculated numerically using the same level of theory. The derived Hessians of the QM-only regions were employed for simulating the NRVS spectra. Spectroscopic properties were computed with specific theoretical protocols that were identified as reliable in past studies.^67−73^ For each type of spectroscopy, a distinct functional and, when appropriate, a distinct basis set were used according to literature precedents. Mössbauer parameters were computed using the B3LYP^55^ functional with core-property CP(PPP)^74^ basis sets for Fe following previously defined protocols for determining isomer shifts and quadruple splittings.^74−79^ The core-property basis set has increased flexibility in the *s* functions and is essential here to ensure converged description of the density at the nucleus.^74^ A tight grid and integration accuracy (SpecialGridIntAcc 7) were used for Fe. The LC-BLYP functional^80^ was used to calculate UV–vis spectra using the time-dependent (TD-DFT) approach. LC-BLYP is a long-range corrected functional with variable admixture of exact (Hartree–Fock) exchange that reaches 100% at long range; such functionals are essential for avoiding artificial charge-transfer states in TD-DFT and have been shown to perform well for macrocyclic systems.^81−83^ X-ray absorption (XAS) and emission (XES) spectra were calculated with TD-DFT using the B3LYP functional because extensive prior experience has shown global hybrid functionals to provide good correlations with experiments for X-ray spectroscopy.^67−69,84,85^ For plotting the corresponding spectra, we used the *orca_mapspc* utility and applied a broadening of 2.0 eV. We also applied an energy shift of 150.85 eV, which was determined as per the established literature procedure by the difference between calculated XAS spectra and experimental HERFD XAS pre-edge energies for APX-II.^68^ TD-DFT calculations used the Def2-TZVP basis sets and corresponding def2/J auxiliary basis sets with the RI-J and COSX approximations.^86,87^

## 3 Results and Discussion

### 3.1 Models for APX-II

More than 100 distinct QM/MM models were created and optimized for Fe(IV)-oxo and hydroxo possibilities as well as for a number of Fe(III) models that were considered both for comparison and because they have been discussed in the literature in connection to APX-II. The diverse models were created by considering variations in the following factors:

a) Presence or not of a proximal water that hydrogen-bonds to the axial heme group and to the Arg38 side chain. The position of this water correlates with the orientation of the Arg38 side chain, which had not been resolved in the XFEL crystallographic model.^18^
b) Protonation state of His42: This can be singly or doubly protonated.
c) Different conformations of the Arg38 side chain, which result in different NH or NH_2_ groups of the guanidinium being involved in hydrogen bonding
d) Different hydrogen-bonding arrangements around the axial oxo or hydroxo group and different orientations (rotamers) of the OH group
e) Proton redistribution between Arg38 and His42
f) Proton relocation between His42 nitrogens
g) Various positions of second-sphere water molecules surrounding the active site
h) Different spin states of the iron center

**Figure 4:**
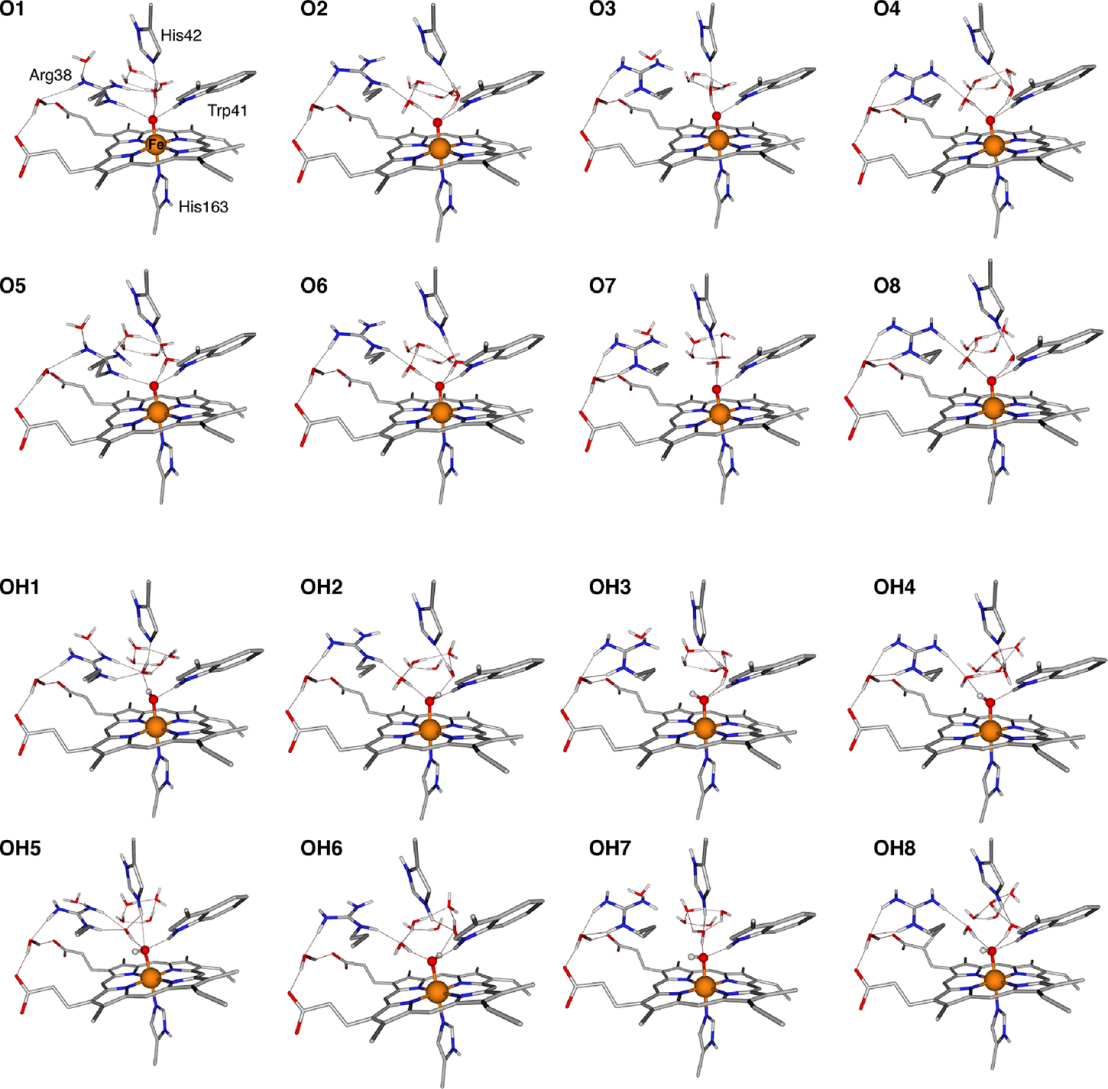
Overview of the selected model systems for Fe(IV)-oxo and Fe(IV)-hydroxo representations of APX-II. For the sake of clarity, only the QM regions are shown. Complete optimized coordinates of the depicted models are provided as Supporting Information.

To facilitate presentation and discussion, we analyzed the structural and conformational differences among all identified minima and made a selection of representative Fe(IV)=O and Fe(IV)–OH models. To establish clear structure–spectroscopy correlations in this work, we avoided a strict initial screening of models based on their computed relative energies. This was done both because computed energetics either cannot be used for nonisomeric structures or cannot be considered always reliable for complex systems with serious geometric and electronic uncertainties (which is why quantum chemical calculations of spectroscopic parameters may take precedence in such cases)^88^ and because we wanted to explicitly retain all types of model previously considered in the literature^7,17,18,29,89,90^ to ensure complete coverage of all published ideas. Moreover, by explicitly considering multiple protonation alternatives and then directly comparing them with experimental observables, we circumvent the problem of having to accurately compute p*K*_a_ values for the various groups as a precondition for constructing targeted models because we account for all possibilities without making assumptions regarding the ability of DFT to estimate such values within a highly connected hydrogen-bonded network. According to the above considerations, we chose a total of eight unique models for Fe(IV)-oxo and eight corresponding models for Fe(IV)-hydroxo. Additional variants of these APX-II models (labeled **O9-O37** and **OH9-OH37**) are reported in the Supporting Information (Tables S20 and S21, Figures S23 and S24). Figure 4 depicts the final group of selected models in their optimized ground-state geometries: the eight Fe(IV)=O models **O1**–**O8** and the eight Fe(IV)–OH models **OH1**–**OH8**. Models **O1**–**O4** and **OH1**–**OH4** have singly protonated His42, whereas models **O5**–**O8** and **OH5**–**OH8** have doubly protonated His42. Arg38 is positioned to point either “in” or “out”, with different N-bound hydrogen being involved in hydrogen bonding and with the consequent variable optimized positions of the other waters resulting in the different total numbers of hydrogen bonds involving the Fe-bound oxo or hydroxo group. The placement of Arg38 in the two different positions was based on existing structures from similar enzymes. It is relevant to note that these two orientations are not merely shifts of the side chain; i.e., they do not simply result from the side chain swinging “in” and “out”. Rather, they represent rotations of the guanidinium along the Cγ–Cδ bond and thus are not expected to be readily interconvertible. For completeness and for facilitating comparisons and discussion, we also considered three Fe(III) models, with the axial Fe ligand being OH_2_ (model **M1**, also referred to in the text as simply “ferric APX”), OH (**M2**), and O (**M3**) as shown in Figure S2. These selected models will be used for calculations of spectroscopic properties and further analysis.

The Fe(IV) center within APX-II can, in principle, adopt electronic configurations corresponding to spin states with values of total *S* = 2, 1, and 0. All of these states were considered and explicitly optimized for all structural models in this work. Consistent with experimental and theoretical data,^18,29,31,39,91^ the computed energetics confirm that all Fe(IV)-oxo/hydroxo models have an intermediate spin (*S* = 1) ground state, well-separated from either the low or the high spin alternatives (Tables S2 and S3). The ground state prediction is uniformly consistent between DFT and high-level DLPNO-CCSD(T) calculations,^92−95^ with the two approaches differing in the relative ordering of the excited *S* = 2 and *S* = 0 states, a topic that is however of no concern here. The calculated spin density plots for all 16 model ground states are shown in Figure S3, and the spin populations are listed in Table S4. The spin populations at the iron center for the ground state of models **O1**–**O8** and **OH1**–**OH8** were computed in the range of 1.47–1.67 and 1.32–1.60 electrons, respectively, consistent with the intermediate spin state of the Fe(IV) center. Significant spin population on the ferryl oxygen (0.35–0.60) in models **O1**–**O8** was also computed, suggesting some oxyl radical character. However, a very small amount of spin density was found on the oxygen atom in **OH1**–**OH8** (0.01–0.12), suggesting the almost pure OH^–^ nature of the ligand.^29^ The remaining spin density is distributed on the porphyrin ring and the proximal His163 group.

Table 1 lists the Fe–O distances of all models depicted in Figure 4. Further selected distances are provided in Table S5. It is immediately obvious that the protonation state is the principal determining factor of the Fe–O bond length. The crucial bond lengths in Fe(IV)-oxo models are considerably shorter than those of all of the Fe(IV)-hydroxo models. In addition, the Fe–O bond in the **OH1**–**OH4** models is shorter than in the **OH5**–**OH8** models. Upon closer inspection, it becomes clear that there are three distinct “classes” of Fe–O bond lengths represented by the optimized geometries:

a) a short region of ca. 1.66–1.71 Å covered exclusively by the oxo models, within which differentiations arise due to the total number of hydrogen bonds to the terminal oxo group and are also related to the protonation state of His42, with the doubly protonated forms exhibiting longer Fe–O bond lengths;
b) an intermediate region of 1.80–1.83 Å, represented by models **OH1**–**OH4**, with the shortest (OH1) being again correlated to the minimal hydrogen bonding, and
c) a long region at 1.90–1.93 Å with models **OH5**–**OH8** (doubly protonated His42).

**Table 1:** Fe–O Bond Lengths for the APX-II Models Shown in Figure4

Fe(IV)-oxo models	Fe–O (Å)	Fe(IV)–OH models	Fe–O (Å)
**O1**	1.671	**OH1**	1.798
**O2**	1.674	**OH2**	1.824
**O3**	1.659	**OH3**	1.829
**O4**	1.669	**OH4**	1.811
**O5**	1.714	**OH5**	1.932
**O6**	1.711	**OH6**	1.906
**O7**	1.678	**OH7**	1.898
**O8**	1.699	**OH8**	1.915

**Scheme 1:**
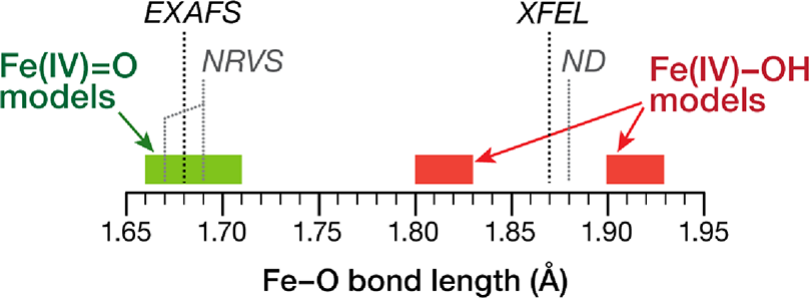
Graphical Qualitative Summary of Observations Regarding the Distribution of Fe–O Distances in Three Classes for the Models Considered in the Present Work The distances deduced from EXAFS^31^ and NRVS experiments,^38^ as well as from XFEL crystallography^17^ and neutron diffraction (ND),^18^ are also indicated for reference.

These observations are graphically summarized in Scheme 1.

Important conclusions regarding the correlation to the experiment can be reached already. First, Fe–O bond distances calculated for the Fe(IV)-oxo **O1**–**O8** models are close to and either coincide with or bracket the EXAFS derived value of 1.68 Å.^31^ The distances deduced from NRVS are also within this range.^38^ No oxo model can possibly reach the length of 1.87–1.88 Å inferred from X-ray and neutron diffraction studies.^17,18^ However, it is important to note that none of the Fe(IV)-hydroxo models reproduce these values either. The **OH1**–**OH4** models are all shorter (class b above) and the **OH5**–**OH8** are all longer (class c above) than the 1.87–1.88 Å proposed by the structural studies.^17,18^

It is crucial to emphasize what is already obvious from Scheme 1; namely, that the three ranges described above are nonoverlapping. Regardless of structural and chemical variations in the second coordination sphere (Arg38, Trp41, His42, and water molecules) and regardless of the total number of hydrogen-bonding interactions established with the axial group, the Fe–O bond lengths in the hydroxo models and in the oxo models are well separated. Therefore, the two formulations appear fundamentally distinct and can be fully differentiated based on bond length alone.

The fact that the 1.87 to 1.88 Å distance is not reproduced by the Fe(IV)-hydroxo models is perplexing. As mentioned above, we also optimized three Fe(III) models with OH_2_ (**M1**), OH (**M2**), and O (**M3**). The optimized geometries and spin density plots calculated for the ground state are shown in Figure S2. The comparison of spin populations on the iron center and the oxygen atom for Fe(IV)–OH and Fe(III)–OH revealed values of ∼1.60 versus 1.00 and ∼0.12 versus 0.05, respectively, clearly indicating distinct electronic structures between the two species. The optimized ground state Fe–O bond lengths of the three Fe(III) models are 2.119 1.869, and 1.837 Å, respectively (Tables S6 and S7). Interestingly, the optimized Fe–O bond length for the distance of the Fe(III)–OH model (**M2**) matches very well the X-ray/neutron diffraction Fe–O distances of 1.87–1.88 Å, whereas the optimized distance for the Fe(III)-oxo model (**M3**) happens to match a shorter distance of 1.84 Å reported in an early crystallographic study of APX-II.^89^ Both of these Fe(III) models fall in the intermediate region that is not populated by the Fe(IV)-hydroxo models. The present computational results do not necessarily imply that the APX-II sample in the structural studies might have been reduced to Fe(III). Nevertheless, the agreement of the crystallographic parameters with those computed here for Fe(III) models is high enough to warrant further investigation.

Given that the Fe–O bond length is of central significance for the present work, we need to address the question of the reliability of the optimized structures. Different types of dispersion corrections (D3BJ^96^ vs D4) were first considered, but these do not affect the optimized Fe–O bond lengths (Tables S8 and S5). The effect of the density functional on the optimized Fe–O bond lengths on all 16 models was tested using the alternative hybrid B3LYP functional (Table S9 and S10). The results show that the Fe–O bond length is marginally reduced by ca. 0.01 Å for the Fe(IV)-oxo of the **O1**–**O8** models and by ca. 0.02 Å for the Fe(IV)-hydroxo unit of the **OH1**–**OH4** models compared to the r^2^SCAN functional, respectively. However, the Fe–O bond length for Fe(IV)-hydroxo of the **OH5**–**OH8** models increases slightly by ca. 0.02 Å compared to that of the r^2^SCAN functional. The reason is that with B3LYP, models **OH3** and **OH5**–**OH8** converged to a different electronic structure than with the r^2^SCAN functional. Specifically, the intermediate spin state of Fe(IV)-hydroxo in these models was converted to low-spin Fe(III)-hydroxo with a cation radical on the porphyrin ring (see Figure S4 for spin density plots and Table S11 for spin populations). In itself, this computational result is significant because it shows that the assumptions behind the structural definition and protonation state of these models favor—or are more consistent with—a more reduced Fe oxidation state than the Fe(IV) state corresponding to APX-II if a standard percentage of exact (Hartree–Fock) exchange is included in the functional.

**Figure 5:**
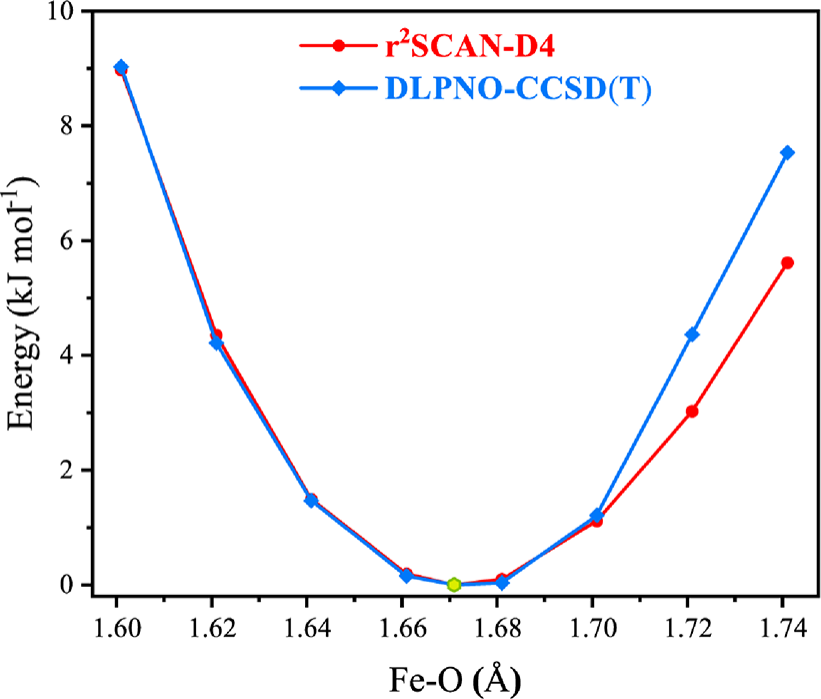
Relaxed scan of the potential energy surface with changes of the Fe–O bond with a 0.02 Å interval for Fe(IV)-oxo model **O1**, comparing the standard DFT approach used for the QM/MM optimizations with the results of DLPNO-driven coupled cluster theory.

Selected results were evaluated using a higher-level correlated wave function method, specifically the domain-based local pair natural orbital implementation of the coupled cluster method with single, double, and perturbative triple excitations, DLPNO-CCSD(T). First, we confirmed using selected models that DFT and DLPNO-CCSD(T) produce the same results regarding the ground spin state (Table S2).^18,29,31,91^ In terms of structural parameters, we conducted relaxed potential energy surface (PES) scans for representative models, scanning the Fe–O bond using the r^2^SCAN functional and then applying single-point DLPNO-CCSD(T) energy calculations along these scans. The energetics obtained with the coupled-cluster level of theory practically coincide with the DFT PES close to the minimum (Figure 5 compares the two curves for the case of Fe(IV)=O), with only a very subtle change in energy (0.1 kJ mol^–1^) with respect to the ground state bond length (1.671 Å) from 1.681 Å based on the relaxed PES scan for the **O1** model. Similarly, in the case of the Fe(IV)–OH model **OH1**, DLPNO-CCSD(T) PES scans lead to exactly the same ground state bond lengths (1.798 Å) as DFT (Table S12). These results show that the present QM/MM DFT optimizations yield accurate and reliable Fe–O parameters, thereby confirming that the two protonation state formulations do indeed differ significantly and that they do not overlap under any of the considered assumptions regarding hydrogen bonding interactions and second-sphere perturbations.

We conclude this section with some finer details about the structural models and specific energetic aspects. In the XFEL crystal structure of compound II of APX, the arginine (Arg38) side chain was not resolved, and the exact orientation of this residue is not clearly defined.^18^ Changes in the orientation of Arg38 can introduce significant conformational changes in the active site structure, as discussed by Kwon et al.;^18^ therefore, we extensively explored the conformational space with different orientations and rotations of the Arg38 side chain, correlating them with redistribution of one or more water molecules. The two major orientations are the “out” position and the “in” position (see Figure S5),^18^ the former being more frequently stabilized in our models. In terms of computed energetics, the “out” orientation of Arg38 was found to be more thermodynamically stable (11.5–62.1 kJ mol^–1^ depending on the specific model, see Table S13) than the “in” orientation. Although it is not possible to decompose the computed differences into specific contributing factors, the difference between the two orientations presumably results from a balance between the following: intra-QM and QM–MM hydrogen bonding interactions, the intrinsic energetics of Arg side chain rotation along the Cγ–Cδ bond, and the stabilization of waters either in the interior or the exterior of the cavity, which again involves both intra-QM and QM–MM interactions in our model setup. This energetic preference is further elaborated numerically if one considers additional rotational possibilities within the side chain of the residue (see Figure S6), as well as the attendant reorganization of waters and of the vicinal hydrogen-bonding network. In view of the flexibility displayed by Arg38 and the hydrogen bonding network, we expect that further elaboration and a more detailed understanding of the system can be achieved through molecular dynamics simulations that will better sample the configurational space and additionally provide a basis for mechanistic investigations. We also note that to fully consider the protonation states of residues near the active site, we considered the proton migration from Arg38 to His42 as well as within His42. Moving the proton from HH12 of Arg38 to Nε2 of His42 results in longer Fe–O bonds than in the original structure in the case of the tested **O1**, **OH1**, and **M1** models; however, these models have higher energies by more than 70 kJ mol^–1^ (Table S14). The computed energies establish a preference for the guanidinium state of Arg38 in APX-II.^97^ Similarly, moving the proton from Nδ1 to Nε2 in His42 leads to longer Fe–O bonds but also with significant energetic penalty compared to the original structures in the **O1**–**O4** and **OH1**–**OH4** models, which indicates that His42 has a strongly preferred proton position on Nδ1 compared to Nε2 (Tables S15 and S16). Overall, the “out” position of Arg38 in the present computational models is preferred energetically to the “in” position, Arg38 has preferably a guanidinium side chain, and His42 is singly protonated. All of these aspects are indeed captured by model **O4**, which we will be using as a representative model in further calculations presented in the main text (results for all models are presented in the SI), but we emphasize that **O4** is not suggested to be the unique “correct” depiction of the APX-II active site; variants with the same basic features but different hydrogen-bonding arrangements are possible and may even be preferable according to some spectroscopic observations.

The overall result of this structural analysis specifically for the Fe–O unit is that Fe(VI)-oxo models provide a very good fit to the EXAFS and NRVS derived^31,38^ APX-II Fe–O distance of ca. 1.68 Å compared to all Fe(IV)-hydroxo models. The latter yield much longer bond lengths that cover two distinct distance ranges, an intermediate and a long one, both of which bracket the crystallographically supported Fe–O distance of 1.87–1.88 Å,^17,18^ but none of which reproduces it (although an Fe(III)–OH model does). None of the three distinct distance ranges computed for the present models overlap with any other, and there is no indication of any factor, electronic or structural, that could blur the distinction between the distinct Fe(IV)-oxo/hydroxo formulations.

### 3.2 Mössbauer Spectroscopy

In this and the following sections, we shift our focus to the spectroscopic properties of the various models and discuss how they relate to the reported experimental data. We begin with Mössbauer spectroscopy, an invaluable tool for probing the oxidation state and local bonding environment of the iron atom within the heme cofactor.^98,99^ The isomer shift (δ) offers valuable information regarding the oxidation state, whereas the quadrupole splitting (Δ*E*_Q_) can be employed to monitor the protonation state of the ferryl oxygen.^25,100^ In terms of calculations, the Mössbauer isomer shift is correlated with the charge density at the nucleus, and the quadrupole splitting is estimated from the electric field gradient on the iron nucleus.^74^ We extracted the quadrupole splitting (Δ*E*_Q_) directly from the calculations; to calculate the isomer shift, we use the equation δ = α(ρ_0_ – *C*) + β, where ρ_0_ is the computed contact density at the Fe nucleus, *C* is a constant that corrects for large density values, and α and β are “correlation” constants determined by linear regression between computed ρ_0_ values and experimental isomer shifts for a given combination of theoretical methodologies using a reference set of experimentally characterized iron compounds. The computed electron density depends on the electronic structure methodology employed; therefore, different sets of α and β constants have been derived in the literature for different functional and basis set combinations. Here we used the B3LYP functional with the CP(PPP) basis set for Fe, in which case the values of α, *C*, and β were determined as −0.366, 11,810, and 2.852, respectively.^75^ Past methodological studies suggest that isomer shift values are reliably predicted by DFT, with possibly reduced reliability for quadrupole splittings.^76−78^

Table 2 provides a comprehensive summary of the computed and experimental Mössbauer parameters for APX-II (see also Table S17). The isomer shifts calculated for all models are found to be similar in magnitude with the exceptions of the **OH3** and **OH5**–**OH8** models. The reason for the deviation of the latter is that with the hybrid functional, these models converted into different electronic structures with Fe(III)–OH and a porphyrin-based radical. Most models are found to be within or close to the experimental data, and the difference between the two isomer shifts is Δ_max_δ = 0.02. This reflects the fact that the isomer shift is a very local property that is principally determined by the oxidation state of the iron center and is less sensitive to the protonation state of the ligand.

**Table 2:** Calculated Mössbauer Parameters for All Fe(IV)-oxo/hydroxo Models Compared to Experimental Mössbauer Data for APX-II

model	δ	Δ*E*_Q_	model	δ	Δ*E*_Q_
**O1**	0.06	1.70	**OH1**	0.06	2.72
**O2**	0.06	1.72	**OH2**	0.08	2.81
**O3**	0.07	1.48	**OH3**^a^	0.27	–2.71
**O4**	0.07	1.64	**OH4**	0.07	2.82
**O5**	0.05	2.11	**OH5**^a^	0.30	–2.59
**O6**	0.05	2.05	**OH6**^a^	0.28	–2.54
**O7**	0.06	1.72	**OH7**^a^	0.28	–2.73
**O8**	0.06	1.95	**OH8**^a^	0.29	–2.53
**exp**	0.05	1.66	**exp**	0.05	1.66

a For these hydroxo models, the initial (Por)Fe(IV)–OH switched to (Por^+•^)Fe(III)–OH upon using the B3LYP functional for the Mössbauer calculations.

Quadrupole splitting values are much more sensitive to changes in the anisotropy of the environment around the Fe nucleus and hence exhibit greater fluctuations. The calculated Δ*E*_Q_ values for Fe(IV)-oxo models are in much better agreement with the experimental data,^38^ whereas all Fe(IV)-hydroxo models exhibit considerably higher quadrupole splittings that render them incompatible with experiment. Therefore, Δ*Ε*_Q_ is a sensitive and decisive reporter of the protonation state of the axial ligand to Fe. We note that in the context of ferryl protonation within P450-II, an increase in Δ*E*_Q_ is observed, shifting from 1.30 to 2.02 mm/s when the ferryl oxygen is protonated, and the thiolate-ligated chloroperoxidase compound II was found to have similar results.^100^ Because similar results are observed when the ferryl oxygen is protonated, Δ*E*_Q_ increases by approximately 1.10 mm/s from the Fe(IV)-oxo model to the Fe(IV)-hydroxo model in APX-II. For completeness, we also calculated the Mössbauer parameters for the originally considered **M1**, **M2**, and **M3** models. The calculated isomer shifts for these models are δ = 0.30, 0.29, and 0.39 mm/s, respectively, and the quadrupole splittings are Δ*E*_Q_ = −2.89, −3.01, and −0.81 mm/s. All of these results are in disagreement with the experimental Mössbauer data on APX-II, and therefore, Fe(III) models are conclusively excluded from consideration.

The experimental Mössbauer data of APX-II show very similar isomer shift and quadrupole splitting as in other enzymes with a ferryl iron with spin *S* = 1 for compound II.^27,31,35,101^ X-ray absorption and resonance Raman measurements revealed that HRP-II, CCP-II, and myoglobin (Mb)-II contain authentic iron(IV)-oxo species.^34,35,102^ The resemblance in Mössbauer parameters between APX-II and these systems implies that APX-II also represents an iron(IV)-oxo species at a pH of 7. Among oxo models, **O1**, **O2**, **O4**, and **O7** yield calculated Mössbauer parameters that align very well with the experiment and closely resemble the parameters reported for APX-II.^31,38^ Given that the nonhybrid TPSS functional^75^ has also been calibrated for the prediction of Mössbauer parameters, we also used this one on the complete series of models to “compensate” for the fact that B3LYP converts several of the hydroxo models to the more stable Fe(III) form. The TPSS results (Tables S18 and S19) confirm the conclusions already reached above; namely, that regardless of the choice of method and the precise electronic structure, all hydroxo models are incompatible with the experiment.

### 3.3 UV–Vis Spectroscopy

**Figure 6:**
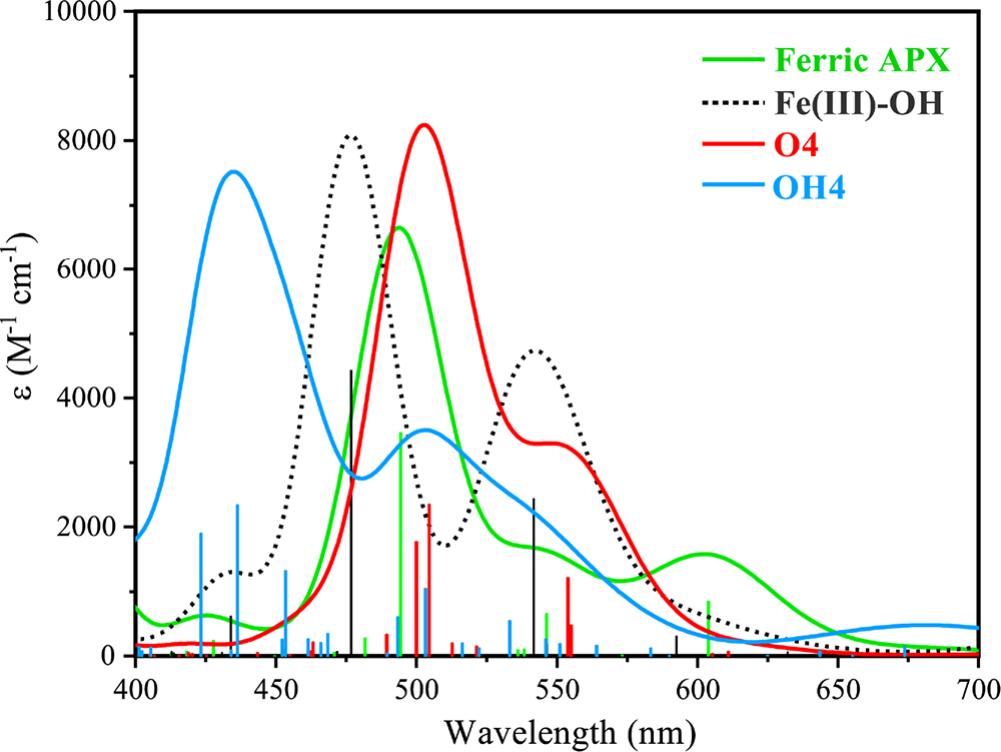
Absorption spectra (Q-band region) computed using TD-DFT calculations on the ground state structures of ferric APX (model **M1**), Fe(III)–OH (model **M2**), Fe(IV)-oxo model **O4**, and Fe(IV)-hydroxo model **OH4**.

Stopped-flow UV–vis measurements have been used to identify the generation of compound I of APX (APX-I, an iron(IV) oxo porphyrin radical species, λ_max_ = 409, 527, 575^sh^, 649 nm) on the reaction of ferric APX with the two-electron oxidant *m*-chloroperbenzoic acid. The formed APX-I species rapidly decays to generate the ferryl compound II intermediate (λ_max_ = 415, 528, and 559 nm), which is stable over long (8.3 min) time scales.^17,18,89^ Green and co-workers used a mixture of ferric APX with 5 equiv of *m*-CPBA to increase the aging time to several seconds to allow for maximum APX-II formation. In ferric APX, the Soret maximum is at 405 nm, with Q bands at 509 and 544 nm, whereas in APX-II, it is at 418 nm, with lower energy bands at 528 and 559 nm.^31^ Thus, APX-II formation is revealed by a shift of all features to lower energy compared to ferric APX. Green and co-workers proposed an authentic Fe(IV)-oxo species,^31^ whereas Moody and co-workers proposed an authentic Fe(IV)-hydroxo species^17,18,89^ in APX-II, as described by apparently similar UV–vis spectra. Despite this fundamentally different interpretation regarding the protonation state of APX-II, the definitive experimental observation is the redshift accompanying the formation of APX-II. As discussed below, this does serve as a discriminating criterion between the competing formulations when we compute explicitly the absorption spectra of the different models from first principles. The TD-DFT approach with a long-range corrected functional was used to compute the excited state energies for all Fe(IV)-oxo and Fe(IV)-hydroxo models. Figures S7 and S8 report the complete series of simulated spectra, whereas here in the main text, Figure 6 compares the calculated UV–vis spectra of ferric APX, Fe(III)–OH, Fe(IV)=O (**O4**), and Fe(IV)–OH (**OH4**). The corresponding “stick spectra” with individual intensities are shown in Figure S9. Explicit decomposition of the most intense peaks in terms of the natural transition orbitals (NTOs) corresponding to all species is provided in Figure S10.

The spectra simulated on the basis of TD-DFT calculations show three major features, in agreement with the experimental observations. The Soret feature (390–430 nm, Figure S11) is blue-shifted in the calculated spectra, as observed also by Ramos et al.,^103^ presumably because of limitations of TD-DFT in describing the types of excitation dominating the B-band,^81,104,105^ but the Q-band is known to be reliably computed.^81^ Calculations of ferric APX give peaks at 496 and 546 nm, matching well with the experiment.^31^ The peak at 496 nm (S_14_) has a porphyrin (π*) to iron (d_*xz*/*yz*_) charge transfer character, and the peak at 546 nm (S_10_) is the expected Q-type porphyrin-based π*−π* excitation. The other peak at 604 nm (S_8_), which also has a Q-type porphyrin π*−π* character, is found to be slightly blue-shifted compared with the experiment. Calculations predict similar peaks at 477 (S_13_) and 542 nm (S_11_) for our Fe(III)–OH model. The nature of these features is similar to that of ferric APX. For the Fe(IV)-oxo model, our simulations yield peaks at 503 and 550 nm. The peak at 503 nm consists of the two states (S_12_ and S_13_), both with a similar porphyrin (π*) to iron (d_*xz*/*yz*_) character. The peak at 550 nm consists of principally two states (S_7_ and S_8_), one with a mixed porphyrin-based as well as π* to iron (d_*xz*/*yz*_) character and the other with local porphyrin-based excitation. These are in good agreement with the experiment and reproduce the red-shift observed experimentally for APX-II compared to ferric APX. The calculations for the Fe(IV)-hydroxo model yield a similar spectral shape, but the crucial peaks are blue-shifted compared to ferric APX (434, 503, 533^sh^ nm). The peak at 434 nm consists of the three states that have a similar character, resulting from mixed porphyrin and axial histidine (π*) to iron (d_*xz*/*yz*_) transitions. The peak at 503 nm (S_21_) has an important ligand-to-metal charge transfer character, and a similar feature is also observed in the shoulder peak at 533 nm (S_18_). This shows that protonation results in the shifting of the major features to shorter wavelengths compared to the oxo species, presumably as a result of the different mixing of axial histidine character and the adjustment of the Fe orbital energies. This could be related to the emergence of a shoulder peak at 533 nm in the hydroxo case and the absence of a peak in the region close to 560 nm as required for agreement with the experimental UV–vis spectrum of APX-II. The same conclusions regarding the suitability of Fe(IV)-oxo vs Fe(IV)-hydroxo as a model for the experimental spectra are reached if we consider the additional results for the remaining models reported in the SI. Overall, the calculations reported here suggest that the best interpretation of the experimental observations is in line with an Fe(IV)=O species, whereas Fe(IV)-hydroxo models show the opposite trend than what is experimentally observed for the UV–vis spectrum of APX-II.

### 3.4 X-ray Absorption Spectroscopy

X-ray absorption spectroscopy (XAS) is a powerful and element-specific technique for the structural investigation of inorganic complexes and active sites of metalloenzymes.^67,69,73,106−108^ An XAS “edge” results from a core electron absorbing energy that matches or exceeds its binding energy. For the Fe K-edge, this corresponds to ∼7.1 keV, the binding energy of an Fe 1s electron, and the primary transition is the dipole-allowed 1s → 4p excitation. Shifts observed in the Fe K-edge energy offer a measure of changes in the effective nuclear charge at the absorbing atom and thus can be correlated to the oxidation state. Beyond the rising edge, the extended X-ray absorption fine structure (EXAFS) provides structural information, accurate for the first-shell Fe–ligand distance, less so for the overall coordination sphere, and generally limited for the overall active site geometry. At energies lower than the rising edge, weak features can be observed in the K-pre-edge region. A pre-edge feature observed in an XAS spectrum is formally attributed to a 1s → 3d quadrupole-allowed transition. This is much weaker compared to the dipole-allowed 1s → 4p main edge transition. However, distortions from centrosymmetry enable the mixing of 4p with 3d orbitals, giving this transition an electric dipole-allowed feature and thus increasing the pre-edge intensity. The Fe pre-edge feature thus provides information on the oxidation state, geometry, coordination, site symmetry, and, in some cases, the spin state of the iron atom.^109−111^

**Figure 7:**
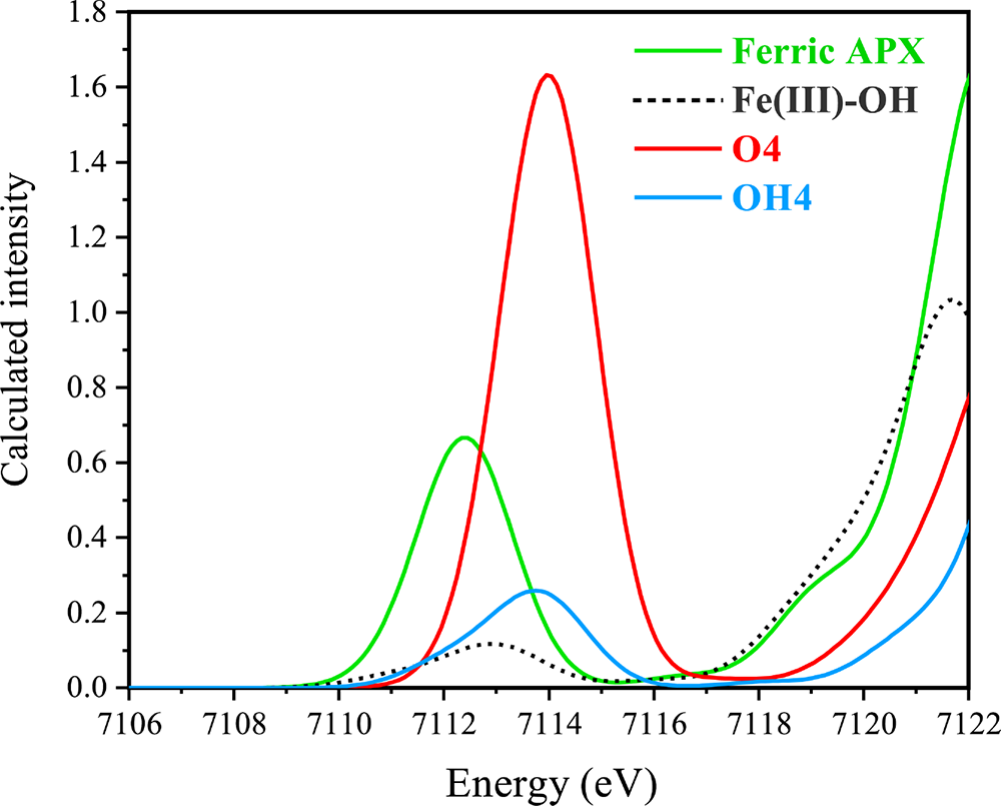
Calculated TD-DFT Fe K pre-edge XAS for **ferric APX**, **Fe(III)–OH**, **O4** and **OH4** of APX-II.

The Fe K-edge XAS pre-edge analysis has been particularly useful for studying APX-II.^24,25,35,112^ The pre-edge energy generally increases with the strength of the ligand field around the Fe atom and is additionally modulated by the metal oxidation state.^67,73^ As a result, under the same ligand environment, the Fe(IV) oxo species should exhibit higher pre-edge energy than the corresponding Fe(III/IV)-hydroxo species. The previously reported results for cytochrome P450 of iron(IV)-oxo and iron(IV)-hydroxide species showed that an unprotonated form of P450-II features a sharp pre-edge that is significantly weakened upon protonation of the ferryl moiety.^25^ Similar observations were made for iron(IV)-oxo compared to iron(III)-hydroxide forms of Mb.^35^ The experimental XAS data reported by Ledray et al. show that the pre-edge of unprotonated Mb-II and P450-II resembles closely that of APX-II, contrasted to their protonated forms, implying the same unprotonated nature of the Fe(IV) species in APX-II as in the Fe(IV)-oxo forms of P450-II and Mb-II.^31^ Using the observations of Ledray et al. as a guide, here we compare the computed pre-edge region for the ferric APX, Fe(III/IV)-hydroxo, and Fe(IV)-oxo model systems of APX-II using an established and calibrated TD-DFT protocol.^69^ Fe K-edge pre-edge spectra of all 16 QM/MM models, including ferric APX and Fe(III)–OH species, are reported in Figures S12 and S13. Figure 7 displays the calculated Fe K-edge pre-edge of the representative ferric APX (**M1**), Fe(III)–OH (**M2**), **O4**, and **OH4** species.

The calculated pre-edge region is reproduced well by the TD-DFT calculations and is in line with the experiment.^31^ With respect to the ferric APX pre-edge maximum, the calculations yield an upshift of approximately 1.5 eV for the APX-II models, which is similar to the reported data on P450-II and Mb-II and consistent with the presence of an iron(IV).^31,67,112^ We note that Fe(III)–OH is found to be slightly above the pre-edge of ferric APX by 0.5 eV. In terms of energies, the pre-edge of Fe(IV)-oxo is found to be slightly higher (ca. 0.3 eV) than Fe(IV)-hydroxo, suggesting that an energy-based differentiation might be uncertain. However, the intensity differences are dramatic and decisive. The calculated pre-edge intensity for ferric APX is almost three times lower than that of the Fe(IV)-oxo APX-II model, consistent with the previously reported data for P450-II and Mb-II, whereas the pre-edge of the Fe(IV)-hydroxo form is considerably weaker.

It is readily attributed to the lengthening of the Fe–O bond in the case of the hydroxo model that the intensity and transition energy decrease, as shown in Figure S14. Analysis of the low energy features shows that they correspond primarily to an Fe 1s → 3d_*z*^2^_ transition. As the lengthening of the Fe–O bond reduces the mixing of 3d_*z*^2^_–4p_*z*_, the ligand field is reduced as well as the intensity of the pre-edge.^113^ The Löwdin reduced orbital population analysis of the ferric APX, Fe(III)–OH, **OH4**, and **O4** species shows that the total Fe 4p character mixed into the 3d orbitals is greatest for **O4** (1.1%), decreases slightly in ferric APX (1%), and decreases significantly in **OH4** (0.1%) and even more in Fe(III)–OH (0%). These computed values precisely mirror the trends in the intensities observed in Figure 7. In conclusion, the calculations are fully consistent with the conclusions of Ledray et al.^31^ that the experimental XAS spectra of APX-II can be attributed uniquely to an authentic Fe(IV)-oxo species rather than an Fe(IV)-hydroxo species.

### 3.5 X-ray Emission Spectroscopy

X-ray emission spectroscopy serves as a complementary tool to probe metal sites and investigate the protonation state of enzymatic systems.^24,114,115^ The Fe Kβ XES provides insight into the iron active site structure, including the spin state, oxidation state, and ligand identity.^116−118^ In Kβ XES, photons are emitted via electron decay after the 1s electron of a metal is ionized. The spectrum encompasses the Kβ_1,3_ emission (electric dipole-allowed 3p → 1s transition)^119^ and higher-energy valence-to-core transitions identified as ligand *n*p to metal 1s transitions (Kβ_2,5_) and ligand *n*s to metal 1s transitions (Kβ″).^120,121^ Given the established utility of Fe XES spectroscopy,^114,122−124^ it is interesting to compare our computational APX models also in this respect despite the absence so far of experimental data.

**Figure 8:**
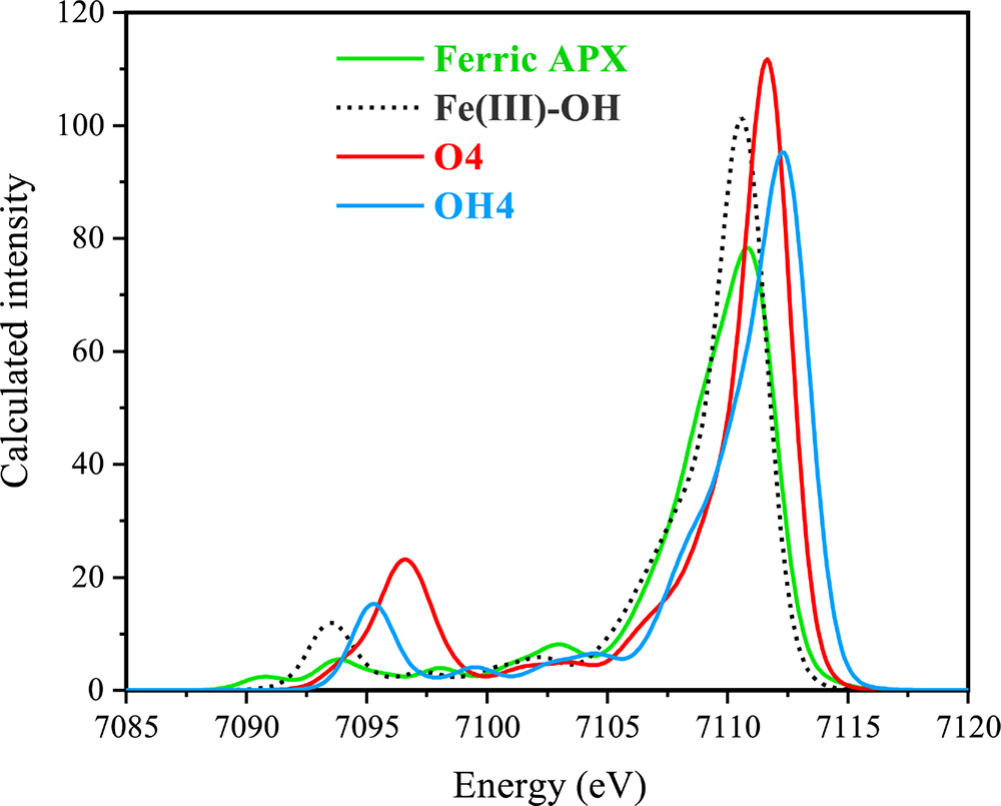
Calculated valence to core spectra for **ferric APX**, **Fe(III)–OH**, **O4**, and **OH4** of APX-II.

The calculated valence-to-core XES spectra shown in Figure 8 for ferric APX, Fe(III)–OH, **O4**, and **OH4** exhibit two major features in the valence to the core region: weak Kβ″ features at ca. 7093.7, 7093.4, 7096.6, and 7095.3 eV, respectively (although more features are present, particularly for the ferric APX), and intense Kβ_2,5_ features at 7110.8, 7110.6, 7111.6, and 7112.3 eV, respectively, for the four models (see Figures S15 and S16 for additional models; Figure S17 describes the orbitals involved in major transitions for all models). The Kβ″ peak discriminates the Fe(IV)=O and Fe(IV)–OH models in that the former has a highest energy and a somewhat higher intensity. Both are clearly higher than those of the Fe(III) models. The most intense transitions in the satellite region arise principally from O 2s contributions (Figure S17), but the models are differentiated by the fact that with the exception of O4, there are significant mixed contributions from other ligands.^70,125^ The intensity of the Kβ feature is lower in Fe(III)–OH and **OH4** compared to that in **O4**. The VtC-XES intensity is correlated to the metal–ligand bond distance: in the case of **O4**, the Fe–O distance is shorter compared to that of **OH4**, Fe(III)–OH, and ferric APX (by 0.142 0.2, and 0.448 Å, respectively), resulting in a more intense feature.

The higher energy Kβ_2,5_ feature is attributed to ligand bonding and nonbonding 2p-based orbitals. In all instances, there is a substantial Fe 4p character (ca. 1–3% Fe 4p) conferring dipole-allowed intensity. The Löwdin reduced orbital population analysis of ferric APX, Fe(III)–OH, **OH4**, and **O4** species shows that the total Fe 4p character mixed into the 3d orbitals is greatest for **O4** (3.1%), decreases slightly in Fe(III)–OH (2.7%), and decreases more in **OH4** (1.9%) and in ferric APX (1.7%). The oxo models have a substantial amount of Fe p character compared with the hydroxo models. For discussions of the relative importance of 3p versus 4p contributions to Fe XES, we refer the reader to more detailed studies.^113,126,127^ Overall, the results suggest that XES could complement XAS as a probe of perturbations at the iron site and distinguish between the two protonation possibilities.

### 3.6 Nuclear Resonance Vibrational Spectroscopy

Ledray et al.^38^ have used nuclear resonance vibrational spectroscopy (NRVS)^128,129^ to investigate the iron site in APX-II and deduce Fe–O bond lengths via Badger’s rule based on the observed vibrational features. NRVS has previously been employed in the investigation of the stretching vibrations of iron(IV)-oxo species in myoglobin compound II, as well as in synthetic compounds.^130−135^ In addition to being element-specific for Mössbauer-active nuclei, it has the advantage of diminished flux density in comparison to X-ray-based methodologies and does not involve electronic excited states, in contrast to resonance Raman spectroscopy, and the Fe–O vibrational resonance mode can be readily observed as it appears in a noncongested frequency region. Badger’s rule correlates Fe–O bond distances with Fe–O vibrational frequencies and has been applied successfully to heme and nonheme iron–oxygen bonds.^28,136^ Experimental NRVS data for APX-II show a broad feature between 700 and 780 cm^–1^, with two peaks appearing at 732 and 770 cm^–1^.^38^ According to Badger’s rule, these correspond to Fe–O distances of 1.69 and 1.67 Å, respectively, which align closely with the distance of 1.68 Å obtained by EXAFS^38^ but not with crystallography.

**Figure 9:**
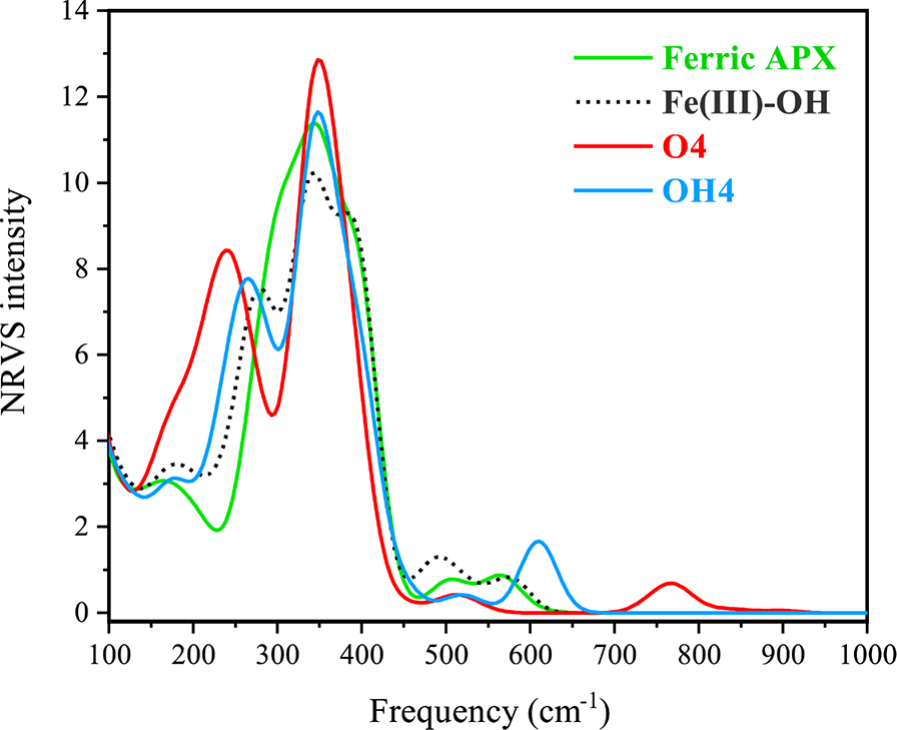
NRVS spectra of the **ferric APX**, **Fe(III)–OH**, **O4**, and **OH4** of APX-II computed with r^2^SCAN/def2-TZVP (*T* = 298.15 K).

To approach this topic computationally, we performed numerical frequency calculations as a basis for subsequently simulating the NRVS spectra. The calculated resonant Fe–O stretching vibrational modes for all Fe(IV)-oxo models are in the range of 669–794 cm^–1^, whereas those for all Fe(IV)-hydroxo models are in the range of 352–641 cm^–1^ (Table 3, Figures S18 and S19). These values show explicit correlation to the Fe–O distances of the models, with lower values correlated with longer Fe–O distances and vice versa. Given that the present results directly connect the distance to the vibrational frequency via explicit electronic structure calculations, they can be viewed as confirming the validity of Badger’s rule as used in the experimental studies. Among oxo models, the computed vibrational modes in **O1**, **O2**, **O4**, and **O7** are very close to the experimental data (closer to the 770 cm^–1^ experimental peak for the first three and close to the 732 cm^–1^ peak for the fourth one). In contrast, all Fe(IV)-hydroxo models deviate strongly from the experiment. From a direct comparison between the Fe(IV)-oxo of the **O4** model, which is in excellent agreement with the 1.67 Å Fe–O bond deduced by Badger’s rule for APX-II, the Fe(IV)-hydroxo of the **OH4** model with a bond length of 1.81 Å is shifted by 166 cm^–1^ (Figure 9).

**Table 3:** Fe–O Vibrational Modes (cm^–1^) Based on the Computed NRVS Spectrum for All APX-II Models Considered in This Work

Fe(IV)-oxo models	ν(Fe–O)	Fe(IV)–OH models	ν(Fe–O)
**O1**	770	**OH1**	641
**O2**	763	**OH2**	598
**O3**	794	**OH3**	587
**O4**	776	**OH4**	610
**O5**	669	**OH5**	352
**O6**	684	**OH6**	391
**O7**	739	**OH7**	484
**O8**	708	**OH8**	501

We note that the computed values for the hydroxo models (particularly for model **OH3**) are consistent with previously reported resonance Raman derived data for the Fe(IV)–OH stretching mode in chloroperoxidase compound II (565 cm^–1^ at 1.82 Å).^137^ For comparison, we also calculated the Fe–O vibrational resonance mode for the Fe(III)–OH model; this model, which has an Fe–O distance of 1.87 Å in agreement with XFEL crystallography, has an Fe–O mode at 571 cm^–1^. This pair of values agrees very well with observations on a different system, specifically myoglobin Fe(III)–OH, with an EXAFS-derived Fe–O distance of 1.86 Å and a stretching frequency of 556 cm^–1^, consistent with the ferric hydroxide state.^130^

In conclusion, these NRVS calculations strongly support the assignment of the experimental spectra to a genuine Fe(IV)-oxo species for APX-II^28,38^ and show that no hydroxo model (Fe(IV) or Fe(III)), under any local perturbations of the active site, can possibly provide a feature at the 730–770 cm^–1^ region observed in the NRVS spectra of APX-II.

### 3.7 Molecular Dynamics Simulations

Based on the various studied models of the heme active site in APX-II and a detailed spectroscopic analysis, we showed that all spectroscopic observations are consistent only with a genuine Fe(IV)-oxo species with a short Fe–O bond of ca. 1.7 Å. Herein, we selected two active site models for Fe(IV)-oxo (**O1** and **O4**) corresponding to the “in” and “out” configuration of the Arg38 side chain, respectively, to assess the conformational stability of the protein in each case through MD simulations. Both systems equilibrated within 400 ps of the equilibration dynamics, and the density remained stable up to 1 ns. After this point, we initiated independent MD production runs for 50 ns with selected restraints as described in the Computational Details section. Analysis of the MD trajectories indicates that the heme active site retains a stable H-bond network in both configurations. Both the “in” and the “out” conformations of Arg38 are stable and do not interchange (i.e., flip of the side chain along the Cγ–Cδ bond) within our MD time scales. The distances between the Arg38 Nε and the axial oxo for the **O1** configuration remain stable in the range of 3–4 Å throughout the MD production runs. In the case of **O4**, this H-bond is replaced by the bridging water molecule between the Arg38 side chain and the oxo ligand, which also remains stable within our MD time scales. The overall side-chain fluctuations of the protein, the time evolution of critical distances, and their frequency distributions along the MD trajectories are shown in Figures S20–S22. The protein RMSD is similar for both models but deviates more from the equilibrated protein conformation in the case of **O1** than in **O4**, which stays more compact. Also, the environment around the heme active site is found to be more dynamic in the case of **O1** (Figure S20). On the other hand, the His42 residue was seen to be more flexible in the case of the **O4** starting model, as the side chain could switch toward an H-bonding interaction with the Arg38 side chain. The solvent molecules within 5 Å of the heme moiety do not show dynamic changes, and the water network around the heme active site is conserved in both cases. The average number of water molecules within a 3 Å radius of the heme site is found to be 7.4 for **O1** and 7.7 for **O4**, which suggest that the orientation of Arg38 does not affect the immediate hydrogen bonding environment and the hydration level of the active site. More comprehensive studies of dynamics at physiological conditions, possibly coupling classical with *ab initio* MD simulations, will be required to understand the conformational flexibility of Arg38 and its potential mechanistic role in functions such as proton transfer.^138^ The present work settles a major open question about the nature of the heme cofactor in APX-II so that these future studies can more confidently focus on investigating second-sphere effects and reactivity aspects.

## 4 Summary and Conclusions

The protonation state of the ferryl compound II intermediate in ascorbate peroxidase, Fe(IV)-oxo or Fe(IV)-hydroxo, has been a subject of debate. Different studies have reached conflicting conclusions, with neutron diffraction and XFEL crystallography supporting a long Fe–O distance consistent with a protonated (hydroxo) form,^17,18^ whereas several spectroscopic techniques are consistent with the Fe(IV)=O form.^31,38^ A hypothesis advanced to accommodate this discrepancy suggests that the moiety may display high flexibility that would result in an overlap of the expected bond length ranges for the two protonation states, rendering the bond length a weak criterion for differentiation. In the present work, we approached the problem using spectroscopy-oriented computational chemistry by constructing large all-atom models of the protein and conducting extensive multiscale QM/MM calculations. We considered multiple variants of the active site based on a comprehensive series of first- and second-sphere modifications and subjected the models to a complete series of spectroscopic calculations for direct comparisons to all available experimental data.

The structural investigation provides a detailed picture of the APX-II site, describing the effects of orientation/conformation changes of the distal Arg38 residue that is involved in proton delivery from the substrate (ascorbate) to the heme group during catalysis, the protonation state of His42, and the hydrogen-bonding (re)arrangements around the ferryl group. In terms of the crucial Fe–O distances, our results show that Fe(IV)=O and Fe(IV)–OH models occupy clearly distinct Fe–O bond length ranges regardless of second-sphere modifications. The oxo species span a relatively narrow range below 1.7 Å, which encompasses the short Fe–O distances deduced for APX-II by EXAFS (1.68 Å) and by NRVS (1.67 and 1.69 Å). Hydroxo models span a wider range that is actually split into a region below ca. 1.84 Å and a region above ca. 1.90 Å. The latter is associated with a doubly protonated histidine residue (His42), a state that is strongly disfavored energetically and creates an electronic situation that favors the reduction of the Fe ion by the porphyrin. There is a ca. 0.1 Å difference between the longest oxo and the shortest hydroxo; therefore, we conclusively exclude the possibility of overlap in bond lengths: the two protonation states are distinct and distinguishable. Interestingly, no Fe(IV)–OH model can reproduce the 1.87–1.88 Å distances reported by Kwon et al.^17,18^ These fall into an intermediate region that is not populated by any of the Fe(IV) models considered in the present work. The only computed models that have such Fe–O distances are models containing an Fe(III) ion instead.

In addition to the structural analysis, we computed properties related to all types of spectroscopies reported experimentally, including Mössbauer, optical absorption, XAS, and NRVS, as well as XES that has not yet been employed in practice for this system. Comparison of calculations with the experiment supports the assignments made by Ledray et al.,^31,38^ namely, that all spectroscopic observations on APX-II are consistent with—and only with—a genuine Fe(IV)-oxo species with a short Fe–O bond.

The impossibility of reconciling spectroscopic observations with the long crystallographic Fe–O distance and also the difficulty in reproducing the latter with Fe(IV)–OH models as opposed to Fe(III)–OH analogues lead naturally to the question of whether the long distances reflect the reduction of ferryl APX-II to a ferric species despite the care taken experimentally to control and minimize radiation damage.^17^ Our computational results can hint at this possibility but not substantiate it. It is worth noting that XFEL sources are supposed to have precisely this advantage in protein crystallography over conventional synchrotron sources (“diffraction before destruction”);^139^ however, this has been challenged in some cases and should not be accepted as a general fact.^140−143^ The situation with the Fe–O bond length and protonation discrepancy for APX-II is reminiscent of similar examples in the past (such as the case of myoglobin^29,35,42,130,144^) where divergence between crystallographic and spectroscopic data was often related eventually to the photoreduction of Fe(IV).

Overall, the present work supports the Fe(IV)-oxo formulation for APX-II and provides detailed information about favorable structural and electronic aspects of the intermediate and its immediate environment, including protonation states and conformational preferences of vicinal residues. At this point, we cannot single out a specific oxo model as a unique best fit to the spectroscopic data, and we suggest that such an attempt might even be in principle incorrect: the flexibility of the hydrogen bonding network, the mobility of proximal waters, and the effects of pH can all contribute to the coexistence of more than one population, which is already hinted at by NRVS data.^38^ The present study hopefully provides motivation to revisit and extend some of the experimental work, and by establishing concrete whole-enzyme models and validated methodologies, it also lays the groundwork for extending computational studies to investigations of how the protein environment and protein dynamics tune acidity and redox potential to control reactivity.

## Supporting Information Available

Comparison of QM regions (Figure S1, Table S1); models **M1**–**M3** (Figure S2); comparison of spin states (Tables S2 and S3); spin density plots for all models (Figures S3 and S4); Mulliken spin populations (Table S4); additional optimized parameters (Table S5); results for Fe(III) models (Tables S6 and S7); Fe–O bond lengths optimized with r^2^SCAN-D3BJ (Table S8); Fe–O bond lengths optimized with B3LYP-D3BJ (Table S9); Fe–O bond lengths optimized with B3LYP-D4 (Table S10); B3LYP-D3BJ Mulliken spin populations (Table S11); relaxed scan results (Table S12); Arg38 positions (Figure S5); relative energies with respect to Arg38 orientation (Table S13); rotamers and conformers of Arg38 (Figure S6); geometric parameters of protonation alternatives (Tables S14–S16); deviations of computed Mössbauer parameters from experimental data (Table S17); TPSS Mössbauer parameters (Table S18); TPSS-D3BJ Mulliken spin populations (Table S19); UV–vis absorption spectra (Figures S7–S9); natural transition orbitals for selected transitions (Figure S10); extended UV–vis spectra of the four selected models (Figure S11); Fe K pre-edge XAS spectra and selected assignments (Figure S12–S14); XES spectra and selected assignments (Figures S15–S17); NRVS spectra (Figures S18 and S19); MD results (Figures S20–S22); additional computational details and example input files; and additional models/variants (Tables S20 and S21, Figures S23 and S24) (PDF)
Cartesian coordinates of all structural models discussed in the main text (TXT)
Example input files for QM/MM calculations (TXT)
Accompanying force field parameter set (prms) for the provided PDB file (TXT)
PDB file representing the complete system setup (PDB)

## Supplementary Material

ja3c13169_si_004.txt

ja3c13169_si_005.pdb
