# Supplementary material for: Correlating Structure with Spectroscopy in Ascorbate Peroxidase Compound II
Source: J Am Chem Soc. 2024 Mar 26;146(14):9640–56. doi: 10.1021/jacs.3c13169 (PMC11009960; doi:10.1021/jacs.3c13169)
Supplement: Supplementary file 1 — ja3c13169_si_001.pdf [file ja3c13169_si_001.pdf]

SUPPORTING INFORMATION

for

Correlating Structure with Spectroscopy in Ascorbate Peroxidase Compound II

Mursaleem Ansari, Sinjini Bhattacharjee, Dimitrios A. Pantazis*

Max-Planck-Institut für Kohlenforschung Kaiser-Wilhelm-Platz 1, 45470, Mülheim an der Ruhr, Germany

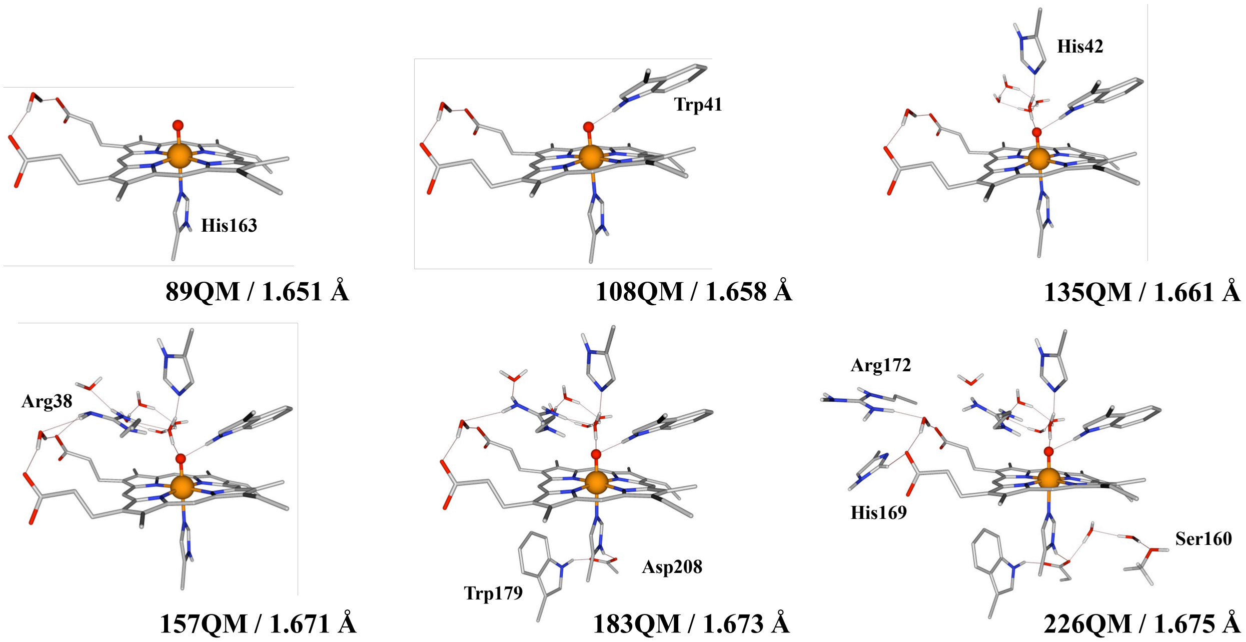

Figure S1. QM regions of varying sizes used to study the convergence of QM size of the Fe(IV)-oxo model in APX-II for the ground state. The label denotes the number of QM atoms in the corresponding QM/MM calculations, and the distance is the Fe–O bond length obtained after QM/MM optimization, with the r^{2}SCAN-D4/def2-TZVP method.

Table S1. Comparison of Fe–O bond lengths in Fe(IV)-oxo and -hydroxo models obtained for various sizes of QM region in QM/MM optimizations of their respective ground states, calculated using the r^{2}SCAN-D4/def2-TZVP level of theory. For reference, a distance of 1.68 Å was reported for APX-II by EXAFS[1] and a distance of 1.87 Å by XFEL crystallography.[2]

Fe(IV)-oxo models	Fe–O (Å)	Fe(IV)-hydroxo models	Fe–O (Å)
**89QM**	1.651	**90QM**	1.819
**108QM**	1.658	**109QM**	1.839
**135QM**	1.661	**136QM**	1.821
**157QM**	1.671	**158QM**	1.798
**183QM**	1.673	**184QM**	1.833
**226QM**	1.675	**227QM**	1.836

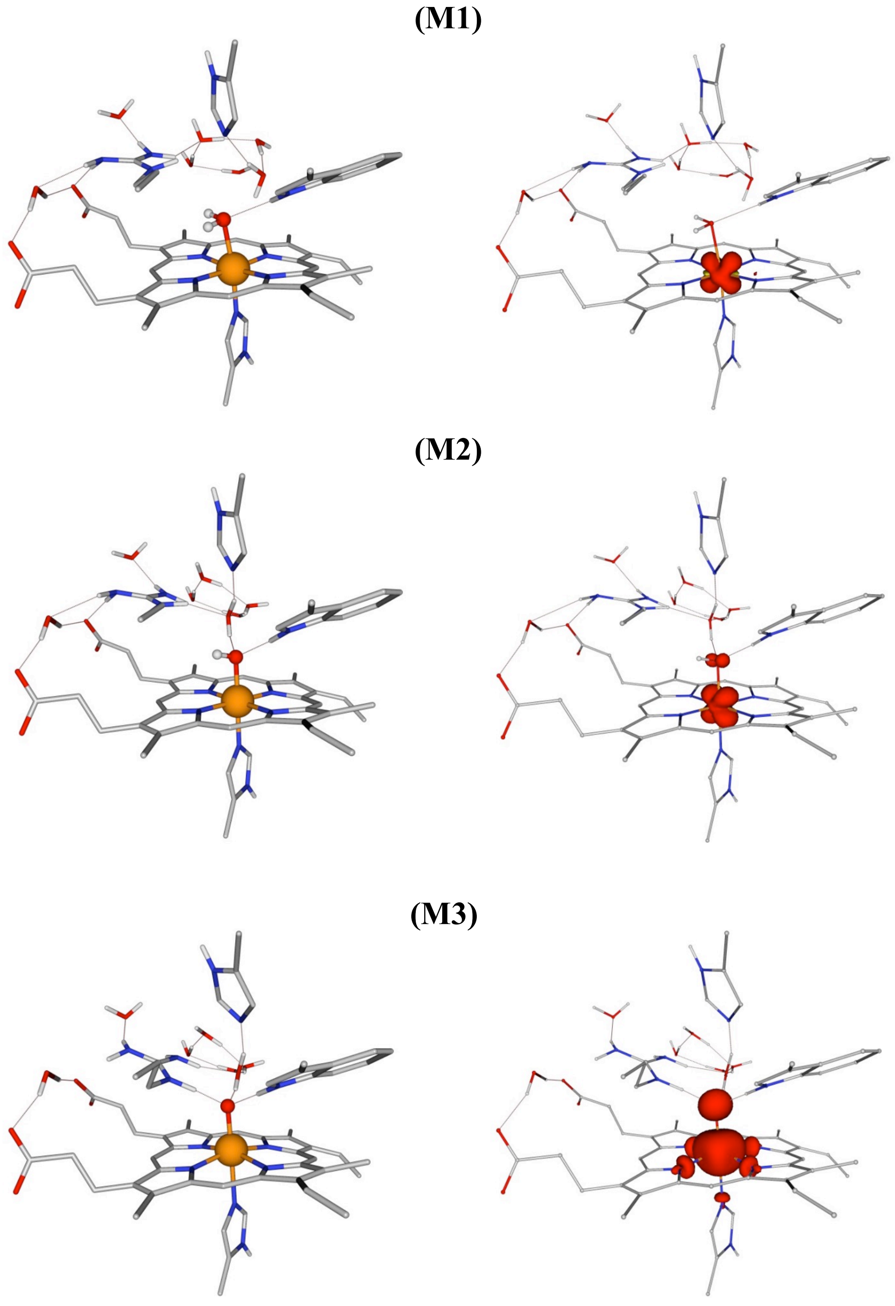

Figure S2. Optimized structures (left) and spin density plots (0.009 au, right) as calculated by DFT for the **M1**, **M2** and **M3** models of APX, i.e. explicitly constructed Fe(III) systems with H_2O, OH, and O axial ligands to Fe.

Table S2. Bond lengths and relative energies (in kJ mol^{-1}) for the **O1** and **OH1** model systems of APX-II computed at different spin states (superscript indicates the spin multiplicity) with the r^2SCAN-D4 and DLPNO-CCSD(T) methods.

**models**	**Fe-O (Å)**	**Δ*E* DFT**	**Δ*E* DLPNO-CCSD(T)**
^{5}**O1**	1.670	75.2	24.6
^{3}**O1**	1.671	0.0	0.0
^{1}**O1**	1.665	48.9	111.6
^{5}**OH1**	2.005	80.2	91.6
^{3}**OH1**	1.798	0.0	0.0
^{1}**OH1**	1.858	17.1	131.1

Table S3. Bond lengths and relative energies for the **O1-O8** and **OH1-OH8** model system in APX-II, calculated with r^2SCAN-D4/def2-TZVP.

Fe(IV)-oxo models	Fe-O (Å)	Δ*E* (kJ mol^{-1})	Fe(IV)-OH models	Fe-O (Å)	Δ*E* (kJ mol^{-1})
^{5}**O1**	1.670	75.2	^{5}**OH1**	2.005	80.0
^{3}**O1**	1.671	0.0	^{3}**OH1**	1.798	0.0
^{5}**O2**	1.674	74.4	^{5}**OH2**	2.057	74.0
^{3}**O2**	1.674	0.0	^{3}**OH2**	1.824	0.0
^{5}**O3**	1.657	72.1	^{5}**OH3**	2.017	72.0
^{3}**O3**	1.659	0.0	^{3}**OH3**	1.829	0.0
^{5}**O4**	1.667	72.1	^{5}**OH4**	2.004	76.9
^{3}**O4**	1.669	0.0	^{3}**OH4**	1.811	0.0
^{5}**O5**	1.711	80.1	^{5}**OH5**	2.817	24.5
^{3}**O5**	1.714	0.0	^{3}**OH5**	1.932	0.0
^{5}**O6**	1.709	78.5	^{5}**OH6**	3.205	6.3
^{3}**O6**	1.711	0.0	^{3}**OH6**	1.906	0.0
^{5}**O7**	1.676	76.9	^{5}**OH7**	2.916	45.2
^{3}**O7**	1.678	0.0	^{3}**OH7**	1.898	0.0
^{5}**O8**	1.696	76.7	^{5}**OH8**	2.161	52.3
^{3}**O8**	1.699	0.0	^{3}**OH8**	1.915	0.0

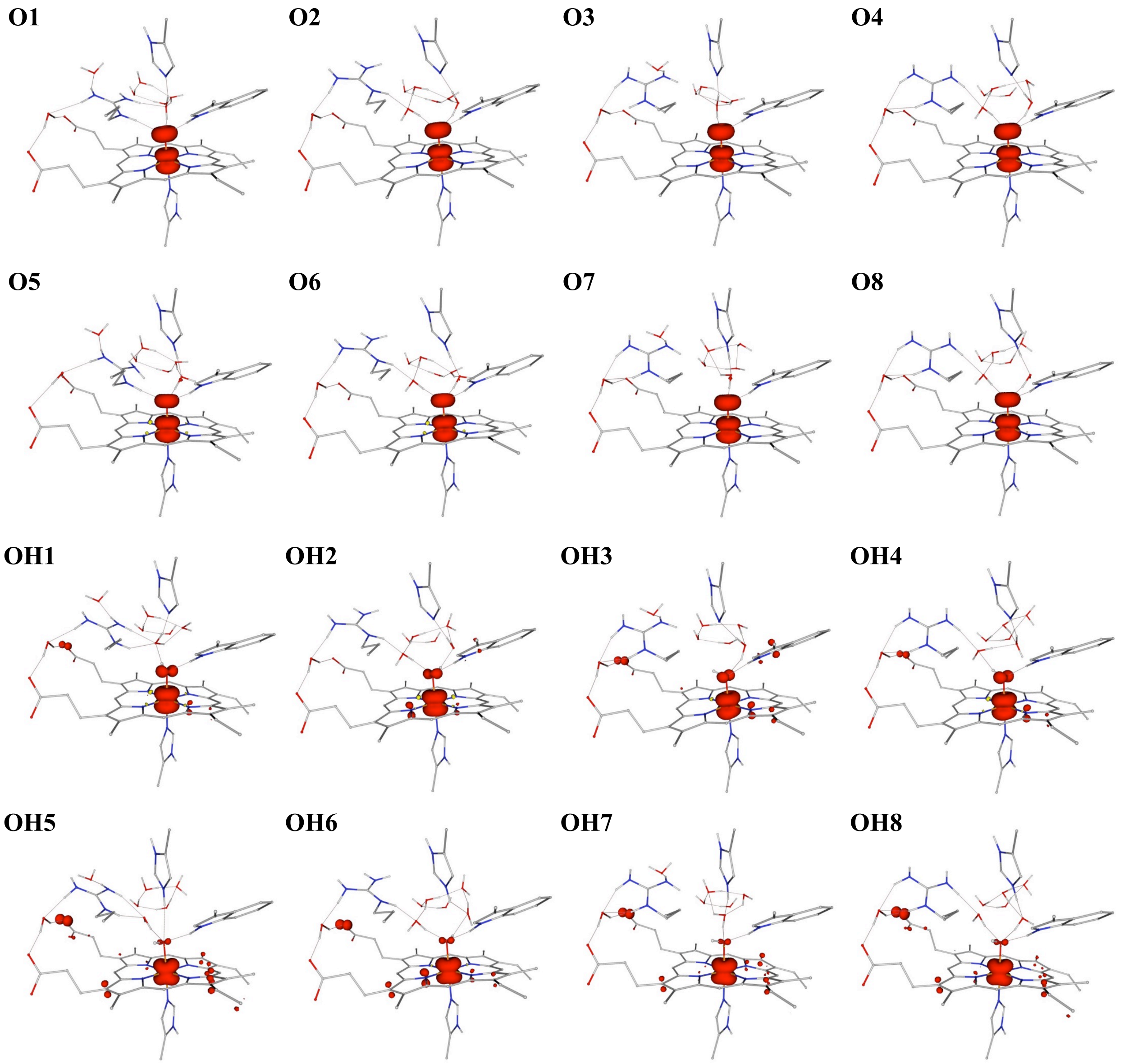

Figure S3. Spin density plots (0.009 au) for the **O1**-**O8** and **OH1**-**OH8** models, computed using r^{2}SCAN-D4/def2-TZVP.

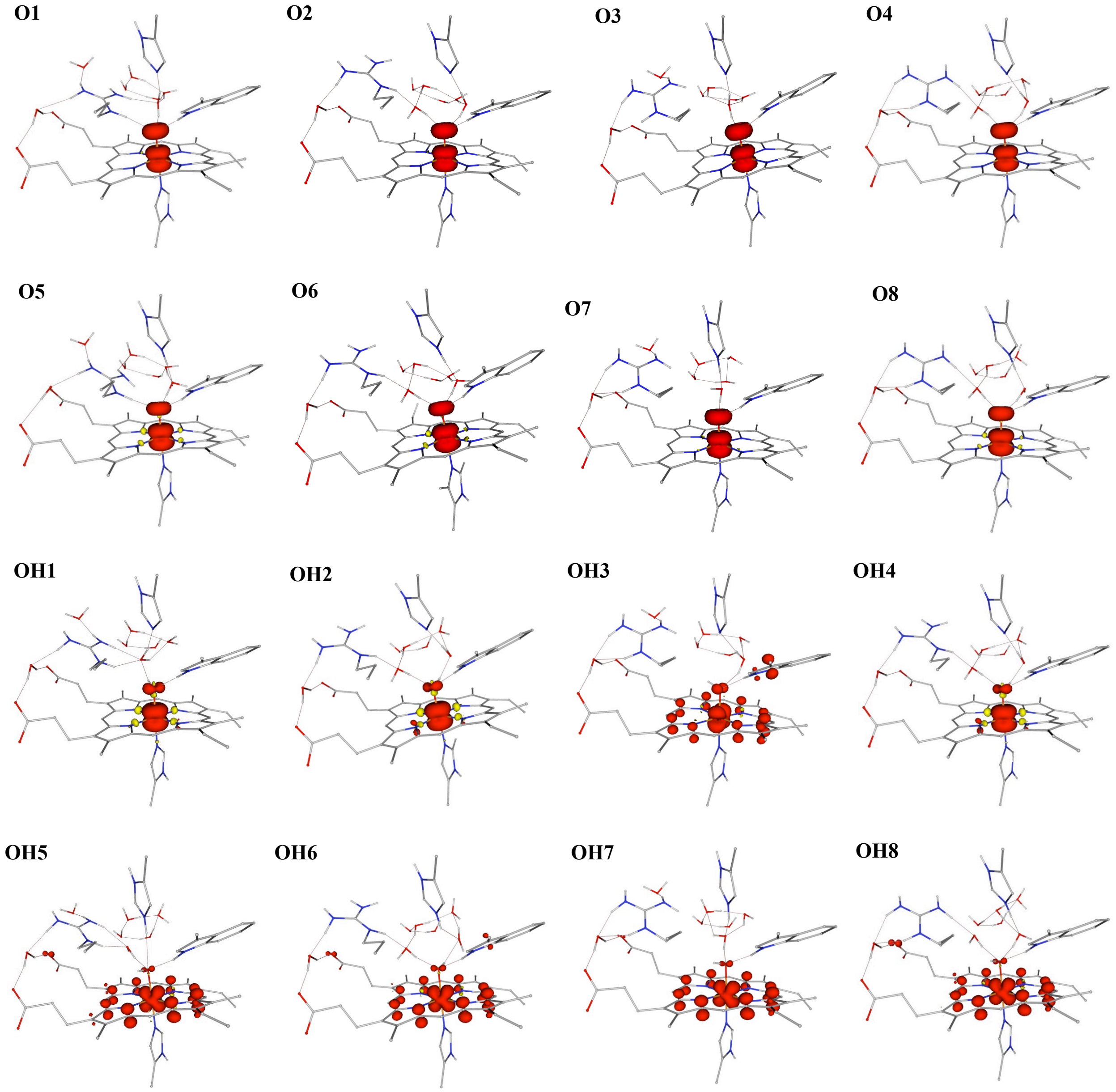

Figure S4. Spin density plots (0.009 au) for the **O1-O8** and **OH1-OH8** models, computed using B3LYP-D3(BJ)/def2-TZVP.

Table S4. Mulliken spin populations of the ground-state (S = 1) optimized Fe(IV)-oxo/hydroxo models computed wih r^2SCAN-D4/def2-TZVP.

Fe(IV)-oxo models	σ_{Fe}	σ_{O}	σ_{Por}	Fe(IV)-OH models	σ_{Fe}	σ_{O}	σ_{Por}
**O1**	1.53	0.53	-0.07	**OH1**	1.60	0.12	0.21
**O2**	1.53	0.53	-0.07	**OH2**	1.59	0.09	0.17
**O3**	1.47	0.60	-0.08	**OH3**	1.47	0.09	0.28
**O4**	1.49	0.58	-0.07	**OH4**	1.56	0.12	0.22
**O5**	1.67	0.35	-0.04	**OH5**	1.32	0.01	0.55
**O6**	1.66	0.36	-0.05	**OH6**	1.41	0.03	0.43
**O7**	1.58	0.48	-0.07	**OH7**	1.40	0.01	0.46
**O8**	1.62	0.43	-0.06	**OH8**	1.35	0.02	0.52

Table S5. Optimized bond lengths of the Fe(IV)-hydroxo/oxo model systems of APX-II computed with r^2SCAN-D4/def2-TZVP.

Models	Fe-O (Å)	Fe-NE2$_{His167}$	O-NE1$_{Trp41}$	O-NE$_{Arg38}$	O-NH1$_{Arg38}$
**O1**	1.671	2.105	2.716	3.096	4.116
**O2**	1.674	2.103	2.715	4.784	6.387
**O3**	1.659	2.104	2.669	5.203	5.355
**O4**	1.669	2.117	2.759	5.115	5.089
**O5**	1.714	2.070	2.735	2.846	3.644
**O6**	1.711	2.068	2.704	4.849	6.432
**O7**	1.678	2.082	2.652	5.285	5.421
**O8**	1.699	2.089	2.737	5.213	5.145
**OH1**	1.798	2.018	2.662	3.766	4.975
**OH2**	1.824	2.012	2.761	4.809	6.393
**OH3**	1.829	2.012	2.694	5.252	5.374
**OH4**	1.811	2.022	2.675	5.151	5.209
**OH5**	1.932	1.970	2.729	4.230	5.423
**OH6**	1.906	1.979	2.732	4.826	6.362
**OH7**	1.898	1.959	2.714	5.303	5.416
**OH8**	1.915	1.970	2.765	5.169	5.133

[a] Exp: Fe–O: 1.68[1]/1.87[2]/1.88[3]/1.84[4]; Fe-NE2$_{His167}$: 2.02[2]/1.97[3]; O-NE1$_{Trp41}$: 2.78[4]

Table S6. Bond lengths, relative energies, and spin populations of the Fe(III) **M1**, **M2** and **M3** models computed with r^2SCAN-D4/def2-TZVP.

models	Fe-O (Å)	ΔE (kJ mol^{-1})	σ_{Fe}	σ_{O}	σ_{Por}
^{6}**M1**	2.932	8.5	4.18	0.01	0.66
^{4}**M1**	2.842	0.7	2.78	0.01	0.05
^{2}**M1**	2.119	0.0	0.96	-0.01	0.04
^{6}**M2**	1.975	64.8	4.29	0.15	0.46
^{6}**M2**	2.023	65.1	2.84	0.13	-0.05
^{2}**M2**	1.869	0.0	1.00	0.05	-0.05
^{6}**M3**	1.837	0.0	4.19	0.38	0.36
^{4}**M3**	1.738	27.4	2.64	0.18	0.17
^{2}**M3**	1.781	14.5	0.98	0.19	-0.18

Table S7. Selected bond lengths of the **M1**, **M2**, **M3** models computed with r^2SCAN-D4/def2-TZVP.

models	Fe-O (Å)	Fe-NE2$_{His167}$	O-NE1$_{Trp41}$	O-NE$_{Arg38}$	O-NH1$_{Arg38}$
^{2}**M1**	2.119	1.913	2.880	3.463	4.729
^{2}**M2**	1.869	1.987	2.693	3.711	4.710
^{6}**M3**	1.837	2.431	2.597	2.624	3.700

[a] Exp: Fe–O: 1.68[1]/1.87[2]/1.88[3]/1.84[4]/2.08[4]; Fe-NE2$_{His167}$: 2.02[2]/1.97[3]; O-NE1$_{Trp41}$: 2.78[4]

Table S8. Bond lengths of the optimized Fe(IV)-oxo/hydroxo ground state (S = 1) models calculated with r^2SCAN-D3BJ/def2-TZVP.

Fe(IV)-oxo models	Fe-O (Å)	Fe(IV)-OH models	Fe-O (Å)
**O1**	1.671	**OH1**	1.798
**O2**	1.674	**OH2**	1.796
**O3**	1.659	**OH3**	1.829
**O4**	1.669	**OH4**	1.824
**O5**	1.714	**OH5**	1.944
**O6**	1.711	**OH6**	1.906
**O7**	1.678	**OH7**	1.898
**O8**	1.688	**OH8**	1.930
**Exp.**	1.68[1]	**Exp.**	1.87[2]

Table S9. Bond lengths of the optimized Fe(IV)-oxo/hydroxo ground state (S = 1) models calculated with B3LYP-D3BJ/def2-TZVP.

Fe(IV)-oxo models	Fe-O (Å)	Fe(IV)-OH models	Fe-O (Å)
**O1**	1.662	**OH1**	1.773
**O2**	1.664	**OH2**	1.796
**O3**	1.650	**OH3**	1.856
**O4**	1.660	**OH4**	1.786
**O5**	1.697	**OH5**	1.932
**O6**	1.697	**OH6**	1.974
**O7**	1.665	**OH7**	1.907
**O8**	1.688	**OH8**	1.915
**Exp.**	1.68[1]	**Exp.**	1.87[2]

Table S10. Bond lengths of the optimized Fe(IV)-oxo/hydroxo ground state (S = 1) models calculated with B3LYP-D4/def2-TZVP.

Fe(IV)-oxo models	Fe-O (Å)	Fe(IV)-OH models	Fe-O (Å)
**O1**	1.662	**OH1**	1.773
**O2**	1.664	**OH2**	1.795
**O3**	1.649	**OH3**	1.856
**O4**	1.659	**OH4**	1.784
**O5**	1.697	**OH5**	1.944
**O6**	1.696	**OH6**	1.906
**O7**	1.666	**OH7**	1.906
**O8**	1.687	**OH8**	1.931
**Exp.**	1.68[1]	**Exp.**	1.87[2]

Table S11. Mulliken spin populations of the optimized Fe(IV)-oxo/hydroxo for ground state (S = 1) models calculated with B3LYP-D3BJ/def2-TZVP.

Fe(IV)-oxo models	σ_{Fe}	σ_{O}	σ_{Por}	Fe(IV)-OH models	σ_{Fe}	σ_{O}	σ_{Por}
**O1**	1.59	0.51	-0.09	**OH1**	1.92	0.12	-0.04
**O2**	1.58	0.52	-0.09	**OH2**	1.93	0.09	-0.02
**O3**	1.50	0.59	-0.09	**OH3**	1.04	0.04	0.72
**O4**	1.53	0.57	-0.09	**OH4**	1.91	0.12	-0.02
**O5**	1.75	0.34	-0.10	**OH5**	1.05	0.01	0.88
**O6**	1.74	0.36	-0.11	**OH6**	1.06	0.0	0.79
**O7**	1.62	0.48	-0.10	**OH7**	1.04	0.01	0.88
**O8**	1.69	0.42	-0.10	**OH8**	1.05	0.01	0.87

Table S12. Relaxed scans between Fe(IV) and O atoms of the **O1** and **OH1** model calculated using r^{2}SCAN-D4/def2-TZVP, and corresponding DLPNO-CCSD(T) energies.

Fe-O (**O1**)	ΔE	DLPNO-CCSD(T)	Fe-O (**OH1**)	ΔE	DLPNO-CCSD(T)
1.601	8.98	9.03	1.718	5.42	7.00
1.621	4.35	4.22	1.738	2.92	3.50
1.641	1.49	1.47	1.758	1.12	3.88
1.661	0.19	0.16	1.778	0.16	0.23
1.671	0.00	0.00	1.798	0.00	0.00
1.681	0.10	0.04	1.818	0.08	0.22
1.701	1.11	1.21	1.838	0.99	6.52
1.721	3.03	4.37	1.858	2.26	6.38
1.741	5.62	7.53	1.878	3.93	7.79

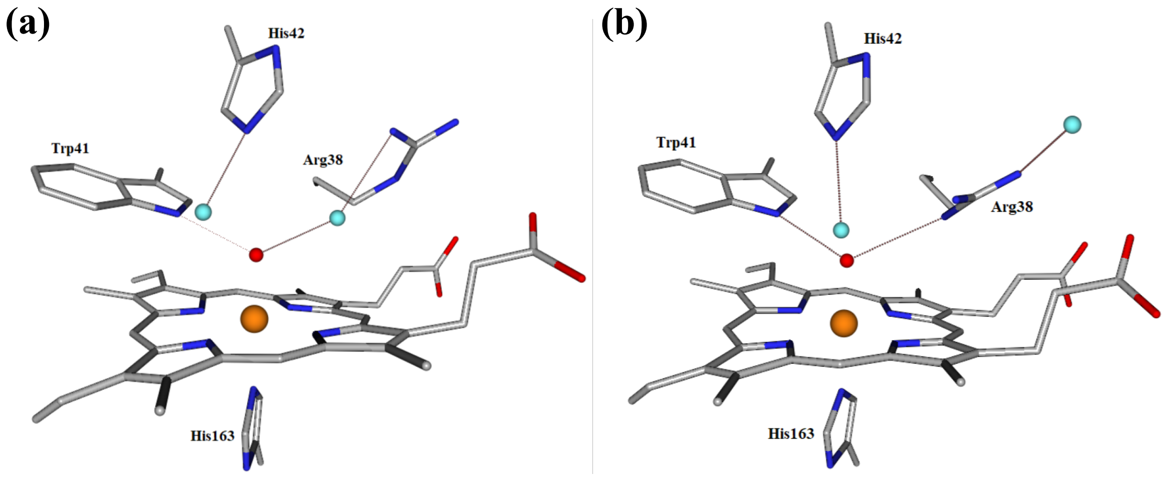

Figure S5. Arg38 can be observed in two positions: one "out" position (a) and the other "in" (b).[2-3] This is correlated to the position of a water molecule which is then reversely "in" or "out". The two positions are not a simple shift but a rotation along the C_γ–C_δ bond. The ferryl oxygen is depicted in red, selected waters in cyan.

Table S13. Relative energies for distinct sets of models with respect to the orientation of Arg38, calculated with r^{2}SCAN-D4/def2-TZVP.

Fe(IV)-oxo models	ΔE (kJ/mol)	Orientation of Arg38
**O1**	0.0	in
**O2**	-23.3	out
**O3**	25.7	in
**O4**	-11.5	out
**O5**	0.0	in
**O6**	-62.1	out
**O7**	20.1	in
**O8**	-46.1	out
**OH1**	0.0	in
**OH2**	-23.1	out
**OH3**	6.6	in
**OH4**	-26.3	out
**OH5**	0.0	in
**OH6**	-31.3	out
**OH7**	-15.6	in
**OH8**	-34.6	out

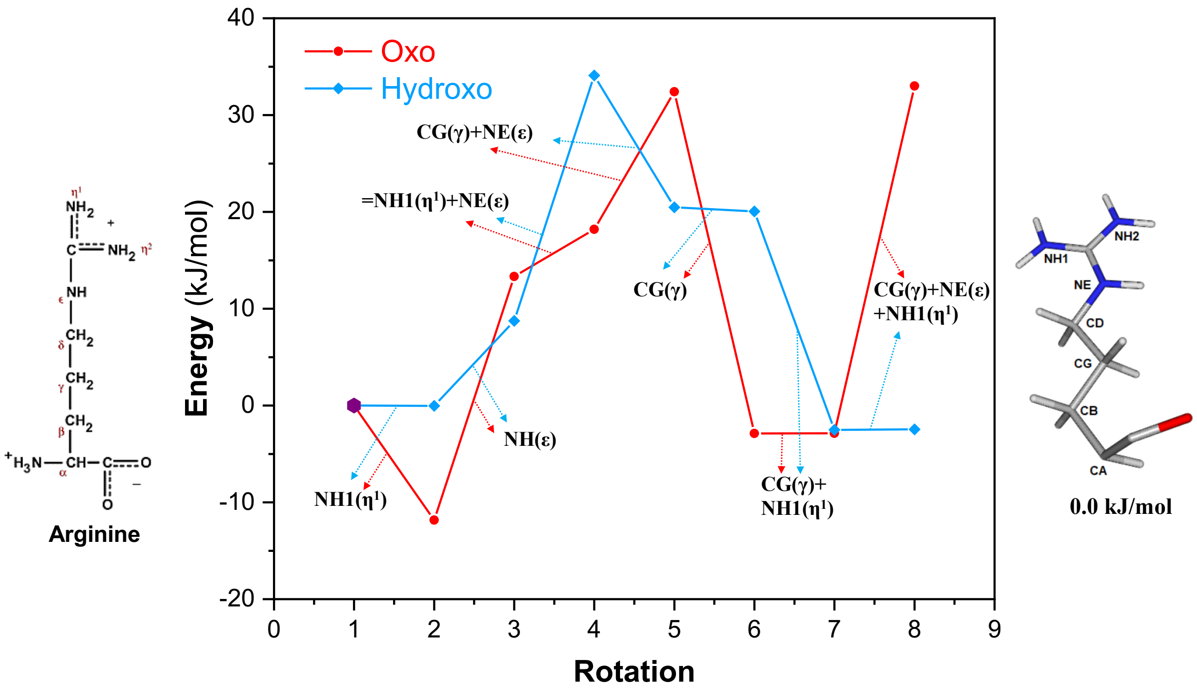

Figure S6. Relative energies of rotamers/conformers/charge states of Arg38 with oxo and hydroxo models.

Table S14. Bond lengths and energies of the optimized **M1**, **O1** and **OH1** models for proton movement from HH12 of Arg38 to NE2 in His42, calculated using r^{2}SCAN-D4/def2-TZVP.[5] Superscript "*" indicates the proton is in the NE2 position in His42.

models	Fe-O (Å)	Δ*E* (kJ/mol)
^{2}**M1**	2.119	0.0
^{2}**M1***	2.205	144.4
^{3}**O1**	1.671	0.0
^{3}**O1***	1.676	73.0
^{3}**OH1**	1.798	0.0
^{3}**OH1***	1.856	90.9

Table S15. Bond lengths and relative energies of the optimized **O1-O4** models for proton movement from ND1 to NE2 in His42, calculated using r^{2}SCAN-D4/def2-TZVP.
Superscript "*" indicates the proton is in the NE2 position in His42.

Fe(IV)-oxo models	Fe-O (Å)	Δ*E* (kJ/mol)
^{3}**O1**	1.671	0.0
^{3}**O1***	1.693	83.8
^{3}**O2**	1.674	0.0
^{3}**O2***	1.689	76.3
^{3}**O3**	1.659	0.0
^{3}**O3***	1.672	49.3
^{3}**O4**	1.669	0.0
^{3}**O4***	1.685	40.8

Table S16. Bond lengths and energies of the optimized **OH1-OH4** models for proton movement from ND1 to NE2 in His42, calculated using r^{2}SCAN-D4/def2-TZVP.
Superscript "*" indicates the proton is in the NE2 position in His42.

Fe(IV)-oxo models	Fe-O (Å)	Δ*E* (kJ/mol)
^{3}**OH1**	1.798	0.0
^{3}**OH1***	1.834	105.1
^{3}**OH2**	1.824	0.0
^{3}**OH2***	1.868	96.9
^{3}**OH3**	1.810	0.0
^{3}**OH3***	1.859	58.6
^{3}**OH4**	1.828	0.0
^{3}**OH4***	1.874	47.5

TableS17. Calculated Mössbauer parameters for all Fe(IV)-oxo/hydroxo models, compared to experimental Mössbauer data, with % deviations from the experimental data for APX-II.

Model	δ	% dev(δ)	ΔE_Q	% dev(ΔE_Q)	Model	δ	% dev(δ)	ΔE_Q	% dev(ΔE_Q)
**O1**	0.06	20	1.70	2.4	**OH1**	0.06	20	2.72	63.8
**O2**	0.06	20	1.72	3.6	**OH2**	0.08	60	2.81	69.3
**O3**	0.07	40	1.48	-10.8	**OH3**[a]	0.27	440	-2.71	-63.2
**O4**	0.07	40	1.64	-1.2	**OH4**	0.07	40	2.82	69.9
**O5**	0.05	0	2.11	27.1	**OH5**[a]	0.30	500	-2.59	-56.0
**O6**	0.05	0	2.05	23.5	**OH6**[a]	0.28	460	-2.54	-53.0
**O7**	0.06	20	1.72	3.6	**OH7**[a]	0.28	460	-2.73	-64.5
**O8**	0.06	20	1.95	17.5	**OH8**[a]	0.29	480	-2.53	-52.4
Exp	0.05		1.66		***Exp***	0.05		1.66	

Table S18. Calculated Mössbauer parameters (TPSS functional) for the models presented in Figure 4, compared to experimental Mössbauer data for the Fe(IV)-oxo/hydroxo main component.

model	δ	ΔE_Q	model	δ	ΔE_Q
**O1**	0.07	1.51	**OH1**	0.12	2.09
**O2**	0.07	1.53	**OH2**	0.14	2.16
**O3**	0.07	1.31	**OH3**[a]	0.15	2.29
**O4**	0.07	1.46	**OH4**	0.13	2.21
**O5**	0.07	1.83	**OH5**[a]	0.22	2.31
**O6**	0.07	1.78	**OH6**[a]	0.19	2.28
**O7**	0.06	1.51	**OH7**[a]	0.18	2.21
**O8**	0.07	1.72	**OH8**[a]	0.20	2.24
**Exp**	0.05[6]	1.66[6]			

Table S19. Mulliken spin populations of the optimized Fe(IV)-oxo/hydroxo model for the ground state (S = 1) were calculated using the TPSS-D3BJ/def2-TZVP level of theory.

Fe(IV)-oxo models	σ_{Fe}	σ_O	σ_{Por}	Fe(IV)-OH models	σ_{Fe}	σ_O	σ_{Por}
**O1**	1.55	0.54	-0.10	**OH1**	1.57	0.14	0.21
**O2**	1.54	0.53	-0.10	**OH2**	1.55	0.09	0.19
**O3**	1.49	0.60	-0.11	**OH3**	1.46	0.10	0.28
**O4**	1.50	0.58	-0.10	**OH4**	1.53	0.13	0.22
**O5**	1.67	0.36	-0.06	**OH5**	1.34	0.01	0.53
**O6**	1.66	0.37	-0.07	**OH6**	1.41	0.02	0.43
**O7**	1.59	0.49	-0.10	**OH7**	1.42	0.01	0.44
**O8**	1.62	0.44	-0.09	**OH8**	1.37	0.01	0.49

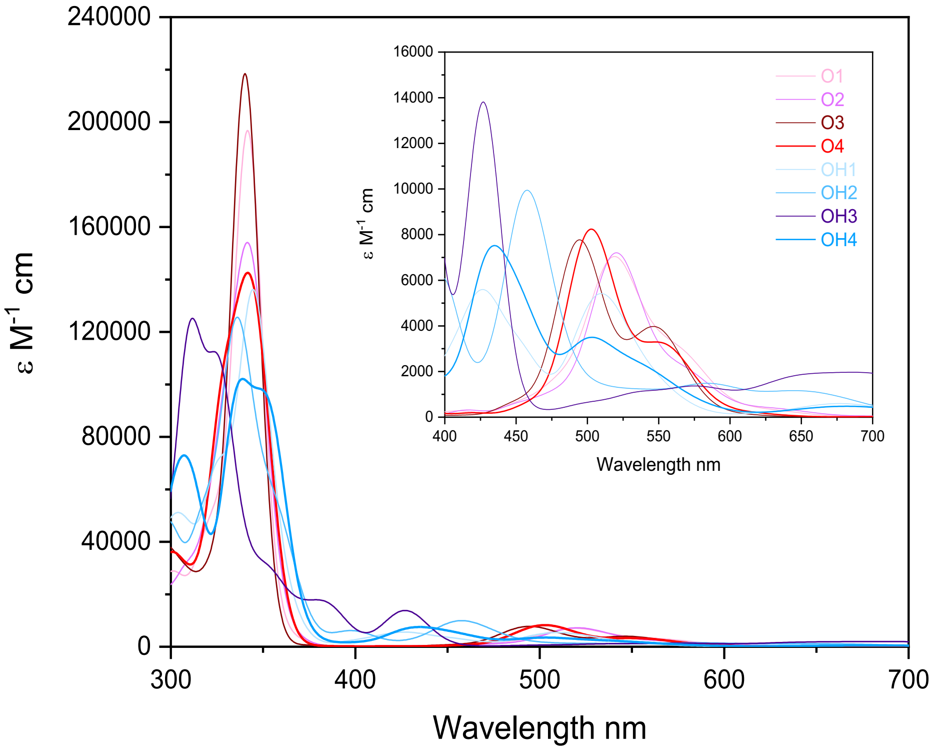

Figure S7. Absorption spectra computed using TD-DFT calculations on the ground state structures of **O1-O4** and **OH1-OH4** of APX-II.

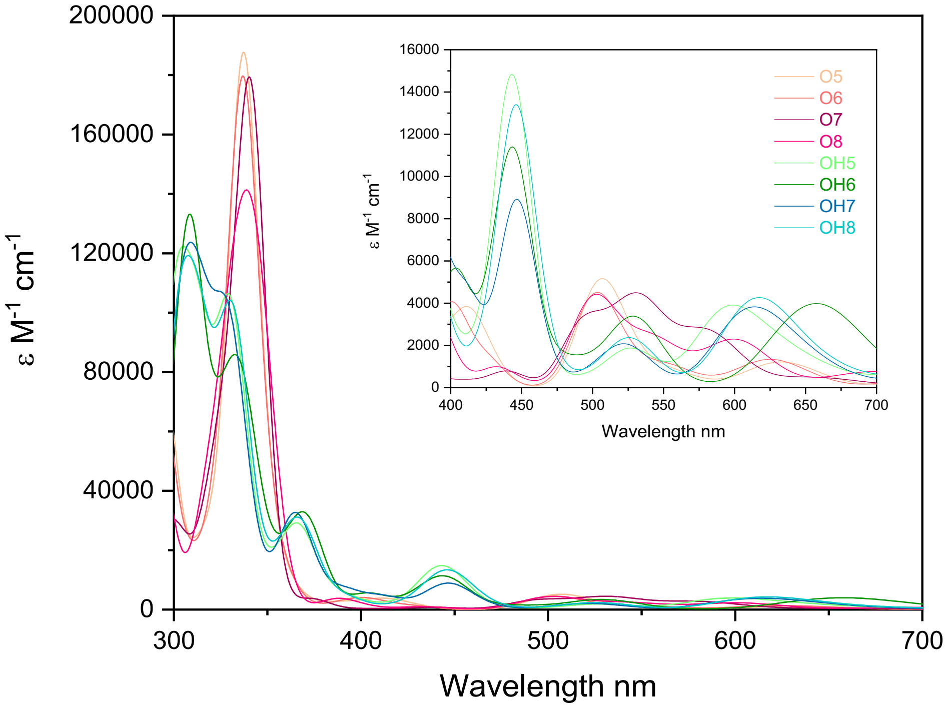

Figure S8. Absorption spectra computed using TD-DFT calculations on the ground state structures of **O5-O8** and **OH5-OH8** of APX-II.

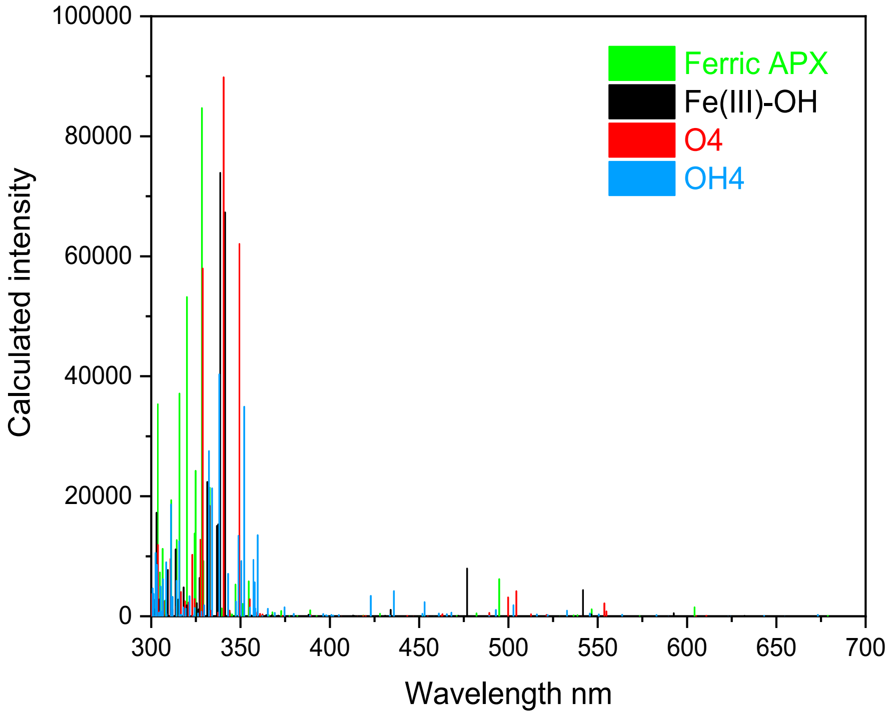

Figure S9. The electronic transitions of the selected models shown as stick spectra.

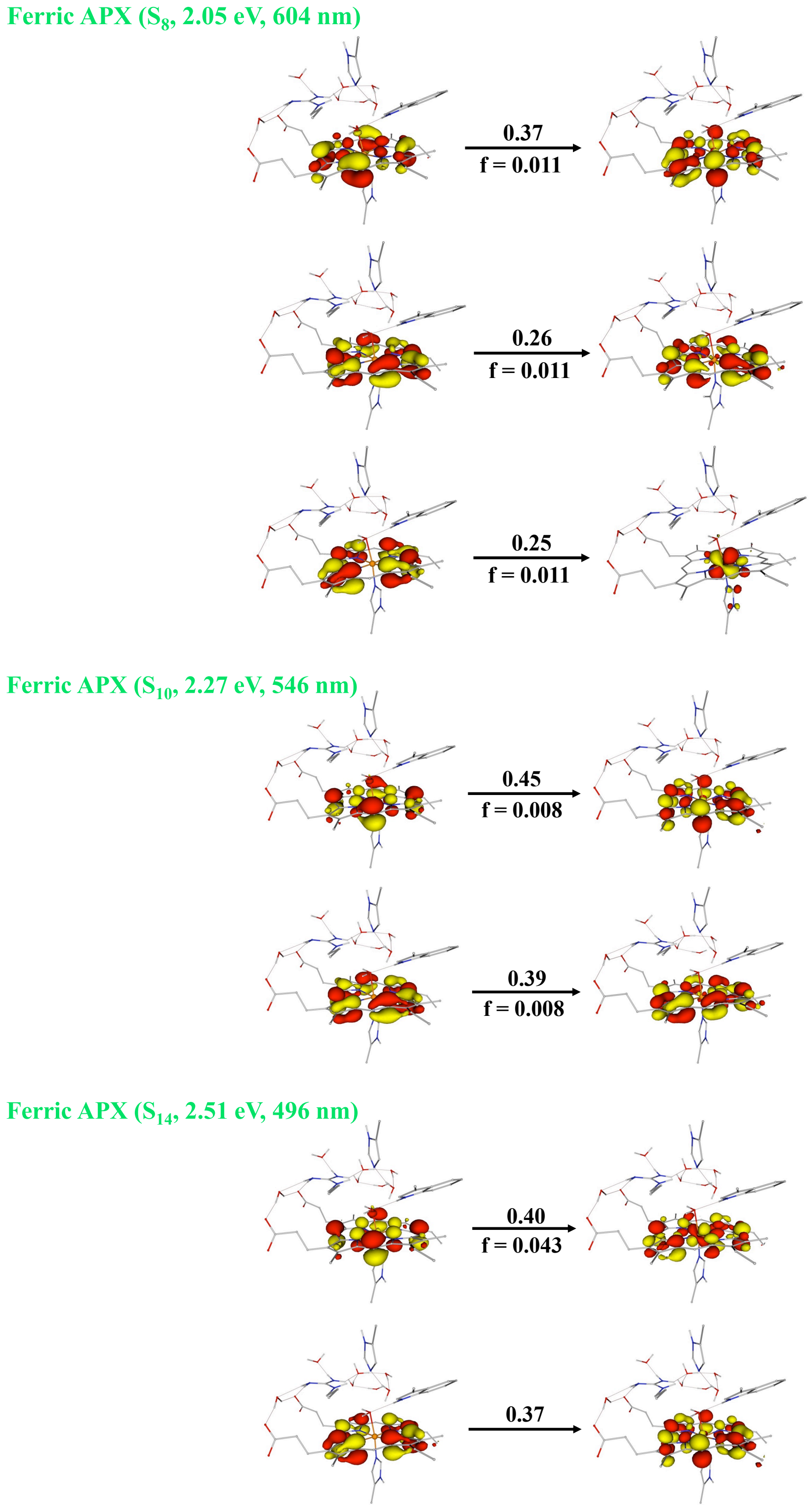

Figure S10. Plots of NTOs (isovalue = 0.04) for key electronic transitions in **Ferric APX**, **Fe(III)-OH**, **O4**, and **OH4**. Excitation wavelengths are given along with the weights of NTOs pairs. The panel to the left of the arrow shows hole density and the panel to the right of the arrow shows particle density.

Fe(III)-OH (S_{11}, 2.29 eV, 542 nm)

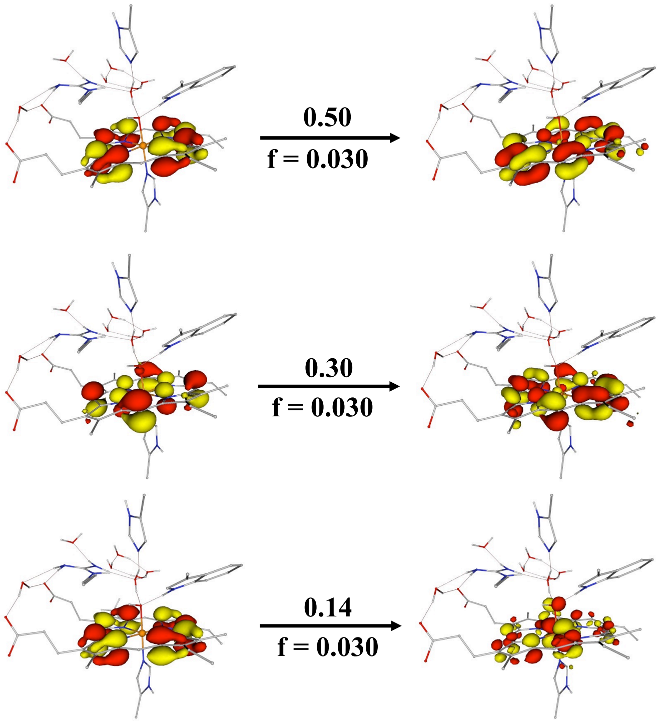

Fe(III)-OH (S_{13}, 2.60 eV, 477 nm)

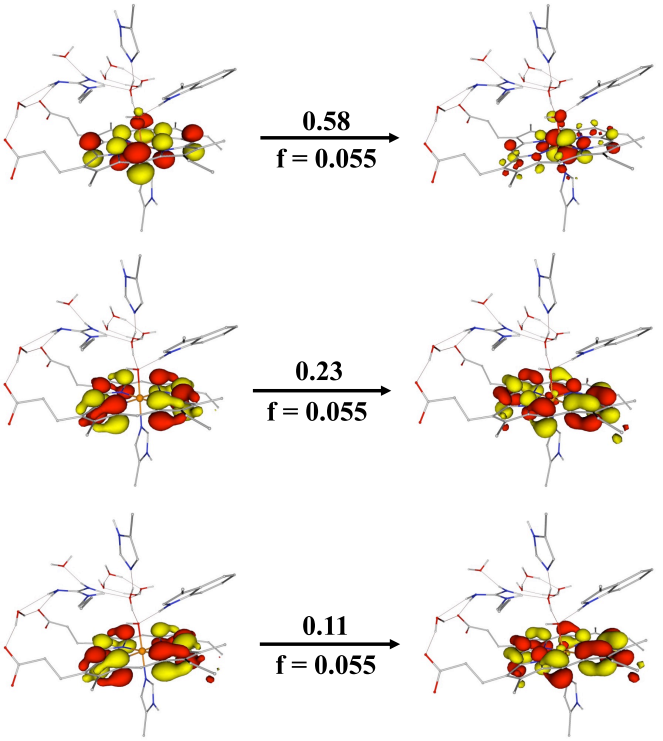

Figure S10. (Continued)

O4 (S_7, 2.23 eV, 555 nm)

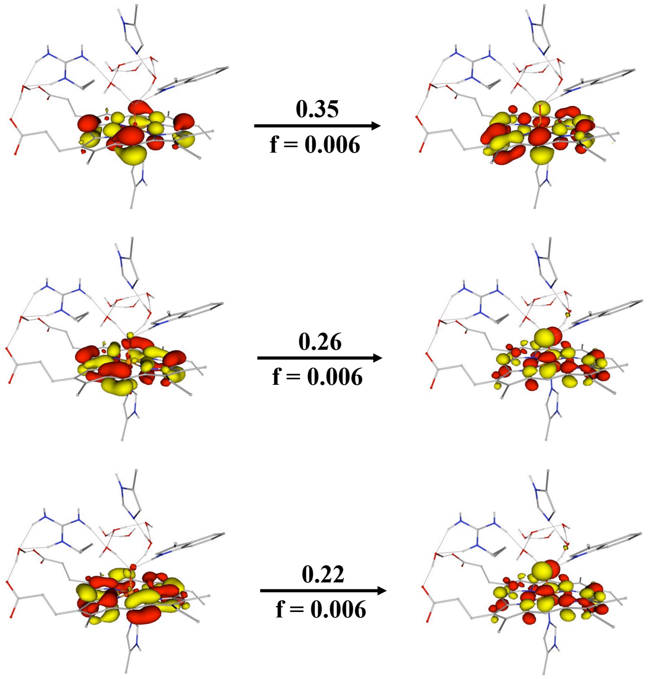

O4 (S_8, 2.24 eV, 554 nm)

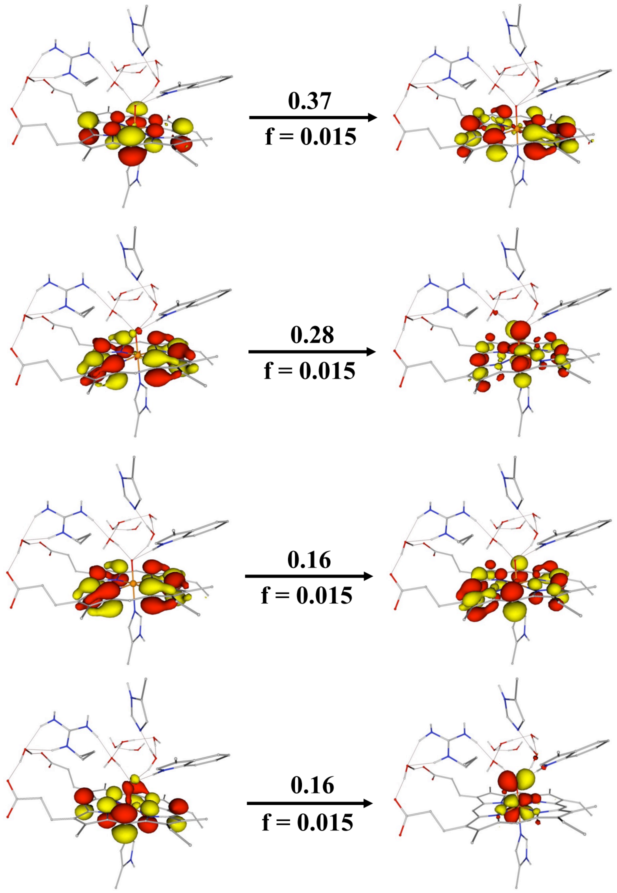

Figure S10. (Continued)

O4 (S_{12}, 2.46 eV, 504 nm)

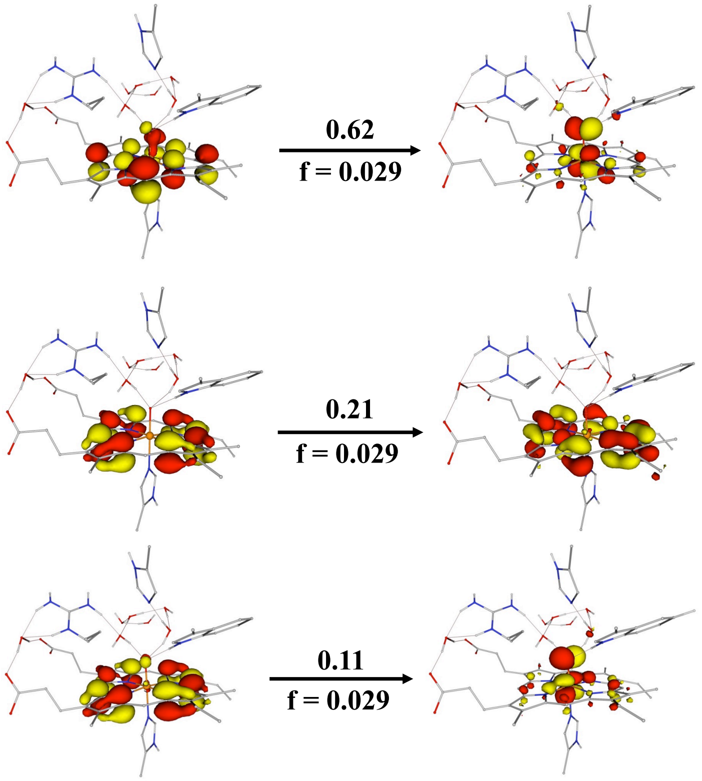

O4 (S_{13}, 2.48 eV, 500 nm)

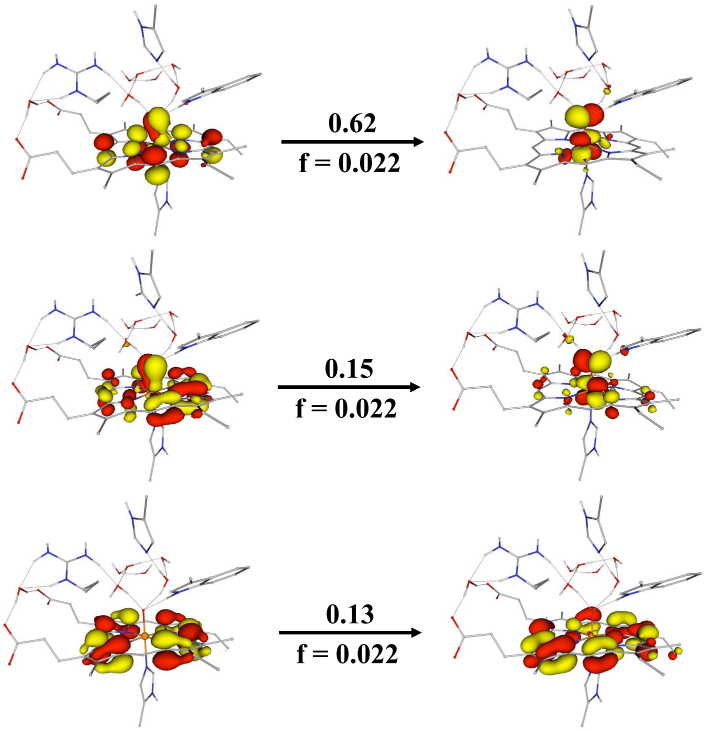

Figure S10. (Continued)

OH4 (S_8, 1.84 eV, 674 nm)

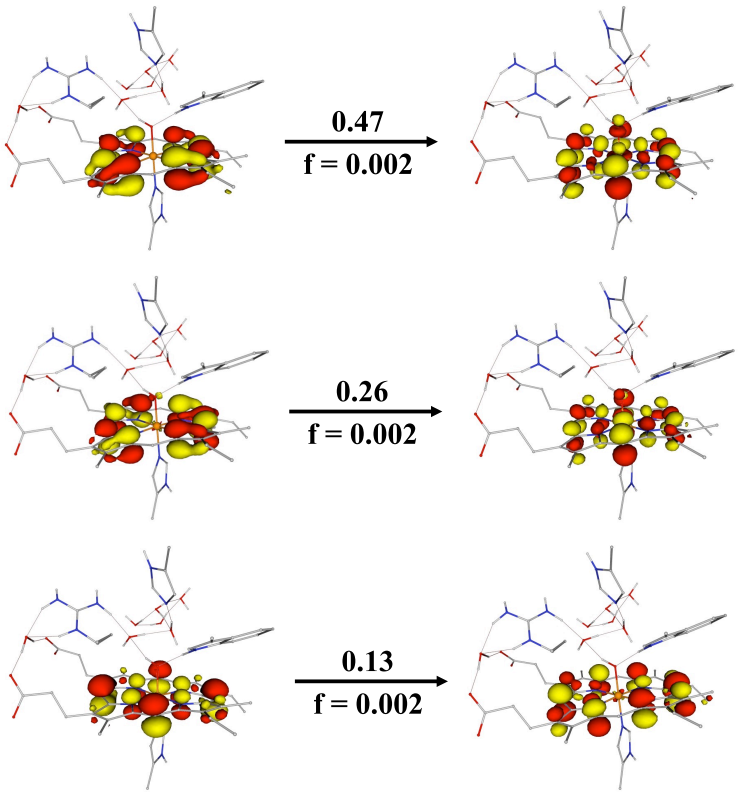

OH4 (S_{18}, 2.32 eV, 533 nm)

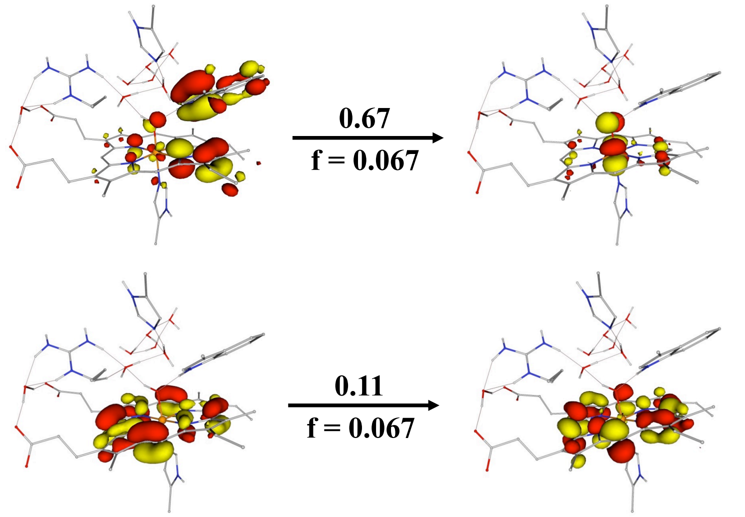

Figure S10. (Continued)

OH4 (S_{21}, 2.46 eV, 503 nm)

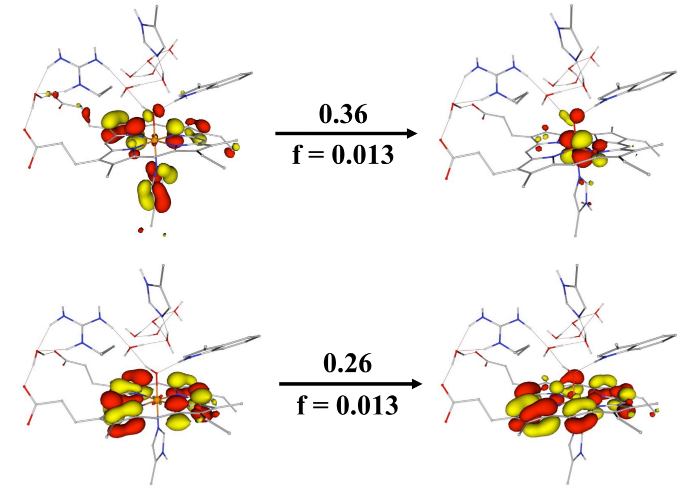

OH4 (S_{28}, 2.73 eV, 453 nm)

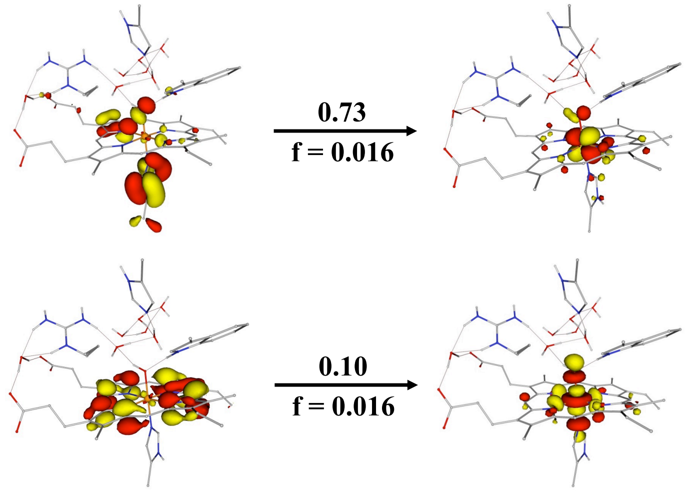

OH4 (S_{30}, 2.84 eV, 436 nm)

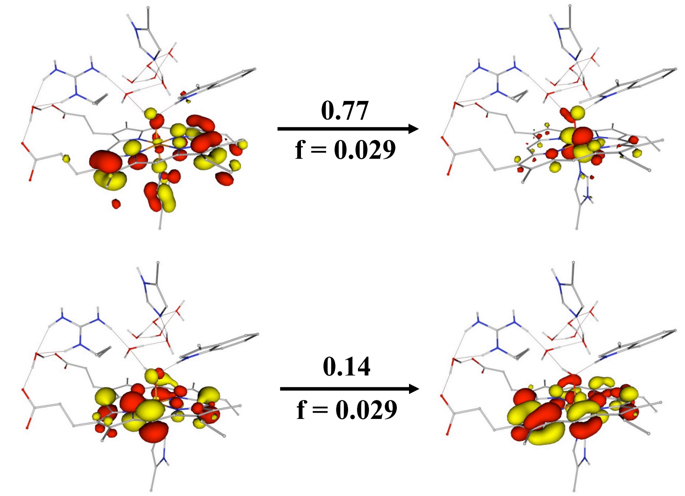

Figure S10. (Continued)

OH4 (S_{32}, 2.93 eV, 423 nm)

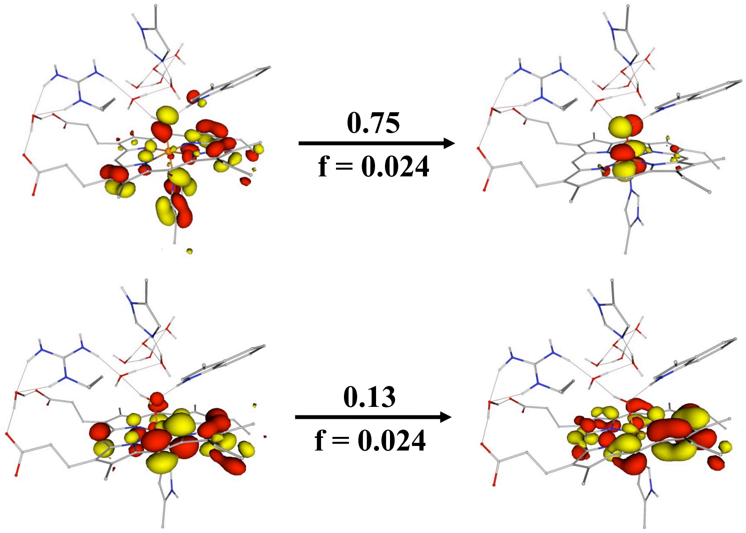

Figure S10. (Continued).

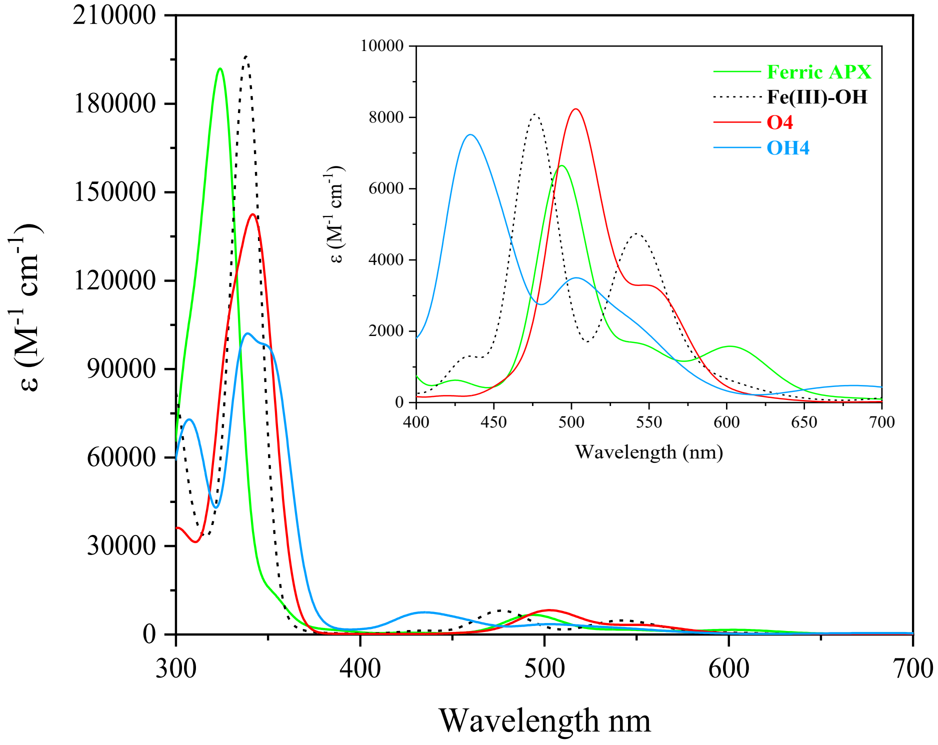

Figure S11. Absorption spectra computed using TD-DFT calculations on the ground state structures of the **Ferric APX**, **Fe(III)-OH**, **O4** and **OH4** of APX-II.

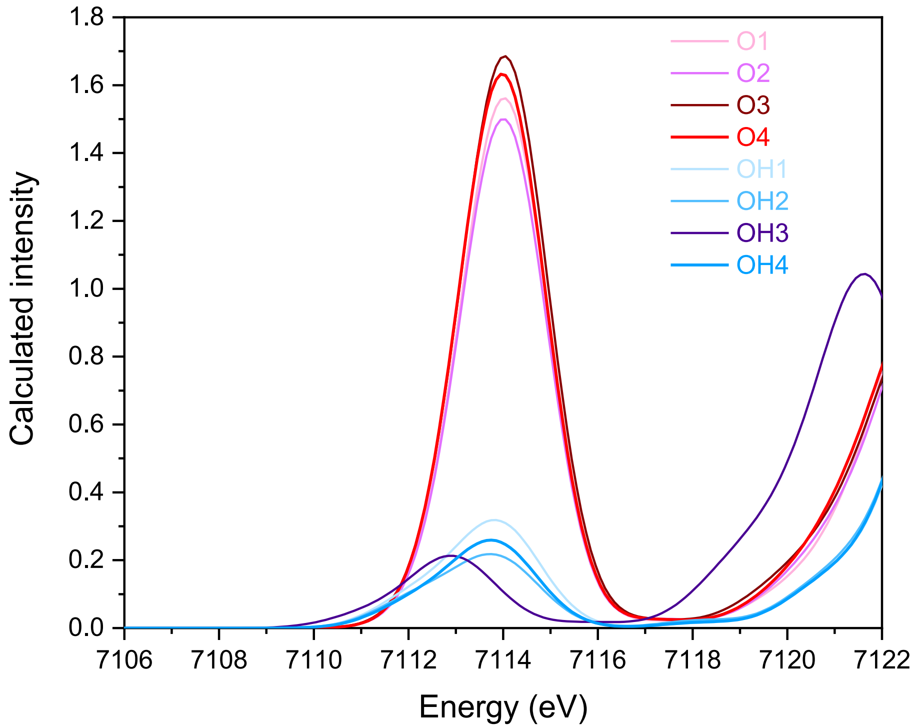

Figure S12. Calculated TDDFT Fe K pre-edge XAS for **O1-O4** and **OH1-OH4** of APX-II.

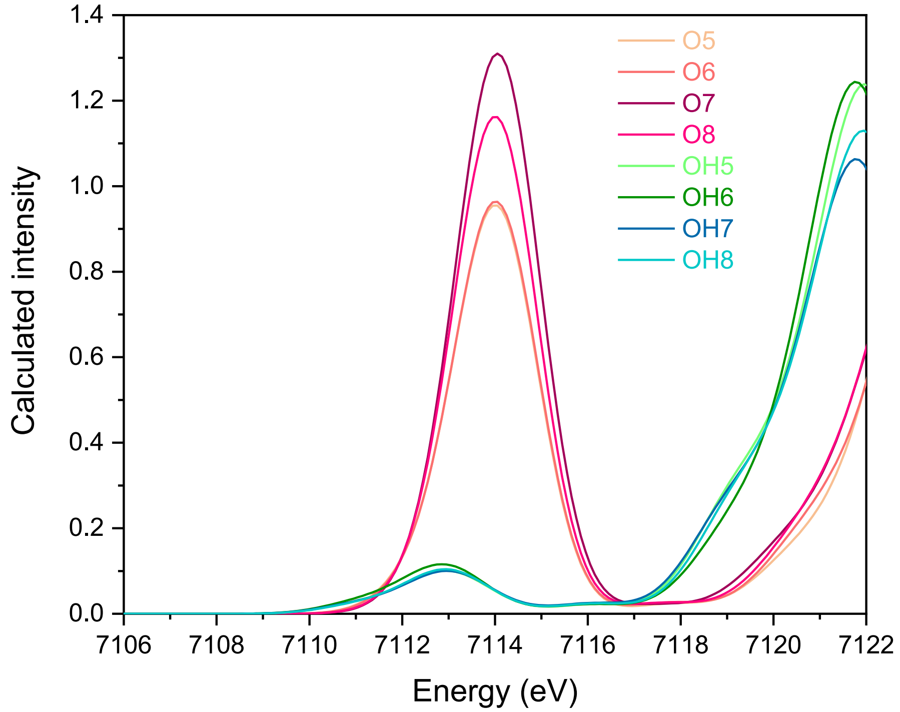

Figure S13. Calculated TDDFT Fe K pre-edge XAS for **O5-O8** and **OH5-OH8** of APX-II.

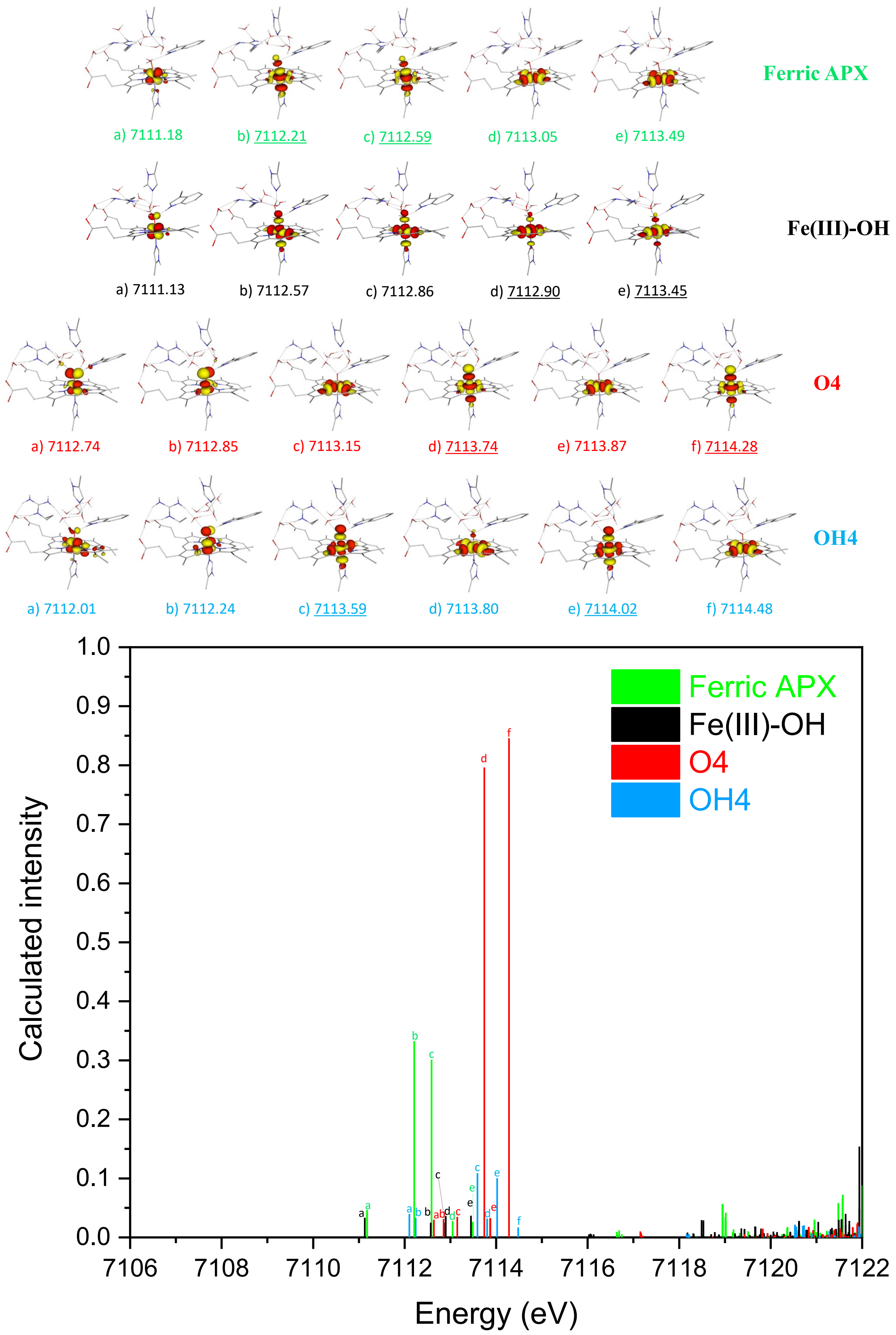

Figure S14. Assignment of the calculated pre-edge XAS spectrum based on the NTOs (isovalue = 0.04) associated with the transitions for **Ferric APX**, **Fe(III)-OH**, **O4** and **OH4** of APX-II.

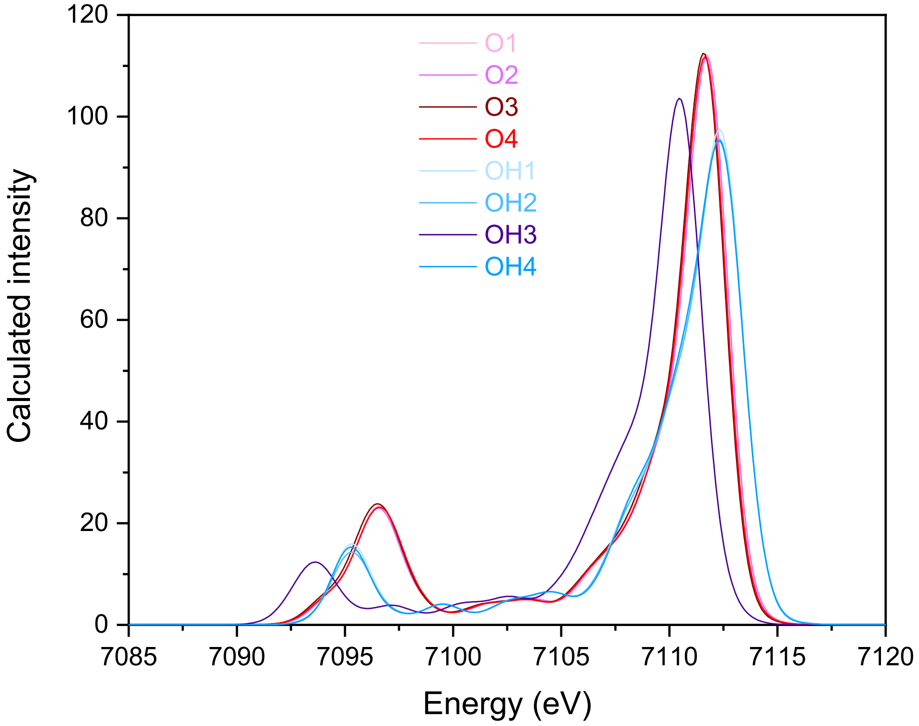

Figure S15. Calculated valence to core spectra for **O1-O4** and **OH1-OH4** of APX-II.

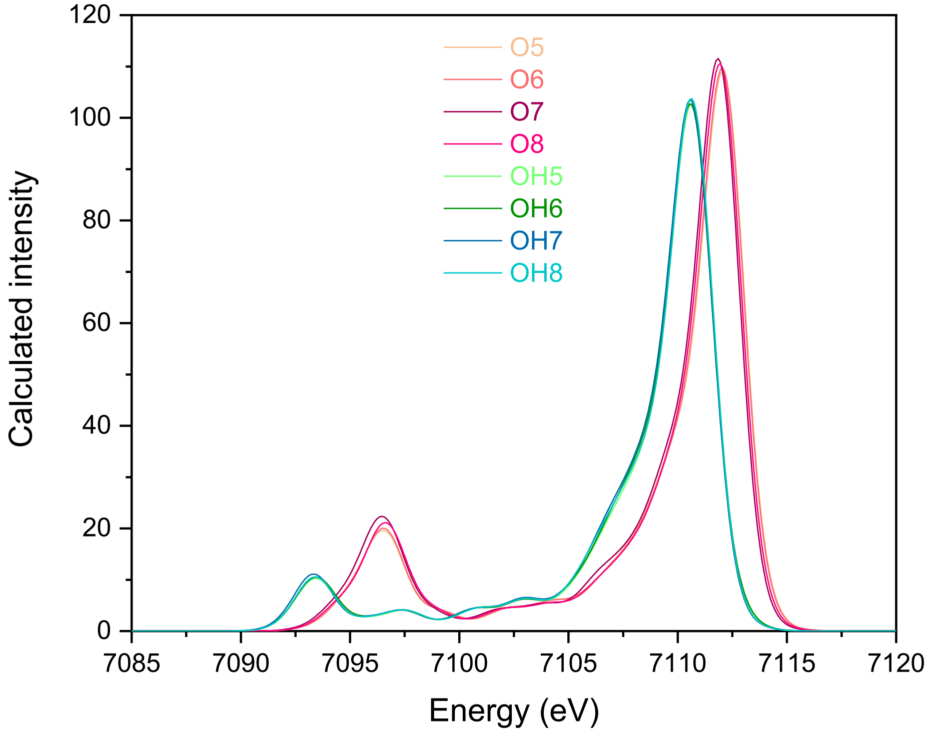

Figure S16. Calculated valence to core spectra for **O5-O8** and **OH5-OH8** of APX-II.

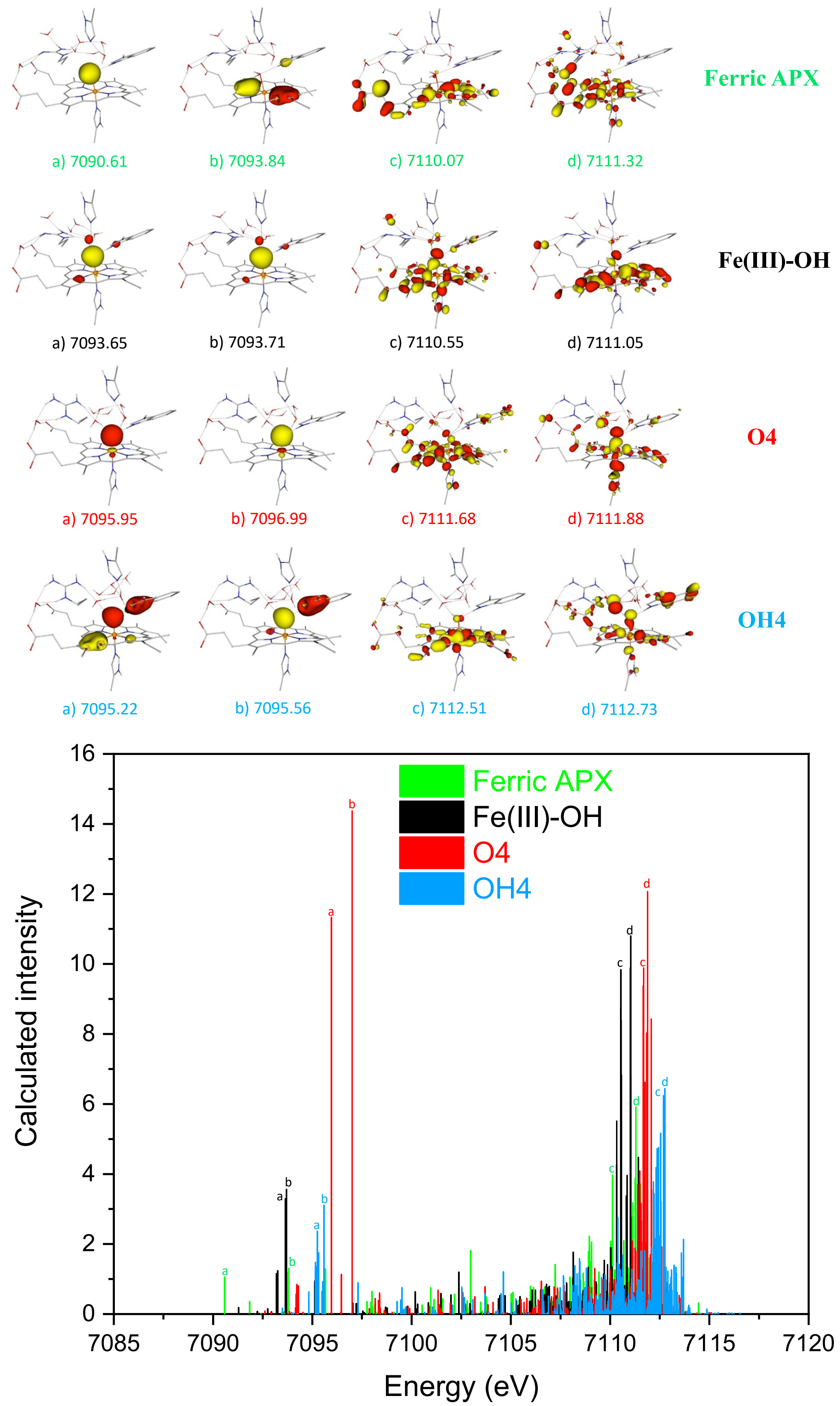

Figure S17. Assignment of selected XES transitions based on molecular orbitals (isovalue=0.04) for **Ferric APX**, **Fe(III)-OH**, **O4** and **OH4** of APX-II.

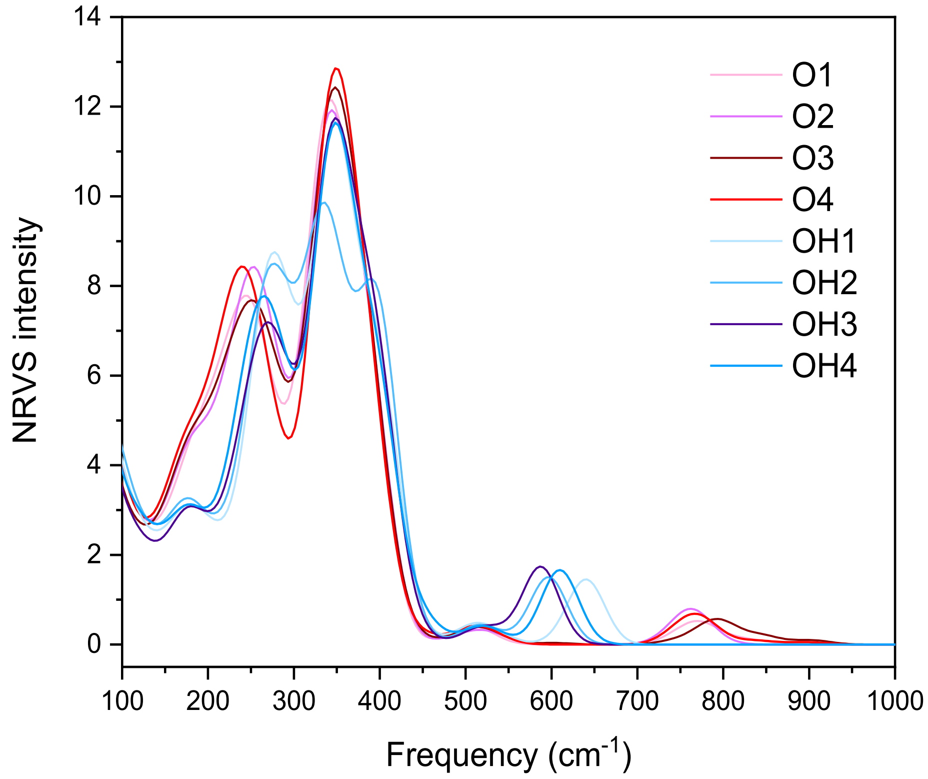

Figure S18. NRVS spectra of the **O1-O4** and **OH1-OH4** of APX-II computed with r^{2}SCAN/def2-TZVP (T = 298.15 K).

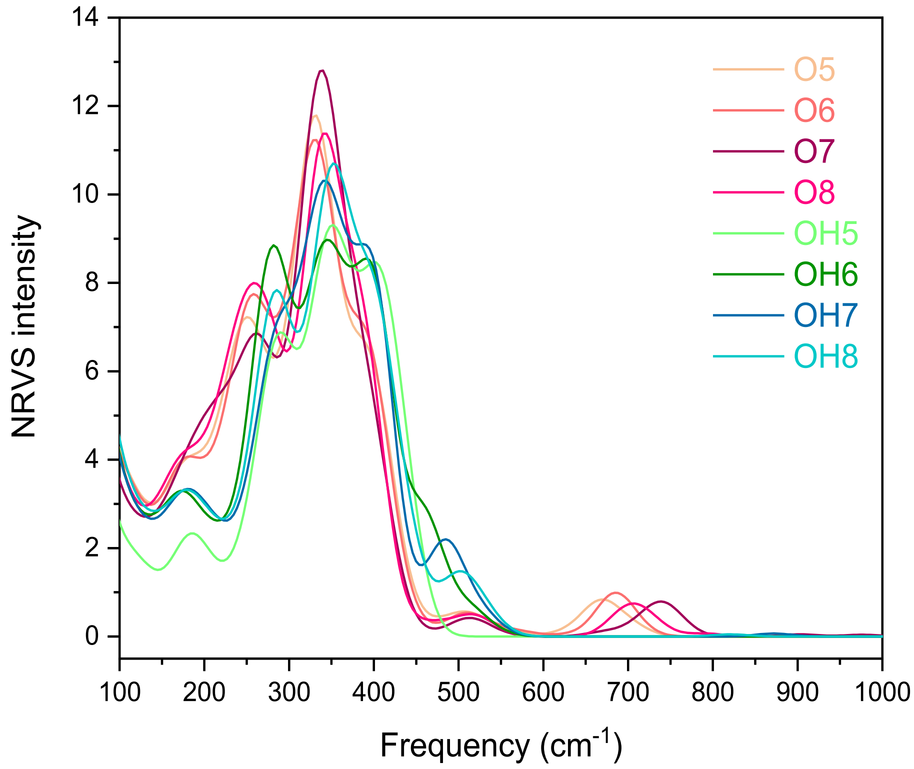

Figure S19. NRVS spectra of the **O5-O8** and **OH5-OH8** of APX-II computed with r^{2}SCAN/def2-TZVP (T = 298.15 K).

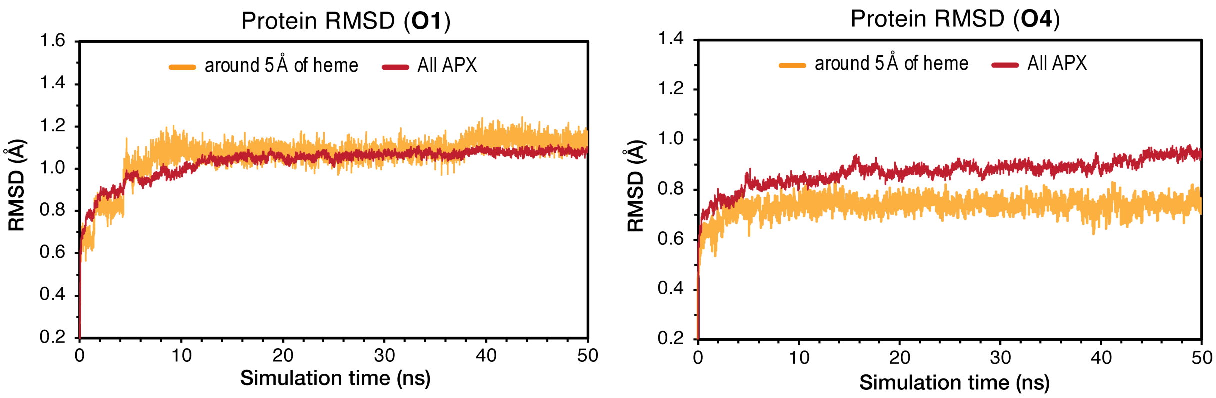

Figure S20. Time evolution of the root-mean-square deviation (RMSD, in Å) of the protein atoms of the complete APX-II protein and within a 5 Å region around the heme cofactor, shown along the MD trajectory.

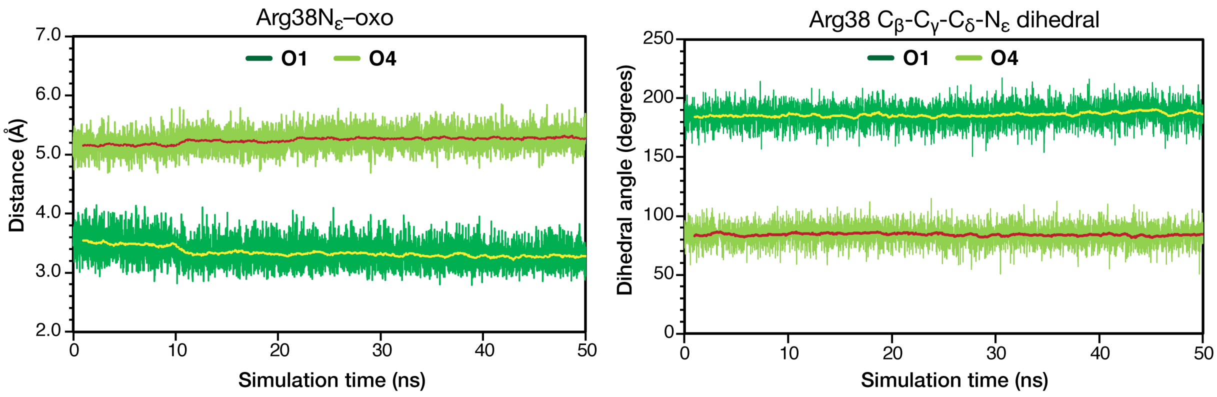

Figure S21. Time evolution of the Arg38–Fe(oxo) distances (left) and of the Arg38 C_β-C_γ-C_δ-N_ε dihedral angle along the production MD run for the **O1** and **O4** active site configurations.

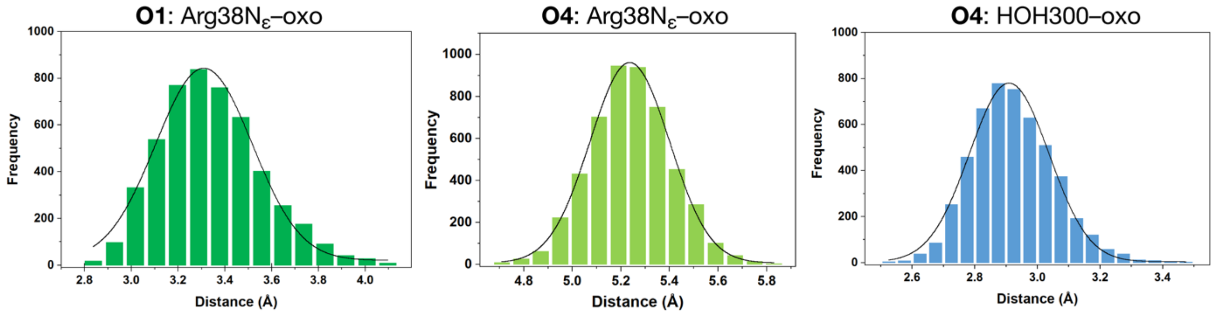

Figure S22. Frequency distribution of selected important distances with respect to the oxo group in the **O1** and **O4** models, sampled throughout the production MD runs.

Further details of calculations

Plotting of spectra

The generated output files were used together with the command *orca_mapspc*, as shown below:

TD-DFT: `orca_mapspc filename.out -i`

XAS: `orca_mapspc filename.out ABSQ -eV -x06500 -x17500 -n10001 -w2.0`

XES: `orca_mapspc filename.out XESQ -eV -x06500 -x17500 -n10001 -w2.0`

NRVS: `orca_vib filename.hess > outputname.out; orca_mapspc outputname.out NRVS`

Example orca input files for spectroscopic calculations

The following input files depict usual settings employed in the present work. Note, however, that these input files are not the ones used in actual QM/MM calculations, which require information on the complete protein structure, force field parameters, and definition of the QM and active regions. Examples of such "complete" input files are provided separately for the case of an oxo model (**O4**), along with additional files required to fully reproduce the QM/MM calculations.

(i) Mössbauer

```
!UKS B3LYP def2-TZVP RIJCOSX def2/J
!NoTrah TightSCF D3BJ SlowConv
!MORead
%moinp "filename.gbw"
%scf MaxIter 500 end
%basis
newgto Fe "CP(PPP)" end
end
%method
SpecialGridAtoms 26
SpecialGridIntAcc 7
end
%pal nprocs 16 end
%maxcore 12000
*xyzfile 0 2 filename.xyz
```

(ii) TD-DFT

```
!UKS LC-BLYP def2-TZVP RIJCOSX def2/J
!NoTrah TightSCF SlowConv
!MORead
%moinp "filename.gbw"
```

```
%scf MaxIter 500 end
%basis
newgto Fe "CP(PPP)" end
end
%method
SpecialGridAtoms 26
SpecialGridIntAcc 7
end
% tddft NRoots 150
        MaxDim 8
        DoNTO True
end
%pal nprocs 16 end
%maxcore 12000
*xyzfile 0 2 filename.xyz
```

(iii) XAS

```
!UKS B3LYP def2-TZVP RIJCOSX def2/J
!NoTrah TightSCF D3BJ SlowConv LARGEPRINT
!MORead
%moinp "filename.gbw"
%scf MaxIter 500 end
%basis
newgto Fe "CP(PPP)" end
end
%method
SpecialGridAtoms 26
SpecialGridIntAcc 7
end
% tddft NRoots 150
        MaxDim 8
        OrbWin[0]=0,0,-1,-1
        OrbWin[1]=0,0,-1,-1
        DoQuad true
end
%pal nprocs 16 end
%maxcore 12000
*xyzfile 0 2 filename.xyz
```

(iv) XES

```
!UKS B3LYP def2-TZVP RIJCOSX def2/J
!NoTrah TightSCF D3BJ SlowConv LARGEPRINT
!MORead
%moinp "filename.gbw"
%scf MaxIter 500 end
%basis
newgto Fe "CP(PPP)" end
end
%method
```

```
SpecialGridAtoms 26
SpecialGridIntAcc 7
end
%xes
CoreOrb 0,0
OrbOp 0,1
DoQuad true
CoreOrbSOC 0,1
DoSOC true
end
%pal nprocs 16 end
%maxcore 20000
*xyzfile 0 2 filename.xyz
```

(v) NRVS

```
!UKS r2SCAN def2-TZVP RI def2/J
!NoTrah TightSCF D4 KDIIS
!NUMFREQ
%scf maxiter 500
     directresetfreq 1
     CNVSOSCF true SOSCFStart 0.001
     end
%pal nprocs 24 end
%maxcore 30000
*xyzfile 0 2 filename.xyz
```

MK-RESP charges for heme in Fe(IV)=O

1	CAA	36.3724	45.4276	38.7169	CT	2763	HEM	0.306937
2	CAB	38.9630	36.6098	35.3318	CY	2763	HEM	-0.076162
3	CAC	44.0402	38.5280	41.1643	CY	2763	HEM	-0.033685
4	CAD	39.2609	44.4973	43.1298	CT	2763	HEM	0.098815
5	NA	38.0819	42.1362	38.4085	NP	2763	HEM	-0.113706
6	CBA	37.3981	46.5102	38.3676	CT	2763	HEM	-0.083079
7	CBB	39.2604	35.5121	36.0378	CX	2763	HEM	-0.410538
8	CBC	44.6300	37.3971	41.5626	CX	2763	HEM	-0.444318
9	CBD	39.7447	45.8662	42.6607	CT	2763	HEM	-0.038774
10	NB	39.0579	39.7384	37.3023	NO	2763	HEM	-0.336373
11	CGA	36.9289	47.9581	38.5968	C	2763	HEM	0.608620
12	CGD	39.3969	47.0218	43.5878	C	2763	HEM	0.678835
13	ND	39.9490	41.8960	40.5278	NO	2763	HEM	-0.237873
14	CHA	38.2807	43.6558	40.3121	CD	2763	HEM	-0.296132
15	CHB	37.3989	41.1994	36.2628	CD	2763	HEM	-0.085680
16	CHC	40.6367	37.9167	37.6148	CD	2763	HEM	-0.171846
17	CHD	41.8358	40.6044	41.3979	CD	2763	HEM	-0.060346
18	CMA	35.5960	43.7017	36.0968	CT	2763	HEM	-0.407558
19	CMB	37.1506	38.9764	34.2068	CT	2763	HEM	-0.247150
20	CMC	43.3015	36.8189	38.4780	CT	2763	HEM	-0.356661
21	CMD	41.6090	42.4240	43.8264	CT	2763	HEM	-0.485941
22	C1A	37.7792	43.2858	39.0770	CC	2763	HEM	0.110816
23	C1B	38.1571	40.0438	36.3170	CC	2763	HEM	0.028926
24	C1C	41.3364	38.4571	38.6700	CC	2763	HEM	-0.074042
25	C1D	40.8984	41.6145	41.4730	CC	2763	HEM	-0.161617
26	O1A	35.7281	48.2341	38.3444	O2	2763	HEM	-0.703624
27	O1D	39.7917	48.1608	43.1926	O2	2763	HEM	-0.685779
28	C2A	36.8334	44.0737	38.3200	CB	2763	HEM	-0.295071
29	C2B	38.0551	38.9637	35.3789	CB	2763	HEM	0.101756
30	C2C	42.5542	37.8890	39.1796	CB	2763	HEM	0.180124
31	C2D	40.7859	42.5130	42.5926	CB	2763	HEM	0.348624
32	O2A	37.8140	48.7692	38.9625	O2	2763	HEM	-0.656286
33	O2D	38.7874	46.7924	44.6532	O2	2763	HEM	-0.722126
34	C3A	36.5544	43.3634	37.1826	CB	2763	HEM	0.261608
35	C3B	38.8666	37.9574	35.8477	CB	2763	HEM	-0.091230
36	C3C	42.8925	38.6298	40.2823	CB	2763	HEM	-0.045956
37	C3D	39.7644	43.3715	42.2993	CB	2763	HEM	-0.297641
38	C4A	37.3597	42.1668	37.2455	CC	2763	HEM	-0.175101
39	C4B	39.5407	38.4958	37.0099	CC	2763	HEM	0.132632
40	C4C	41.8859	39.6671	40.3886	CC	2763	HEM	-0.040528
41	C4D	39.2629	42.9793	41.0035	CC	2763	HEM	0.210094
42	NC	40.9650	39.5593	39.3877	NP	2763	HEM	-0.166550
43	FE	39.5727	40.8982	38.8398	FE	2763	HEM	1.111252
44	HAA1	36.2067	45.4427	39.7958	HC	2763	HEM	-0.063762
45	HAA2	35.4268	45.6682	38.2327	HC	2763	HEM	-0.063762
46	HAB	38.7065	36.4931	34.2810	HC	2763	HEM	0.108423
47	HAC	44.4401	39.4729	41.5277	HC	2763	HEM	0.116175
48	HAD1	39.5995	44.3421	44.1529	HC	2763	HEM	-0.007158
49	HAD2	38.1712	44.5020	43.1568	HC	2763	HEM	-0.007158
50	HBA1	37.6542	46.4347	37.3011	HC	2763	HEM	0.008995
51	HBA2	38.3268	46.3485	38.9222	HC	2763	HEM	0.008995
52	HBB1	39.2672	34.5298	35.5860	HC	2763	HEM	0.135721
53	HBB2	39.4599	35.5473	37.1016	HC	2763	HEM	0.135721
54	HBC1	45.4883	37.4131	42.2226	HC	2763	HEM	0.146482
55	HBC2	44.2416	36.4290	41.2753	HC	2763	HEM	0.146482
56	HBD1	39.3282	46.1131	41.6780	HC	2763	HEM	-0.002862
57	HBD2	40.8354	45.8654	42.5448	HC	2763	HEM	-0.002862

58	HHA	37.8774	44.5522	40.7685	HC	2763	HEM	0.245729
59	HHB	36.7752	41.3484	35.3920	HC	2763	HEM	0.085237
60	HHC	41.0103	36.9963	37.1930	HC	2763	HEM	0.116994
61	HHD	42.5595	40.5303	42.1992	HC	2763	HEM	0.061969
62	HMA1	36.0956	43.8997	35.1446	HC	2763	HEM	0.105381
63	HMA2	35.0151	44.5855	36.3592	HC	2763	HEM	0.105381
64	HMA3	34.8866	42.8858	35.9323	HC	2763	HEM	0.105381
65	HMB1	37.1077	38.0089	33.7083	HC	2763	HEM	0.062926
66	HMB2	37.4526	39.7317	33.4758	HC	2763	HEM	0.062926
67	HMB3	36.1449	39.2207	34.5458	HC	2763	HEM	0.062926
68	HMC1	44.2844	36.6783	38.9266	HC	2763	HEM	0.092664
69	HMC2	43.4517	37.0910	37.4293	HC	2763	HEM	0.092664
70	HMC3	42.7536	35.8712	38.4947	HC	2763	HEM	0.092664
71	HMD1	41.3859	41.5076	44.3803	HC	2763	HEM	0.123561
72	HMD2	41.4056	43.2671	44.4821	HC	2763	HEM	0.123561
73	HMD3	42.6768	42.4397	43.5939	HC	2763	HEM	0.123561

MK-RESP charges for heme in Fe(III)-OH

1	CAA	17.6240	-1.0600	49.0770	CT	2763	HEM	-0.112611
2	CAB	23.5940	-1.6890	41.0150	CY	2763	HEM	-0.043182
3	CAC	17.9000	-7.2380	40.1130	CY	2763	HEM	0.031110
4	CAD	15.2900	-5.7890	47.6310	CT	2763	HEM	-0.120488
5	NA	18.9530	-2.3090	45.7160	NP	2763	HEM	-0.232824
6	CBA	16.3200	-0.2710	48.8510	CT	2763	HEM	0.011680
7	CBB	24.9350	-1.7160	41.4050	CX	2763	HEM	-0.458804
8	CBC	18.5150	-8.4220	39.7260	CX	2763	HEM	-0.486050
9	CBD	16.2330	-6.8030	48.3310	CT	2763	HEM	0.017371
10	NB	20.7250	-2.4190	43.3760	NO	2763	HEM	-0.179232
11	CGA	15.7090	0.2830	50.1310	C	2763	HEM	0.649022
12	CGD	15.6440	-7.1420	49.6770	C	2763	HEM	0.646879
13	ND	17.5000	-4.5490	44.8870	NO	2763	HEM	-0.288067
14	CHA	17.2380	-3.4410	47.0130	CD	2763	HEM	-0.252463
15	CHB	20.6830	-0.6660	45.0890	CD	2763	HEM	-0.032740
16	CHC	21.3040	-3.7490	41.4220	CD	2763	HEM	-0.216851
17	CHD	17.2990	-6.0610	42.9380	CD	2763	HEM	-0.166732
18	CMA	19.9630	0.7730	47.8110	CT	2763	HEM	-0.072224
19	CMB	23.1280	0.5230	43.5680	CT	2763	HEM	-0.384037
20	CMC	20.5150	-5.7100	39.0600	CT	2763	HEM	-0.333470
21	CMD	15.2610	-7.6330	44.8020	CT	2763	HEM	-0.061726
22	C1A	18.1320	-2.4300	46.8290	CC	2763	HEM	0.113575
23	C1B	21.1930	-1.2800	43.9580	CC	2763	HEM	-0.152357
24	C1C	20.1910	-4.5950	41.4770	CC	2763	HEM	-0.086290
25	C1D	17.0230	-5.6820	44.2430	CC	2763	HEM	0.004734
26	O1A	16.3000	0.1380	51.2450	O2	2763	HEM	-0.607320
27	O1D	15.5510	-6.2590	50.5570	O2	2763	HEM	-0.607799
28	C2A	18.3330	-1.2880	47.7230	CB	2763	HEM	-0.023728
29	C2B	22.3340	-0.7870	43.1730	CB	2763	HEM	0.235395
30	C2C	19.8050	-5.5270	40.4400	CB	2763	HEM	0.201141
31	C2D	16.0730	-6.3540	45.1150	CB	2763	HEM	0.035952
32	O2A	14.6450	0.9780	50.0260	O2	2763	HEM	-0.618511
33	O2D	15.2520	-8.3200	49.9060	O2	2763	HEM	-0.622558
34	C3A	19.3160	-0.5350	47.1930	CB	2763	HEM	0.047158
35	C3B	22.5200	-1.6670	42.1610	CB	2763	HEM	-0.114934
36	C3C	18.7170	-6.1710	40.8900	CB	2763	HEM	-0.157559
37	C3D	16.0400	-5.5020	46.3340	CB	2763	HEM	-0.019910
38	C4A	19.7240	-1.1630	45.9600	CC	2763	HEM	-0.077547
39	C4B	21.4940	-2.6840	42.2790	CC	2763	HEM	0.048133
40	C4C	18.3550	-5.6200	42.1630	CC	2763	HEM	0.062374

41	C4D	16.9770	-4.4270	46.1320	CC	2763	HEM	0.115457
42	NC	19.2860	-4.6710	42.5300	NP	2763	HEM	-0.198402
43	FE	19.1350	-3.4790	44.1200	FE	2763	HEM	0.576908
44	HAA1	18.2790	-0.5130	49.7540	HC	2763	HEM	0.031805
45	HAA2	17.4040	-2.0170	49.5500	HC	2763	HEM	0.031805
46	HAB	23.3060	-1.6810	39.9630	HC	2763	HEM	0.094540
47	HAC	16.8510	-7.0600	39.8730	HC	2763	HEM	0.069710
48	HAD1	14.3020	-6.2110	47.4470	HC	2763	HEM	0.033377
49	HAD2	15.1450	-4.8880	48.2270	HC	2763	HEM	0.033377
50	HBA1	15.5940	-0.9210	48.3630	HC	2763	HEM	-0.010550
51	HBA2	16.5200	0.5540	48.1670	HC	2763	HEM	-0.010550
52	HBB1	25.7210	-1.7320	40.6500	HC	2763	HEM	0.131182
53	HBB2	25.1940	-1.7220	42.4640	HC	2763	HEM	0.131182
54	HBC1	17.9490	-9.1770	39.1800	HC	2763	HEM	0.143428
55	HBC2	19.5630	-8.5960	39.9680	HC	2763	HEM	0.143428
56	HBD1	17.2290	-6.3760	48.4490	HC	2763	HEM	-0.008935
57	HBD2	16.3410	-7.7020	47.7260	HC	2763	HEM	-0.008935
58	HHA	16.6910	-3.4540	47.9450	HC	2763	HEM	0.136824
59	HHB	21.0760	0.3140	45.3200	HC	2763	HEM	0.074424
60	HHC	22.0540	-3.9390	40.6700	HC	2763	HEM	0.116931
61	HHD	16.6220	-6.7690	42.4820	HC	2763	HEM	0.111744
62	HMA1	19.2110	1.3180	48.3800	HC	2763	HEM	0.026028
63	HMA2	20.3400	1.4070	47.0080	HC	2763	HEM	0.026028
64	HMA3	20.7860	0.4950	48.4700	HC	2763	HEM	0.026028
65	HMB1	24.1640	0.4310	43.2450	HC	2763	HEM	0.099556
66	HMB2	23.0950	0.6560	44.6500	HC	2763	HEM	0.099556
67	HMB3	22.6720	1.3860	43.0830	HC	2763	HEM	0.099556
68	HMC1	20.9600	-4.7650	38.7520	HC	2763	HEM	0.079660
69	HMC2	19.7860	-6.0290	38.3160	HC	2763	HEM	0.079660
70	HMC3	21.2960	-6.4650	39.1500	HC	2763	HEM	0.079660
71	HMD1	15.0750	-8.1810	45.7260	HC	2763	HEM	0.023669
72	HMD2	15.8250	-8.2630	44.1140	HC	2763	HEM	0.023669
73	HMD3	14.3100	-7.3570	44.3460	HC	2763	HEM	0.023669

Additional models/variants not discussed in the main text

Table S20. Bond lengths and relative energies within ground state (S = 1) APX-II models with singly protonated His42 (**O1–O4**, **O9–O35**) and those with doubly protonated His42 (**O5–O8, O36–O37**), calculated using the r^{2}SCAN-D4/def2-TZVP level of theory.

Fe(IV)-oxo models	Fe-O (Å)	ΔE (kJ/mol)
**O1**	1.671	**0.0**
**O2**	1.674	-23.3
**O3**	1.659	25.7
**O4**	1.669	-11.5
**O9**	1.666	12.4
**O10**	1.671	0.3
**O11**	1.668	-19.2
**O12**	1.661	37.6
**O13**	1.661	18.7
**O14**	1.668	1.5
**O15**	1.661	18.5
**O16**	1.651	51.0
**O17**	1.669	1.5
**O18**	1.670	0.9
**O19**	1.671	0.1
**O20**	1.671	0.2
**O21**	1.659	28.7
**O22**	1.671	-0.2
**O23**	1.665	37.8
**O24**	1.663	38.1
**O25**	1.668	1.5
**O26**	1.664	19.9
**O27**	1.656	-3.1
**O28**	1.664	-26.8
**O29**	1.672	-23.0
**O30**	1.663	15.5
**O31**	1.651	50.9
**O32**	1.661	18.6
**O33**	1.666	19.7
**O34**	1.661	37.1
**O35**	1.667	20.8
Doubly protonated His42:		
**O5**	1.714	**0.0**
**O6**	1.711	-62.1
**O7**	1.678	20.1
**O8**	1.699	-46.1
**O36**	1.685	-4.8
**O37**	1.695	-28.3

Table S21. Bond lengths and relative energies within ground state (S = 1) APX-II models with singly protonated His42 (**OH1–OH4**, **OH9–OH37**) and those with doubly protonated His42 (**OH5–OH8**), calculated using the r^{2}SCAN-D4/def2-TZVP level of theory.

Fe(IV)-hydroxo models	Fe-O (Å)	Δ*E* (kJ/mol)
**OH1**	1.798	**0.0**
**OH2**	1.824	-23.1
**OH3**	1.829	6.6
**OH4**	1.811	-26.3
**OH9**	1.833	-2.1
**OH10**	1.798	0.1
**OH11**	1.825	-23.1
**OH12**	1.835	-25.8
**OH13**	1.825	-23.5
**OH14**	1.797	0.2
**OH15**	1.842	-32.8
**OH16**	1.809	-5.9
**OH17**	1.798	0.1
**OH18**	1.798	0.5
**OH19**	1.819	-4.9
**OH20**	1.827	7.7
**OH21**	1.846	-8.8
**OH22**	1.798	0.1
**OH23**	1.797	-0.2
**OH24**	1.798	0.1
**OH25**	1.832	-2.1
**OH26**	1.804	-6.1
**OH27**	1.804	-2.8
**OH28**	1.810	-26.1
**OH29**	1.796	-5.3
**OH30**	1.820	-42.4
**OH31**	1.847	-8.5
**OH32**	1.812	-3.0
**OH33**	1.852	7.5
**OH34**	1.838	28.9
**OH35**	1.793	17.0
**OH36**	1.792	16.9
**OH37**	1.793	-14.7
Doubly protonated His42:		
**OH5**	1.932	**0.0**
**OH6**	1.906	-31.3
**OH7**	1.898	-15.6
**OH8**	1.915	-34.6

O9

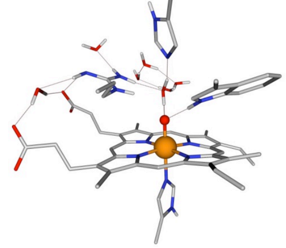

O10

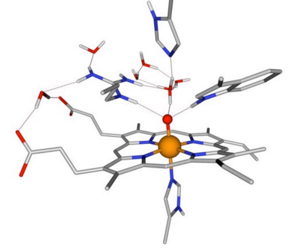

O11

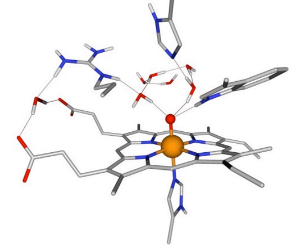

O12

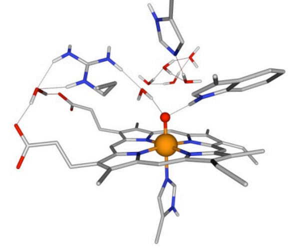

O13

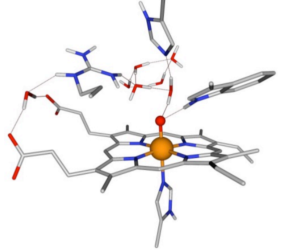

O14

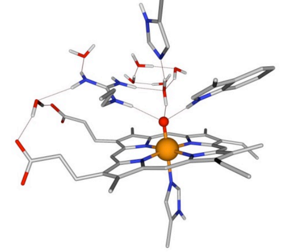

O15

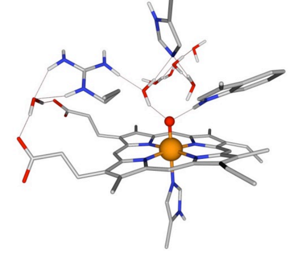

O16

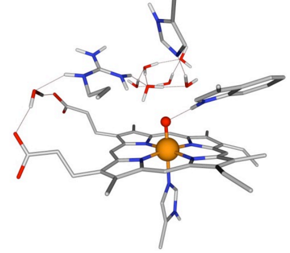

O17

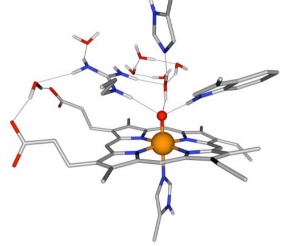

O18

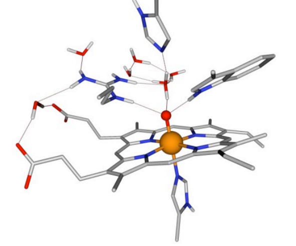

O19

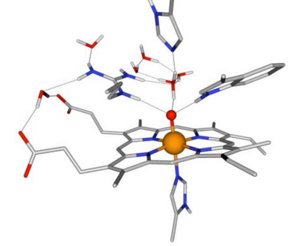

O20

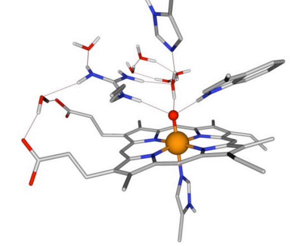

O21

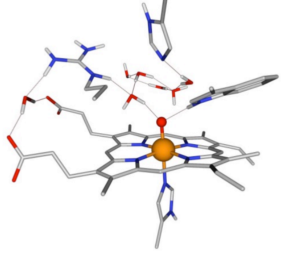

O22

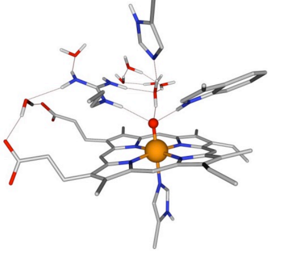

O23

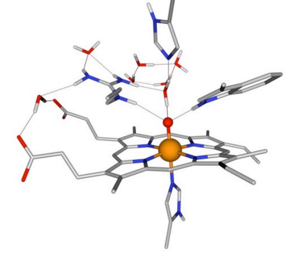

O24

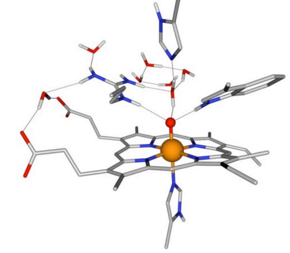

O25

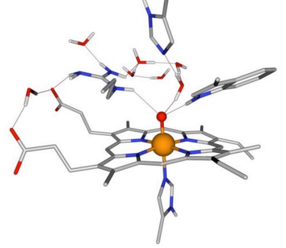

O26

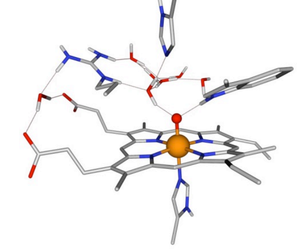

O27

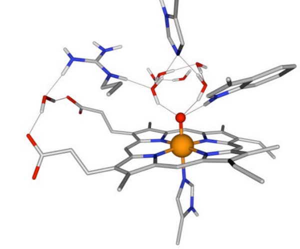

O28

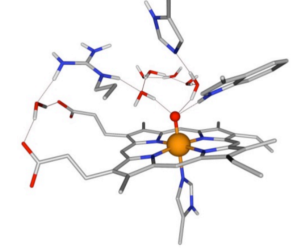

O29

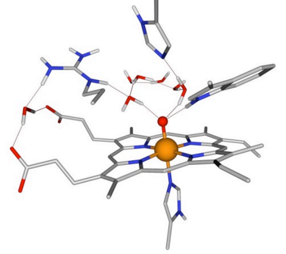

O30

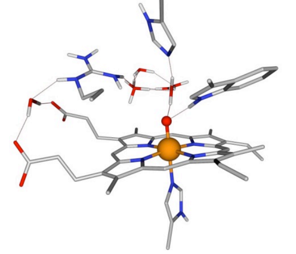

O31

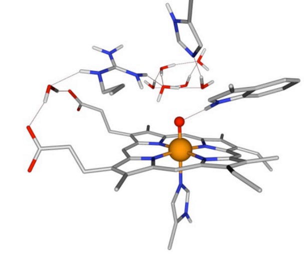

O32

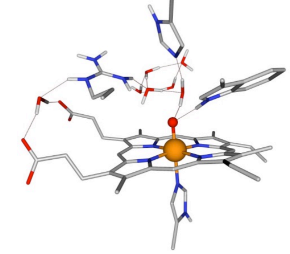

O33

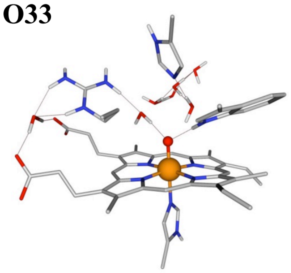

O34

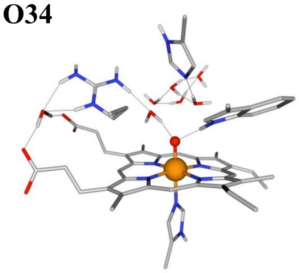

O35

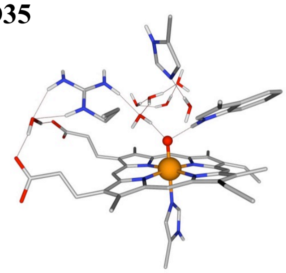

O36

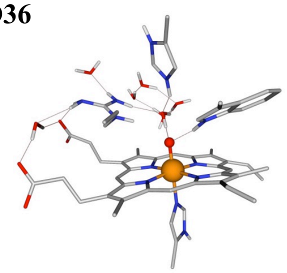

O37

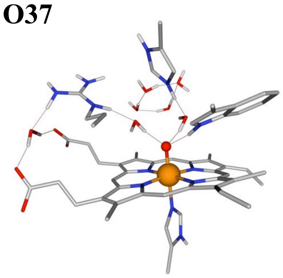

Figure S23. Optimized structures (QM regions) for models **O9-O37**.

OH9
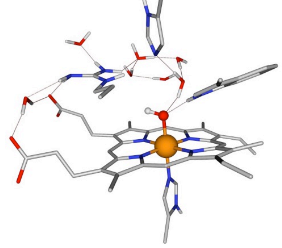
OH10
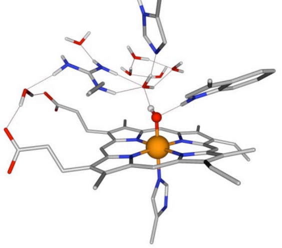
OH11
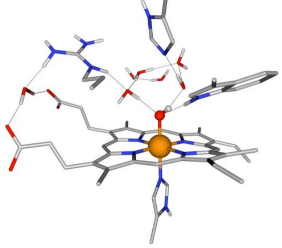
OH12
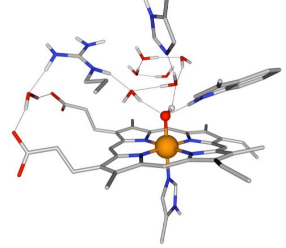
OH13
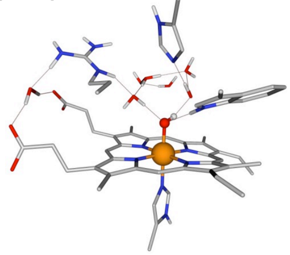
OH14
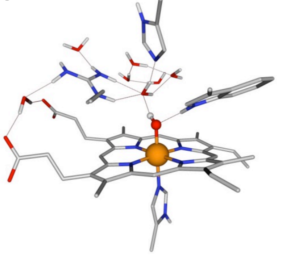
OH15
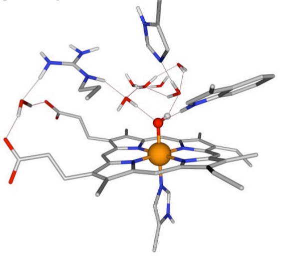
OH16
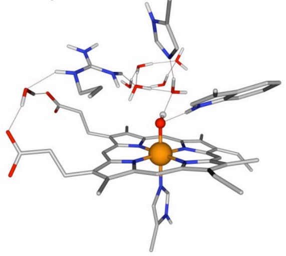
OH17
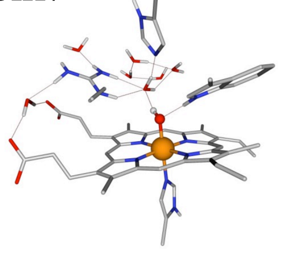
OH18
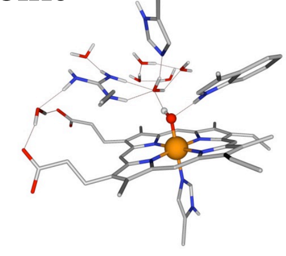
OH19
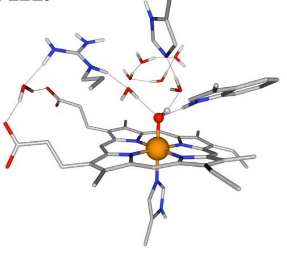
OH20
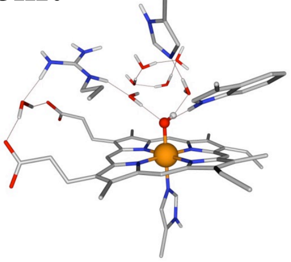
OH21
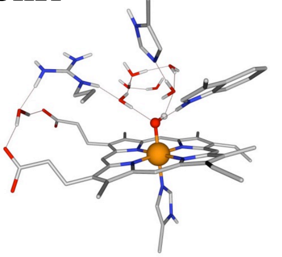
OH22
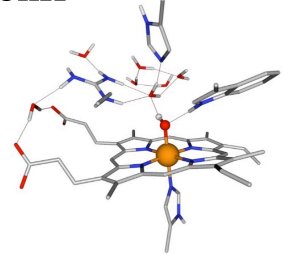
OH23
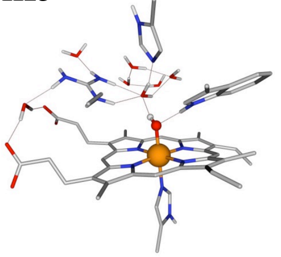
OH24
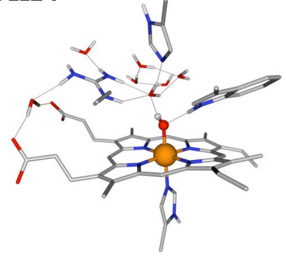
OH25
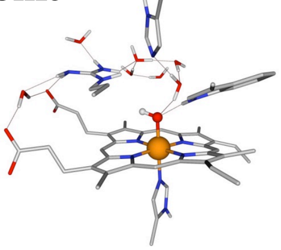
OH26
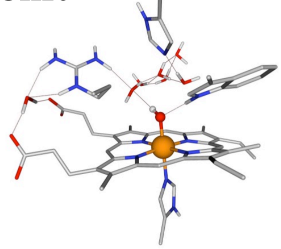
OH27
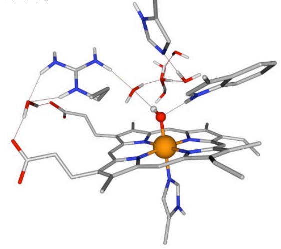
OH28
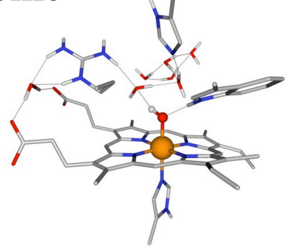
OH29
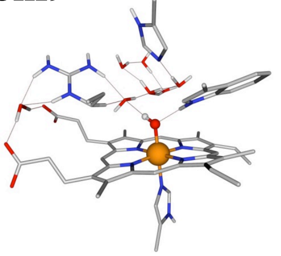
OH30
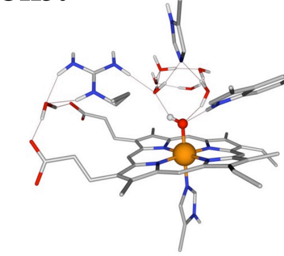
OH31
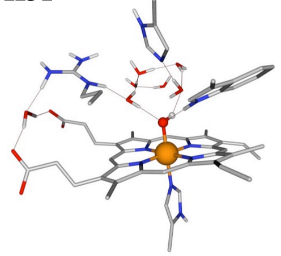
OH32
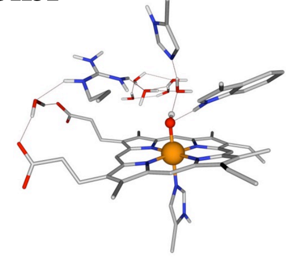

OH33

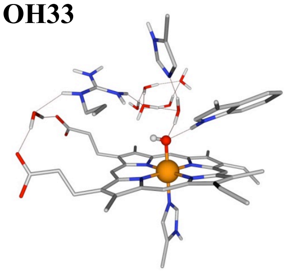

OH34

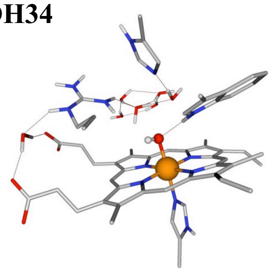

OH35

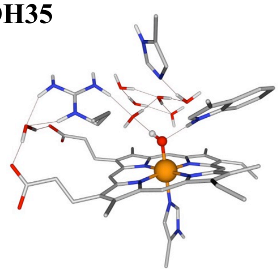

OH36

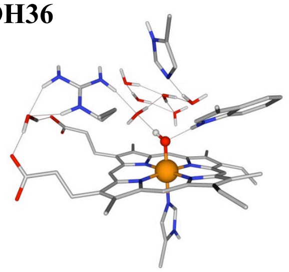

OH37

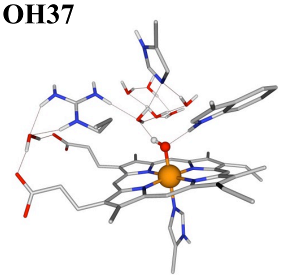

Figure S24. Optimized structures (QM regions) of models **OH9-OH37**.

References

(1) Ledray, A. P.; Krest, C. M.; Yosca, T. H.; Mittra, K.; Green, M. T. Ascorbate Peroxidase Compound II Is an Iron(IV) Oxo Species. *J. Am. Chem. Soc.* **2020**, *142*, 20419-20425.
(2) Kwon, H.; Basran, J.; Pathak, C.; Hussain, M.; Freeman, S. L.; Fielding, A. J.; Bailey, A. J.; Stefanou, N.; Sparkes, H. A.; Tosha, T.; Yamashita, K.; Hirata, K.; Murakami, H.; Ueno, G.; Ago, H.; Tono, K.; Yamamoto, M.; Sawai, H.; Shiro, Y.; Sugimoto, H.; Raven, E. L.; Moody, P. C. E. XFEL Crystal Structures of Peroxidase Compound II. *Angew. Chem., Int. Ed.* **2021**, *60*, 14578-14585.
(3) Kwon, H.; Basran, J.; Casadei, C. M.; Fielding, A. J.; Schrader, T. E.; Ostermann, A.; Devos, J. M.; Aller, P.; Blakeley, M. P.; Moody, P. C. E.; Raven, E. L. Direct visualization of a Fe(IV)–OH intermediate in a heme enzyme. *Nat. Commun.* **2016**, *7*, 13445.
(4) Gumiero, A.; Metcalfe, C. L.; Pearson, A. R.; Raven, E. L.; Moody, P. C. E. Nature of the Ferryl Heme in Compounds I and II. *J. Biol. Chem.* **2011**, *286*, 1260-1268.
(5) Kwon, H.; Basran, J.; Devos, J. M.; Suardíaz, R.; van der Kamp, M. W.; Mulholland, A. J.; Schrader, T. E.; Ostermann, A.; Blakeley, M. P.; Moody, P. C. E.; Raven, E. L. Visualizing the protons in a metalloenzyme electron proton transfer pathway. *Proc. Natl. Acad. Sci. U. S. A.* **2020**, *117*, 6484-6490.
(6) Ledray, A. P.; Mittra, K.; Green, M. T. NRVS investigation of ascorbate peroxidase compound II: Observation of Iron(IV)oxo stretching. *J. Inorg. Biochem.* **2021**, *224*, 111548.
